# The earliest Ethiopian wolf: implications for the species evolution and its future survival

**DOI:** 10.1038/s42003-023-04908-w

**Published:** 2023-05-16

**Authors:** Bienvenido Martínez-Navarro, Tegenu Gossa, Francesco Carotenuto, Saverio Bartolini-Lucenti, Paul Palmqvist, Asfawossen Asrat, Borja Figueirido, Lorenzo Rook, Elizabeth M. Niespolo, Paul R. Renne, Gadi Herzlinger, Erella Hovers

**Affiliations:** 1 ICREA, Pg. Lluís Companys 23, 08010 Barcelona, Spain; 2 Institut Català de Paleoecologia Humana i Evolució Social (IPHES-CERCA), Zona Educacional 4, Campus Sescelades URV (Edifici W3), 43007 Tarragona, Spain; 3 Universitat Rovira i Virgili, Departament d’Història i Història de l’Art, Avinguda de Catalunya 35, 43002 Tarragona, Spain; 4 Human Evolution Research Center (HERC), The University of California at Berkeley, Berkeley, CA USA; 5 Institute of Archaeology, The Hebrew University of Jerusalem, Jerusalem, Israel; 6 Department of History and Heritage Management, Arba Minch University, Arba Minch, Ethiopia; 7 Department of Earth, Environment and Resource Sciences, University of Naples “Federico II”, Naples, Italy; 8 Earth Science Department, Paleo[Fab]Lab, University of Florence, Via G. La Pira 4, Firenze, 50121 Italy; 9 Institut Català de Paleontogia M. Crusafont, Universitat Autònoma de Barcelona, E-08193 Cerdanyola del Vallès, Spain; 10 Departamento de Ecología y Geología, Universidad de Málaga, Universidad de Málaga, Campus de Teatinos, 29071 Málaga, Spain; 11 Department of Mining and Geological Engineering, Botswana International University of Science and Technology, Private Bag 16, Palapye, Botswana; 12 School of Earth Sciences, Addis Ababa University, P. O. Box 1176 Addis Ababa, Ethiopia; 13 Department of Geosciences, Princeton University, Princeton, NJ USA; 14 Department of Earth and Planetary Science, University of California, Berkeley, CA USA; 15 Berkeley Geochronology Center, Berkeley, CA USA; 16 Institute of Human Origins, Arizona State University, Tempe, USA

**Keywords:** Palaeontology, Conservation biology

## Abstract

In 2017, a hemimandible (MW5-B208), corresponding to the Ethiopian wolf (*Canis simensis*), was found in a stratigraphically-controlled and radio-isotopically-dated sequence of the Melka Wakena paleoanthropological site-complex, on the Southeastern Ethiopian Highlands, ~ 2300 m above sea level. The specimen is the first and unique Pleistocene fossil of this species. Our data provide an unambiguous minimum age of 1.6–1.4 Ma for the species’ presence in Africa and constitutes the first empirical evidence that supports molecular interpretations. Currently, *C. simensis* is one of the most endangered carnivore species of Africa. Bioclimate niche modeling applied to the time frame indicated by the fossil suggests that the lineage of the Ethiopian wolf faced severe survival challenges in the past, with consecutive drastic geographic range contractions during warmer periods. These models help to describe future scenarios for the survival of the species. Projections ranging from most pessimistic to most optimistic future climatic scenarios indicate significant reduction of the already-deteriorating territories suitable for the Ethiopian Wolf, increasing the threat to the specie’s future survival. Additionally, the recovery of the Melka Wakena fossil underscores the importance of work outside the East African Rift System in research of early human origins and associated biodiversity on the African continent.

## Abstract (web summary)

The Ethiopian wolf is one of Africa’s most endangered carnivores. A new fossil specimen indicates the presence of this species in Ethiopia since the Early Pleistocene, with climate modelling indicating variable past and future ranges for the species.

## Introduction

**Fig. 1:**
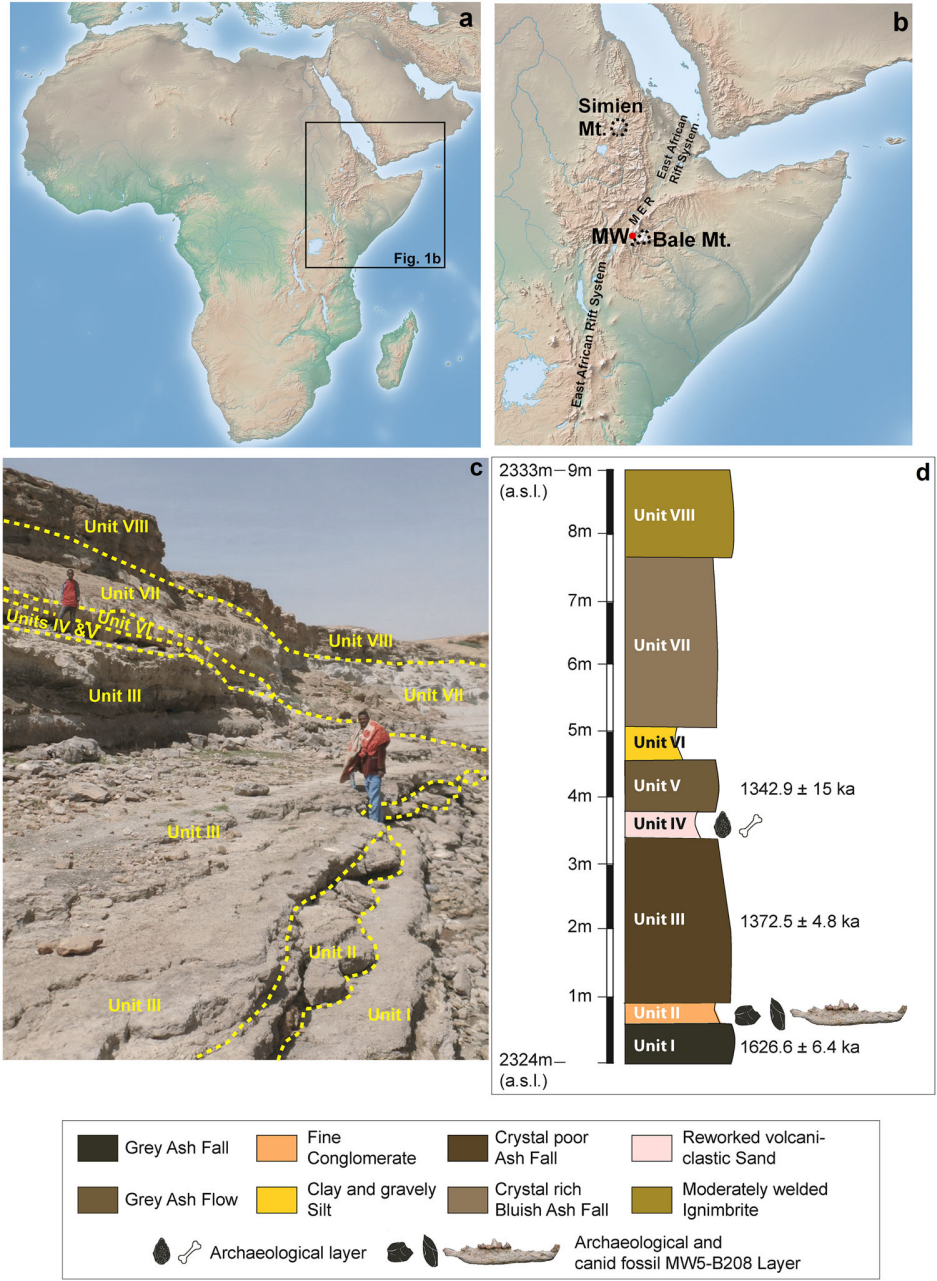
Location and stratigraphy of the site of Melka Wakena (Ethiopia). **a**, **b** Relief map from Natural Earth (public domain): http://www.naturalearthdata.com/. **c** Stratigraphy of the MW5-West sequence. Photo taken by author (T.G.) in 2021. **d** Stratigraphic position of MW5-B208 in relation to the dated archaeological sequence at locality MW5. Stratigraphic log was created by author (A.A.).

**Fig. 2:**
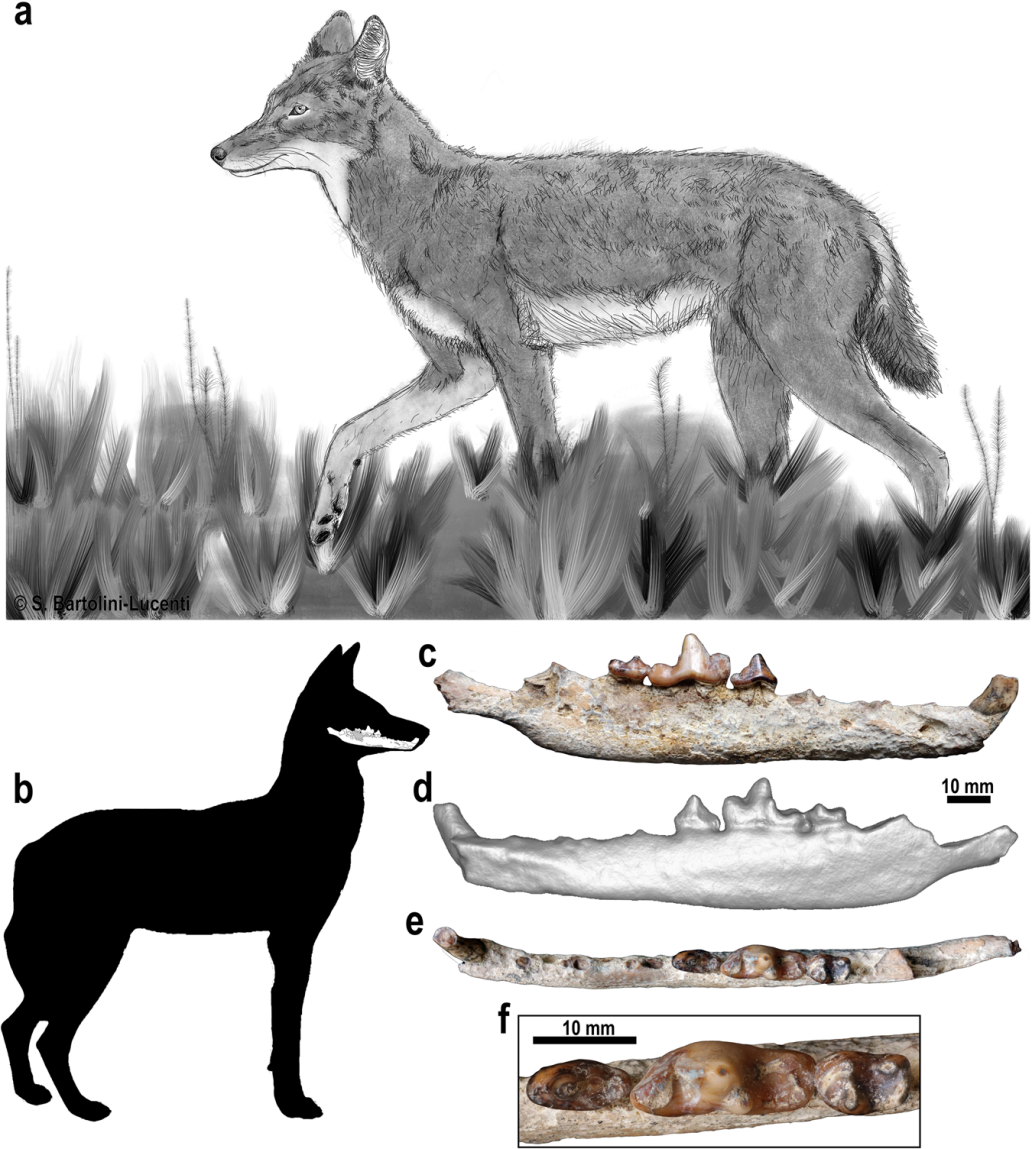
The fossil from Melka Wakena and the extant Ethiopian wolf. **a** *Canis simensis* drawing by S. Bartolini-Lucenti. **b** the silhouette of *C. simensis* and the photographs and 3D model of the right mandible MW5-B208 **c** in buccal, **d** lingual, **e** occlusal, and **f** detailed occlusal view of the p4-m2. Silhouette of *Canis simensis* made by authors (S.B.L.).

The Ethiopian wolf (*Canis simensis*) is an endemic species of the Ethiopian fauna. Its census population of only ~500 individuals, ~200 of them adults, is spread over six different isolated populations restricted to elevations over 3000 m above sea level in the Ethiopian highlands^1–8^. Given an estimated minimum viable population size of 3876 individuals, with a 95% confidence interval of 2261–5095 individuals^9^, *C. simensis* is currently one of the most endangered carnivores on the African continent. This animal and the large cercopithecid monkey *Theropithecus gelada* are the most iconic faunal taxa of Ethiopia. Although *Theropithecus* is now only present in the Ethiopian highlands, the Pleistocene fossil record of this lineage is well-known from eastern, southern and northern Africa, as well as in the Indian Subcontinent, the Levantine Corridor and the Iberian Peninsula^10^. In contrast, there is no Pleistocene fossil record of *C. simensis* from Ethiopia or elsewhere. Thus, the origin of this species and its time of arrival to Ethiopia remains unknown. Extant *C. simensis* is more closely linked genetically with the Eurasian and North American gray wolf, *Canis lupus*, the North American coyote, *C. latrans*, the Asian jackal *C. aureus*, or the African small golden wolf *C. lupaster*, than with any other African canid such as the African painted dog, *Lycaon pictus*, or the African jackals (*Lupulella mesomelas* and *Lup. adusta*) (Supp. Note 3, Supp. Figure 3). The absence of a fossil record of *C. simensis* led to the conclusion that the species arrived in the Ethiopian highlands during the Late Pleistocene^5,11,12^. The discovery of a fossil of *C. simensis* (MW5-B208) in a stratigraphically-controlled and radio-isotopically-dated context in the paleoanthropological site-complex of Melka Wakena^13^ undermines the latter hypothesis (Figs. 1 and 2). MW5-B208 represents the first—and (to date) the only—fossil of the Ethiopian wolf, testifying to its appearance and existence in its Afromontane habitat as early as the second half of the Early Pleistocene.

The Melka Wakena site-complex is located at ~2300 m above sea level (asl) (Supp. Figure 1) on the eastern shoulder of the Main Ethiopian Rift (MER) (Fig. 1a, b). Eight archaeological and two paleontological localities were identified to date, in a chronostratigraphic sequence corresponding to the second half of the Early Pleistocene, ~1.6 to >0.7 Ma^13^ (Fig. 1c, d; Supp. Notes 1 and 2, Supp. Figure 2). Currently, the vertebrate faunal list derived from the stratigraphic sequence consists of *Crocodylus* cf. *niloticus*, *Hystrix* sp., *Canis* sp., *Elephas recki* cf. *brumpti*, *Equus* sp., *Metridichoerus modestus*, *Hippopotamus gorgops*, *Giraffa* cf. *jumae*, *Tragelaphus* sp., Bovini indet. (Cf. *Pelorovis* sp.), *Aepyceros* cf. *melampus*, *Gazella* sp., Alcelaphini indet., Reduncini indet., and Antelopini indet. This fauna is consistent with a late Early Pleistocene age. A lower left m3 of the proboscidean *Elephas recki* cf. *brumpti*, a Pliocene form, was found in a secondary depositional context, embedded in sediments younger than 1.3 Ma^13^.

## Results

**Fig. 3:**
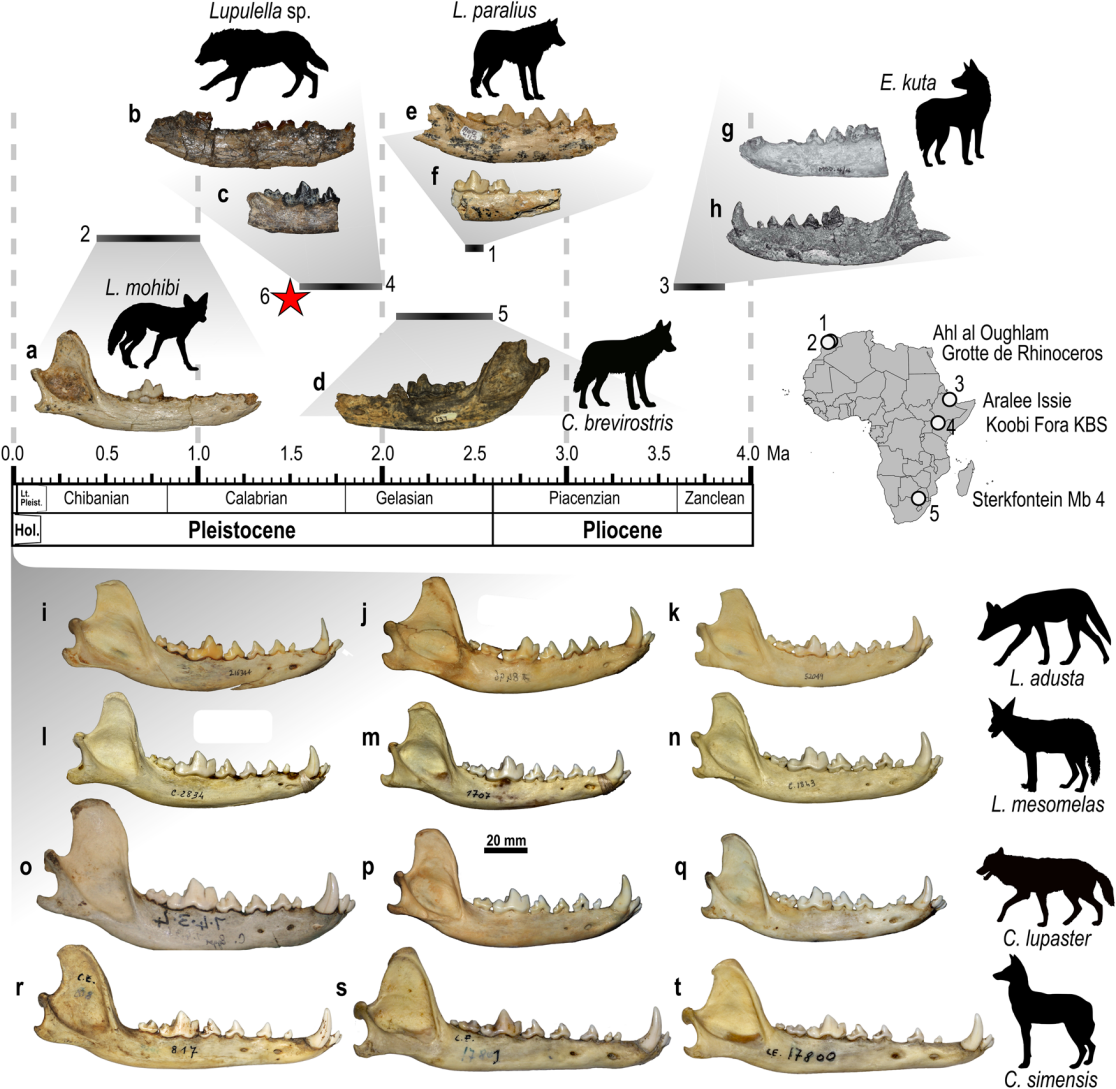
Buccal morphology of different Plio-Pleistocene and extant African canid mandibles. The fossil specimens are arranged in three lines in geographic areas (i.e., North, East, and South African sites). Site numeration: (**1**) Ahl al Oughlam (Morocco); (**2**) Grotte de Rhinoceros (Morocco); (**3**) Aralee Issie (Ethiopia); (**4**) Koobi Fora, Upper Burgi-KBS Members (Kenya); (**5**) Sterkfontein Member 4 (South Africa); (**6**) the red star, Melka Wakena (Ethiopia). **a** right mandible MNHN.F.Th1-10728 of *Lupulella mohibi* from Grotte de Rhinoceros^36^. **b**, **c** right mandible fragments of *Lupulella* sp. from Koobi Fora, Upper Burgi-KBS Members^37^: **b** KNM-ER 332, and **c** KNM-ER 895. **d** *Canis brevirostris* from Sterkfontein Member 4^39^: 3D model of left hemimandible fragment DNMNH.STS137 (courtesy of Dr. M. Tawane and J. Adams, see Adams et al.^40^). **e**, **f** buccal views of right mandible fragments of *Lupulella paralius* from Ahl al Oughlam^36^: **e** MNHN.F.AaO-4119, and **f** MNHN.F.AaO-3499. (**g**, **h**) left mandible fragments of *Eucyon kuta* from Aralee Issie^38^: **g** left hemimandible fragment MSD-VP-4/4^38^, and **h** left hemimandible ARI-VP-1/640^38^. **i**–**k** buccal views of mandibles of *Lupulella adusta*: **i** right mandible AMNH-216344, **j** left mandible, reversed, MZUF 8496, and **k** right mandible AMNH-52049. **l**–**n** buccal views of mandibles of *Lupulella mesomelas*: **l** right mandible MZUF 2834, **m** right mandible MZUF 1707, **n** right mandible MZUF 1843. **o**–**q** buccal views of mandibles of *Canis lupaster*: **o** right mandible NHM 7434, **p** left mandible, reversed, MZUF 1851, **q** right mandible MZUF 2110. **r**–**t** buccal views of mandibles of *Canis simensis*: **r** right mandible CE 818, **s** right mandible CE 17801, **t** right mandible CE 17800. Map and Silhouettes of canids made by authors (S.B.L.).

During the 2017 field season, a right hemimandible (MW5-B208) of a canid was found eroding out of a conglomerate layer (Unit II: Fig. 1c, d, Supp. Note 2, Supp. Figure 2) at the base of a cliff-section of MW5-West, ca. 90 meters west of the archaeological locality MW5. This conglomerate layer is found sandwiched between two volcanic ash layers. The underlying ash flow (Unit-I) is dated by single grain ^39^Ar/^40^Ar geochronology to 1626.6 ± 6.4 thousand years ago (ka) and the overlying ash flow layer (Unit III) dates to 1372.5 ± 4.8 ka (Fig. 1d; all errors are reported at 1 sigma, including systematic uncertainties)^13^. The fossil was initially classified as *Canis* sp.^13^. It clearly belongs to a medium-sized canid similar to the extant endemic Ethiopian *Canis simensis* (Figs. 2 and 3).

### Anatomical and morphometric comparisons with other extant and fossil canids

The dimensions and morphology of MW5-B208 are more similar to the extant *C. simensis* than to other extant African canids (both *Canis* and *Lupulella* species) or to various fossil canids of Africa (Fig. 3, Supp. Table 1, Supp. Note 4). The height of the corpus, the straight ventral margin, the position of the mental foramina (especially the distal one, under the diastema between p2-p3), the large diastemata between canine and premolars, and between premolars themselves are similar between MW5-B208 and the extant *C. simensis*. The shallowness and elongation of the mandible shows that MW5-B208 falls within the range of variability of the extant species *C. simensis*. The similarity extends to dental features: e.g., the oval shape of the p4, shortened in mesiodistal direction; the reduced distal accessory cuspulid on the p4; the slender m1 with reduced metaconid and entoconid; the similar-sized m2 protoconid and metaconid; and the squared distal shape of the m2 (Figs. 2 and 3).

**Fig. 4:**
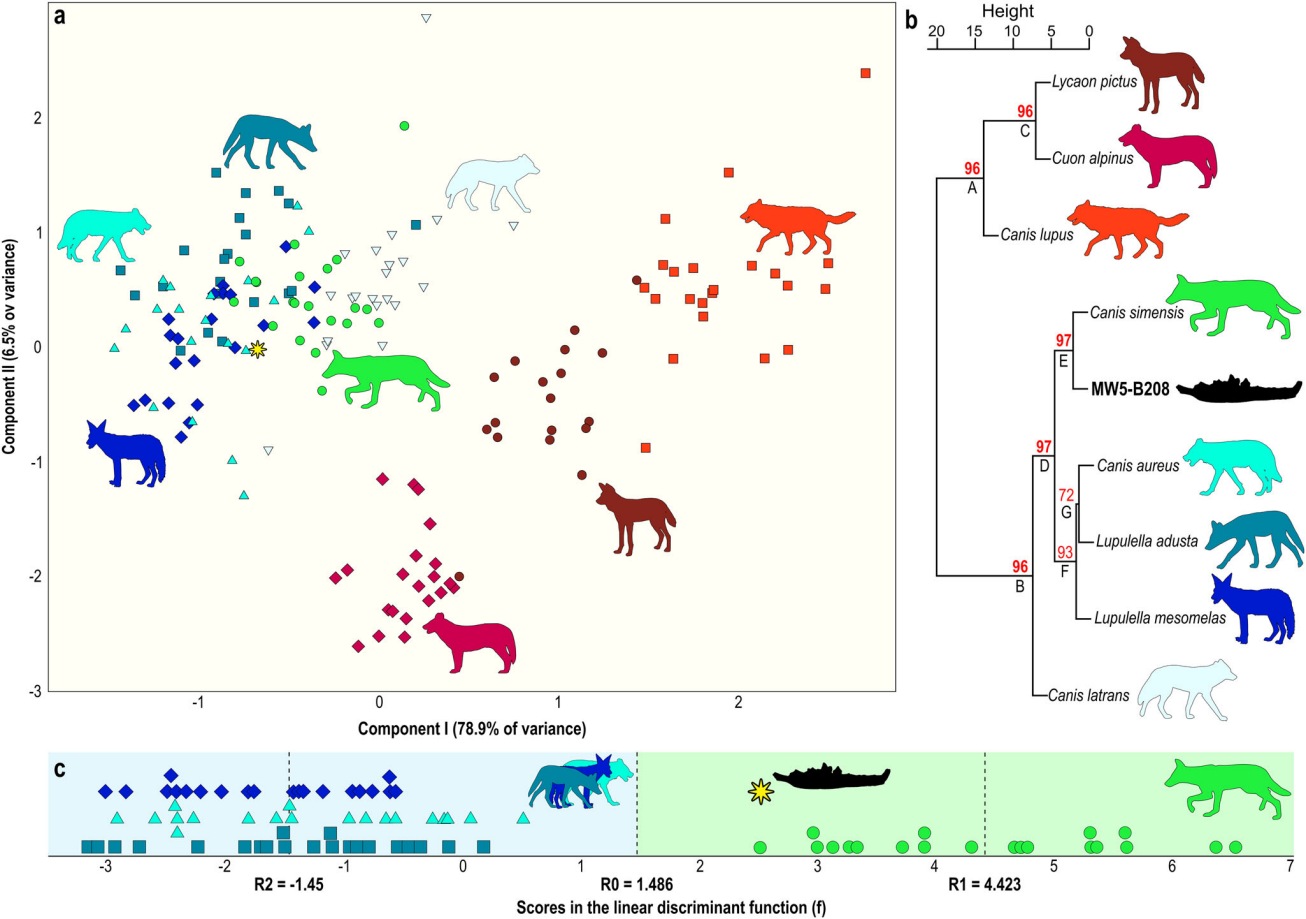
Morphometric comparison analyses of the mandible MW5-B208 from Melka Wakena as opposed to extant canids. **a** Principal component analysis on dentognathic variables. The graph shows a clear separation between the mesocarnivorous canids (on the left) and the three species of pack hunting hypercarnivores (on the right). Yellow star represents the position of MW5-B208 from Melka Wakena. **b** Bootstrap cluster analyses on PCA scores, confirming the similarity of MW5-B208 to extant *Canis simensis*. Numbers represent the percentage *p*-values supporting the node: values b above 95% are reported in bold red. **c** Linear discriminant function between the three species of jackals and the Ethiopian wolf. This function reclassifies correctly 100% of the specimens of the living canid species and assigns the Melka Wakena fossil jaw to the group of *C. simensis*. However, the score of the specimen in the discriminant function is closer to the limit between the groups compared (R0) than to the group centroid of Ethiopian wolves (R1), which suggests that the fossil jaw does not show the fully evolved condition of a mandible of *C. simensis*. Silhouettes of canids made by authors (S.B.L.).

Morphometrically, MW5-B208 takes a position on the morphospace between the golden jackal and the Ethiopian wolf (Fig. 4a). But the principal components analysis (PCA) distribution pattern (cluster) does not help in clarifying the morphological affinities of the fossil. As a result, a linear discriminant analysis between the three living species of jackals and the Ethiopian wolf based on six variables (i.e., Lp4, Bp4, Lm1trig, Bm1tal, Lcm2, and JBp3p4) was employed (Fig. 4c). The analysis reclassifies correctly all the specimens in their respective groups after cross-validations using the leave-one-out method (see Methods subsection “Comparative sample, programs, and software used for the morphological and morphometric analyses”). This function classifies unequivocally the Melka Wakena fossil as a jaw of *C. simensis* with a probability of *p* = 0.997. The results of the bootstrapping cluster analysis (BCA) confirm the general pattern of the PCA, with the hypercarnivorous canids (*C. lupus*, *C. alpinus*, and *L. pictus*) on one side of the plot, whereas the species of the genus *Lupulella* (i.e., *L. adusta*, *L. mesomelas*), *C. aureus*, *C. latrans*, *C. simensis* and the *Canis* from Melka Wakena are grouped in the negative side of the first principal component. Indeed, the BCA results identify two well-separated clusters composed of the same species (Fig. 4b). Within the larger cluster, the hemimandible MW5-B208 from Melka Wakena is grouped with the extant *C. simensis*. The supporting value of the grouping is the highest of the analysis (percentage *p*-value = 97%), thus testifying to the strong similarity in the values of the two species. The results of the BCA fully confirm the classification of the discriminant function in clustering the fossil specimen close to the extant Ethiopian wolf.

### Biogeography and phylogenetic relationships with other canids

Recent phylogenetic studies have demonstrated that *C. simensis* is closely related to species of the crown-group *Canis*, related to the extant gray wolf (*C. lupus*)^14–17^ (Supp. Note 3, Supp. Figure 3). This might hint at the ancestry of extant Ethiopian wolf populations, since true *Canis* species are well-represented in the Eurasian fossil record from 2.5 Ma and are commonly related to living canids, for example the lineage *Canis borjgali–C. orcensis–C. mosbachensis* to *C. lupus*^18–21^ (Sup. Inf.), supporting new interpretations^17^ and contrasting with previous estimates based on mtDNA of a late Middle–Late Pleistocene differentiation of *C. simensis* from a wolf-like ancestor at around 100 ka^12^. An alternative hypothesis based on molecular data—that *C. simensis*’ time of divergence from other crown-group *Canis* species was around −2.5 Ma—has thus far lacked empirical evidence^17^ (Sup. Inf.). The discovery of MW5-B208 at the time span of 1.6–1.4 Ma, with all the dentognathic peculiarities of its extant relatives and very few differences, constitutes the first empirical support for the genetic interpretations of *C. simensis* phylogenetic relationship with the crown-group *Canis*.

The Early Pleistocene dispersal of *C. simensis*’ ancestors from Eurasia to Africa appears to have been coeval with that of the ancestors of the hypercarnivorous *Lycaon pictus (*African hunting dog), which arrived to Africa later than 1.8 Ma^22,23^. At the same time, 2.0–1.8 Ma, yet in the opposite direction, hominins dispersed into Eurasia, as indicated by the record at the site of Dmanisi^24,25^. In the absence of other earlier evidences, this hypothesis fits with the new date of the fossil record of *C. simensis*, 1.6–1.4 Ma.

### Modeling the *Canis simensis*’ niche for past and future projections

**Fig. 5:**
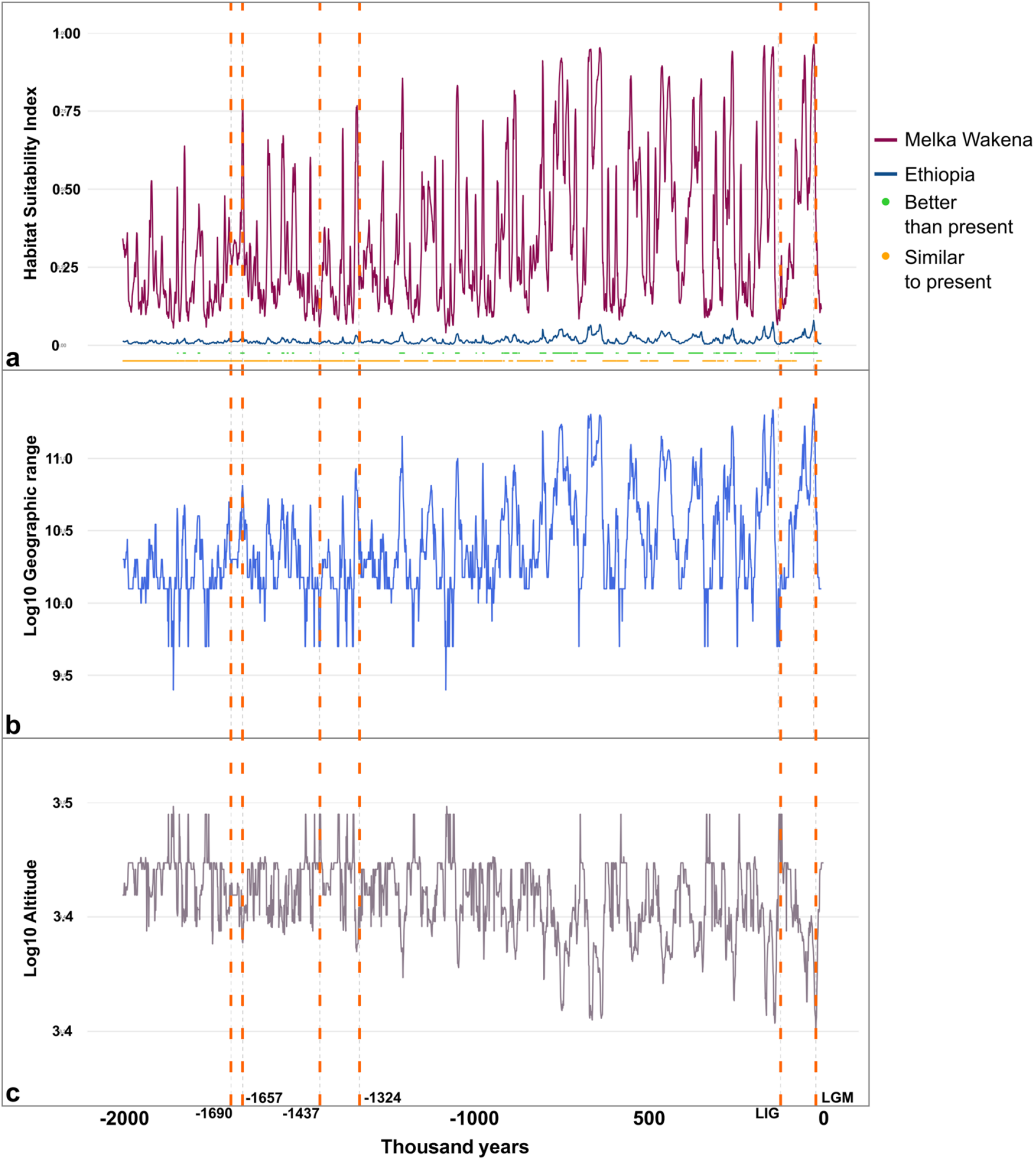
Temporal trend of ecological and environmental parameters of *C. simensis* in Ethiopia and Melka Wakena. **a** Temporal trend of Habitat Suitability Index (HSI) values computed for the last 2 Ma at the site of Melka Wakena (violet line), as mean value in the whole Ethiopia (blue line). At the base of this panel (**a**) a sequence of green points indicates the temporal intervals with environmental conditions in Ethiopia better than the present time interval; below, the sequence of orange points indicates temporal intervals with conditions similar to the current climate in Ethiopia. In some cases, these two different sequences of green and orange points apparently overlap in time, thus generating time bins that seem to include both better and similar conditions to present Ethiopia. This is only a graphical issue due to limit of minimum pixel size in the image. In order to make present and past time bins comparable, current climatic conditions were determined by using the Raia et al.^55^ raster layer for the present. **b** Temporal trend of the species’ geographic range’s Log10 surface area. For species’ geographic range computation, see “Methods” section. **c** Temporal trend of the Log10 mean altitude sampled by the species in the reconstructed geographic range. Orange vertical dashed lines indicate time intervals important to the fossil localitity of Melka Wakena: 1690 ka, the maximum estimated radiometric age of the site; 1657 ka, age with the highest HSI value reconstructed for the site in the estimated age range; 1437 ka, age with the lowest HSI value reconstructed for the site in the estimated age range; 1324 ka, the minimum estimated radiometric age of the site; LIG, Last Interglacial (~123 ka); LGM, Last Glacial Maximum (~22 ka).

**Fig. 6:**
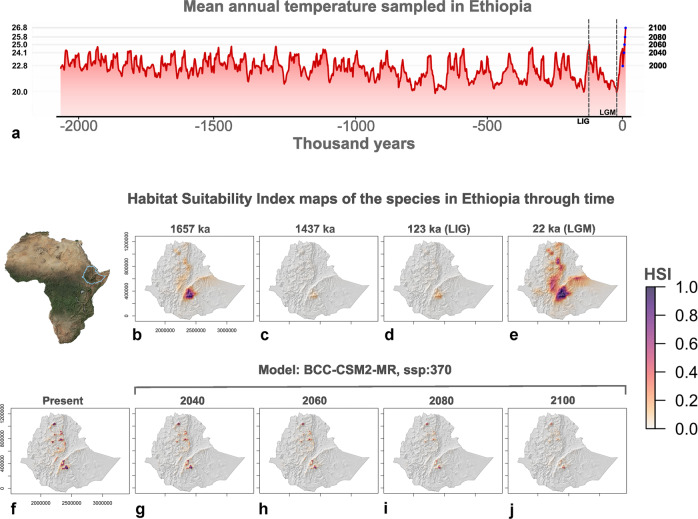
Temporal trend of climate in Ethiopia and of habitat suitable to *C. simensis*. **a** Reconstructed Annual Mean Temperature (BIO 1) averaged for the whole Ethiopia territory during the last 2 Ma, based on Raia et al.^55^, the present and future forecasts in 2040, 2060, 2080, and 2100 (based on the model BCC-CSM2-MR with ssp: 370, see Supp. Note 5 for further information). Before averaging temperature values, present and future temperature raster were upscaled to a resolution of 50 × 50 km, as for the past bioclimatic rasters. Blue dots indicate the temperature values for the present, for 2040, 2060, 2080, and 2100 (see Supp. Note 5 for other future models and scenarios). The Last Interglacial (LIG) and the Last Glacial Maximum (LGM) are also indicated by the dashed-gray vertical lines at 123 and 22 ka; **b**–**e** maps of the reconstructed Ethiopian wolf’s Habitat Suitability Index in Ethiopia for 1657 Ka (**b**), 1437 Ka (**c**), 123 ka (LIG, **d**), and for 22 ka (LGM, **e**); and **f**–**j** Habitat Suitability Index map of the present (**f**) and forecasted maps for the 2040 (**g**), 2060 (**h**), 2080 (**i**), and 2100 (**j**) according to the CMIP6 model BCC-CSM2-MR with the shared socio-economic pathway 370 (see “Methods” for further information about CMIP6 models). Map of Africa modified by the authors (F.C.) from https://neo.gsfc.nasa.gov/view.php?datasetId=BlueMarbleNG, credits to NEO (Nasa Earth Observations, https://neo.gsfc.nasa.gov/). Maps showing the *C. simensis* spatial distribution through time generated modified by authors (F.C.) by applying Maxent models to the bioclimatic variables’ rasters.

The current regional topography of the Southeastern Ethiopian Highlands was largely shaped by late Pliocene tectonic uplift (ca. 4.5 Ma), after which the landscape stabilized to its current topographic configuration^26^. By modeling the species’ bioclimatic requirements in the present, we were able to project its ecological niche onto simulated past and future environmental conditions, creating Habitat Suitability Index (HSI) maps that identify the favorable bioclimatic conditions in Ethiopia through time for *C. simensis* (Figs. 5 and 6). Given the species behavior and ecological needs, this allows us to estimate potential fluctuations in its geographic range. The reliability of these projections was guaranteed by checking for models’ transferability (Supp. Table 2 and Supp. Note 5).

Nowadays, *C. simensis* is adapted to live in the alpine grassland environments of the highest Ethiopian mountains, between 3000 and 4500 m asl^4^ and with cold climates during most of the year^27^. The model predictions used in this study (see Bioclimatic variables in “Methods”) are consistent with the empirical information and indicate that the species occupies territories with an annual mean temperature ranging from ~6 °C to 13 °C, and an annual temperature excursion (i.e., highest temperature of the warmest month minus the lowest temperature of the coldest month) ranging from 15 °C to ~22 °C. Also, the annual mean precipitation range is wide (from 890 to 1639 mm) with more average rainfall occurring during the warmest months (Supp. Note 5). Our models are consistent with the Early Pleistocene palynological record of the Melka Kunture archaeological series^28^, also in the Ethiopian highlands (Supp. Note 1, Supp. Figure 1), which indicates that similar favorable environmental conditions for the survival of the Ethiopian wolf already existed when its ancestor arrived in Ethiopia (Figs. 5a and 6a, Supp. Note 5).

Within the radio-isotopic age interval for the Melka Wakena site-complex, our results suggest that the time interval with the highest percentage of highly suitable territories for *C. simensis* was around 1657 ka (Fig. 5a), when favorable conditions occurred throughout the Ethiopian highlands (Fig. 6b). Conversely, the model suggests a drop in habitat suitability at around 1437 ka (Fig. 5a), leading to a highly reduced potential geographic range of the Ethiopian wolf (Fig. 5c).

According to our models, changes in the species’ potential geographic distribution were less dynamic up to 1 Ma, whereas it contracted and expanded substantially and repeatedly with the onset of 100-ka glacial cycles (Brown-Forsythe Test^29^: 422.1953, *p* ≪ 0.001, Fig. 4b). Our tests confirmed a significant and negative relationship between the species’ potential geographic range and both the mean temperatures (ARIMA regression: slope = −0.097, *p* ≪ 0.001) and altitude sampled by the species (ARIMA regression: slope = −4.160, *p* ≪ 0.001) during the last 2 Ma (Supp. Note 5, and Supp. Figures 4 and 5). During periods of warmer climate, its potential geographic range was drastically reduced, forcing the species to move to higher elevations, and during periods of cooler climate its potential geographic range expanded into lower topographic elevations, favoring the connection of previously spatially fragmented isolated populations (Fig. 5c). Interglacial periods, such as the Eemian (from about 130 to 115 ka) in our models, represent times of maximum deviation from the estimated favorable climatic conditions for the species (Fig. 5a) and, in turn, of maximum reduction and population fragmentation within its potential geographic range (Fig. 6d). Thus, these periods of warm climate may have driven the populations close to a bottleneck, and possibly led some of them to extinction^30^. However, the general cooling of the climate during the Middle and Late Pleistocene increased the potential habitat area of *C. simensis* populations and may have allowed them to move to lower elevations, connecting and exchanging genes with adjacent populations. The overall population size would recover when the different populations connected physically. In summary, we show that the Ethiopian wolf’s favorable conditions and geographic range as predicted from our hind-sight models, expanded during cold phases of the Pleistocene (Supp. Note 5); the Last Glacial Maximum (LGM about 26–18 ka) could have been one of the most favorable phases (Figs. 5b and 6e). These analyses show that *C. simensis* was always an endemic species, with its maximum potential geographic range restricted to the Ethiopian highlands (Fig. 6b–e), represented by small populations, mostly fragmented during the warmer phases of the Quaternary. This is coherent with the hypothesis of the Long-term Small Populations Size and small genetic diversity of *C. simensis*, with a succession of several bottlenecks during its evolutionary history^31^.

By modeling its favorable climatic conditions, we infer suitable territories at present and forecast the fate of *C. simensis* in the Ethiopian highlands over the next 80 years. Our models imply that present and future global warming constitutes a detrimental factor for the Ethiopian wolf’s survival. In the past 2 Ma, the Ethiopian highlands never experienced the temperature increase forecasted for the future (Fig. 5a). This increase implies a significant shrinkage of the Ethiopian wolf’s habitat, which may drastically reduce its geographic range and bring populations to the brink of a bottleneck. There is also evidence that the current global climate change has already induced phenological shifts in top predators, with drastic consequences for their reproductive success^32^. Current climatic conditions already determine very restricted contiguous areas suitable to the species (Fig. 6f and Supp. Table 3). For the future, even optimistic projections, taking into account adequate climate policies (Models: CNRM-CM6-1 and CNRM-ESM2-1, ssp: 126, with mean global temperature increase lower than 2 °C in 2100, Supp. Note 5), indicate that the most favorable habitats will be reduced up to about 65% of its current extent by 2080 (Supp. Note 5) and that they will totally disappear afterward (Supp. Note 5, Supp. Tab. 4). Indeed, more pessimistic projections of faster increases of temperature and CO_2_ emissions predict a habitat loss of ~95% (Model: BCC-CSM2-MR, ssp: 585, year: 2100, Supp. Note 5) and up to 100% (Model: CNRM-CM6-1, ssp: 370 and 585, year: 2080; Model: CNRM-ESM2-1, ssp: 585, year: 2100) of the most suitable territories (see Supp. Note 5, Supp. Table 4, for intermediate scenarios). By considering an intermediate-pessimistic increase of temperature (BCC-CSM2-MR, ssp: 370), we forecasted a reduction of the species’ most suitable conditions of about 28% by 2040 (Fig. 6g), ~63% by 2060 (Fig. 6h), ~74% by 2080 (Fig. 6i), and ~87% by 2100 (Fig. 6j). In general, the preferred conditions for the Ethiopian wolf will be confined to the mountain tops but will be highly reduced geographically (OLS regression, Supp. Note 5) and fragmented into many patches with poor physical connections (MIROC6 model, GLM regression, Supp. Note 5 and Supp. Table 5), although different future climatic projections may impact differently on the species’ habitat (Supp. Note 5 and Supp. Table 6).

## Discussion

Our comparative analysis of the extant specimens of African canids shows that MW5-B208 belongs to a fossil form of the *Canis simensis* lineage (Figs. 3 and 4; Supp. Tab. 1, Supp. Note 4). Both anatomical and morphometric analyses confirmed similarity between the specimen from Melka Wakena and the extant Ethiopian wolf. It is worth noting that the fossil takes a discriminant score that situates it in the lower limit of the range of values shown by Ethiopian wolves in the discriminant function. This may be tentatively interpreted as indicating that while MW5-B208 shows the metric features of the lower dentition and jaw of a specimen of *C. simensis*, it does not represent the fully evolved condition of this species. Given that the Early Pleistocene altitude of Melka Wakena was similar to the current situation (~2300 m above sea level)^26^, the specimen MW5-B208 constitutes the first-ever evidence that *C. simensis* was present on the Ethiopian highlands from at least 1.6–1.4 Ma.

The geographic position of Melka Wakena coincides with the territory where our model identifies some of the highest values of the HSI (i.e., indicating highly favorable conditions for the species) throughout Ethiopia over the last 2 Ma (these values were higher than 99% of the area of the whole region). Since habitat suitability is typically correlated with population abundance^33^, the likelihood was high that the species’ fossilized remains would have been found in this area^34^.

Our study renders the absence (until the current discovery) of *C. simensis* from the fossil record a likely result of the scarcity of documented archaeological and paleontological sites on the Ethiopian highlands. The bulk of the Quaternary fossil record in eastern Africa derives from the rich paleoanthropological and paleontological sites in the rift valley, where *C. simensis* most probably would not be present, given its adaptation for highland environments. The discovery of MW5-B208 thus underscores the importance of paleoanthropological research outside the East African Rift System.

The Ethiopian wolf is one of the most iconic species on the African continent. While our study is not aimed to outline conservation strategies, it does underscore the urgent need for such programs in order to safeguard the remaining habitats of this ecologically specialized, highly endangered species and to prevent its reproductive depression. Since its adaptation to the Afroalpine environments, at least 1.6–1.4 Ma ago, *C. simensis* most probably has been at the brink of extinction several times due to climate changes, yet it has always recovered. This species is a true biological legacy that belongs to the world’s nature heritage. It is an ethical as well as a scientific duty to preserve and draw the Ethiopian wolf from the brink of extinction and turn it into a persistent animal.

The discovery of the ~1.6–1.4 Ma specimen MW5-B208, coupled with the genetic data that suggest its speciation at an even earlier date, suggests that populations of the Ethiopian wolf likely survived unfavorable environments throughout a number of climatic cycles since their arrival to the Ethiopian highlands. Additional information, with the application of new methods to reconstruct the geographic distributions of past species^35^, is needed to understand how *C. simensis* overcame past periods of habitat loss, which might help in building conservation programs for this iconic animal.

## Methods

### Comparative sample, programs, and software used for the morphological and morphometric analyses

The present study describes and analyses a canid mandible (MW5-B208) recovered from the Early Pleistocene site Melka Wakena (Ethiopia), comparing it taxonomically to other African Plio-Pleistocene medium-sized canids and to selected extant species of the genera *Lupulella* and *Canis*. The subject fossil is housed at the Ethiopian Heritage Authority (EHA) in Addis Ababa. The Villafranchian and Epivillafranchian canids from Africa housed at Musée National d’Histoire Naturelle (MNHN, Paris) as well others from the literature were utilized as comparative fossil material. This fossil comparative sample includes specimens of *Lupulella paralius* Geraads, 2011 from Ahl al Oughlam, Morocco^36^; *Lupulella mohibi* Geraads, 2011 from Grotte de Rhinoceros, Morocco^36^; *Lupulella* sp. from Koobi Fora, Upper Burgi-KBS Members^37^; *Eucyon kuta* Werdelin et al., 2015 from Aralee Issie, Ethiopia^38^ and *Canis brevirostris* Ewer, 1956 from Sterkfontein Member 4, South Africa^39^ (digital data obtained thanks to the courtesy of Dr. M. Tawane and Dr. J. Adams from Morphosource^40^). Extant specimens housed at the American Museum of Natural History (AMNH, New York), “La Specola” Museo di Zoologia di Firenze (MZUF, Florence), Naturhistorisches Museum Basel (NMB, Basel), Musée National d’Histoire Naturelle (MNHN, Paris), and EHA (Addis Ababa) were also used for morphological and metrical comparisons. We examined specimens of *Lupulella adusta* (Sundevall, 1847), *Lupulella mesomelas* (Schreber, 1775), *Canis lupaster* Hemprich and Ehrenberg, 1832, and *Canis simensis* Rüppell, 1840. Moreover, a wide dataset of craniodental measurements of modern canids, taken by Prof. Blaire Van Valkenburgh, was used also in some statistical comparisons, including a discriminant analysis between omnivorous (i.e., meso- and hypo-carnivorous) and hypercarnivorous canids, in order to reach paleoecological inferences for the Melka Wakena canid. Cranial and dental measurements were taken with a digital caliper to the nearest 0.1 mm^41^. Photographs of the specimens were taken with a camera Canon EOS 200D and with a Canon 60D and subsequently processed with Photoshop CC 2017 (release 2017.0.1). Inkscape ver 0.92.4 was used to compose and finalize the figures. Three-dimensional data of specimen MW5-B208 were acquired using a handheld high-resolution 3D scanner EinScan Pro HD (www.einscan.com) located at EHA. Scanning was carried out by one of the authors (T.G.) with the help of EHA curator Sahlesellasie Melaku. This hand-held scanner is compact and highly suitable for in situ scans of small to medium-sized objects. Its field-of-view (FOV) ranges between a maximum of FOV = 31.0 × 24.0 cm and a minimum of 20.9 × 16.0 cm with an adjustable scan speed between 10 to 30 frame per seconds. Its accuracy is around 0.1–0.3 mm depending on the chosen preset (respectively “scan” or “rapid scan”), making it suitable for small objects. In order to obtain the 3D model of the specimen, raw data were acquired with multiple passes of the scanner on the mandible, which was oriented in different views and positions to allow a full coverage of the surface of the specimen. Acquisition and subsequent elaboration of the data were performed in the native software EinScan-Pro series ver. 3.1.0.2. This program enabled cleaning, alignment, and refinement of the data prior to the creation of the 3D mesh of the specimen. The rapidity and quality of the scanner allowed obtaining a valuable digital reproduction of the fossil in a short time.

Morphometric analyses^42–47^ of the metric affinities of the Melka Wakena specimen were carried out using nineteen metric variables, measured on the teeth and on the portions of the mandibular ramus preserved in the fossil jaw. The latter include: the anteroposterior length and buccolingual breadth of the lower canine (Lc and Bc, respectively) measured at the base of the tooth crown, the length and breadth of the fourth premolar (Lp4 and Bp4, respectively), the length of the trigonid blade in the lower carnassial (Lm1trig), the maximum breadth of the carnassial (Bm1), the length and breadth of the talonid basin in the carnassial (Lm1tal and Bm1tal, respectively), the length and breadth of the second molar (Lm2 and Bm2, respectively), the length between the anterior tip of the canine and the posterior border of the second molar (Lcm2), the height and breadth of the mandible at the posterior border of the canine (JDc and JBc, respectively), the height and breadth of the mandible at the contact between the third and fourth premolars (JDp3p4 and JBp3p4, respectively), the height and breadth of the mandible at the contact between the fourth premolar and the carnassial (JDp4m1 and JBp4m1, respectively), and the height and breadth of the mandible at the contact between the carnassial and the second molar (JDm1m2 and JBm1m2, respectively). Abbreviations are also reported in a separate section.

A principal components analysis (PCA) was performed after logarithmic transformation of these metric variables on a sample of seven species of living canids, which includes four mesocarnivorous species [the Ethiopian wolf (*Canis simensis*, *n* = 20), the golden jackal (*C. aureus*, *n* = 19), the side-striped jackal (*Lupulella adusta*, *n* = 21), the black-backed jackal (*Lup. mesomelas*, *n* = 21) and the coyote (*C. latrans*, *n* = 21)]. The sample also includes three hypercarnivorous, pack hunting species [the gray wolf (*C. lupus*, *n* = 22), the African painted dog (*Lycaon pictus*, *n* = 19), and the dhole (*Cuon alpinus*, *n* = 21)]. Two other tests were performed to assess the similarity of MW5-B208 to the other fossil species in our sample. Firstly, we performed a linear discriminant analysis between the three species of jackals (*n* = 61) and the Ethiopian wolf (*n* = 20), using the Wilks lambda criterium for selection of variables (*λ*_wilks_ = 0.132, *c*^2^ = 153.912, *p* < 0.0001). The linear function obtained incorporates six variables (Φ = −0.577Lp4 + 1.545Bp4 + 0.277Lm1trig − 0.839Bm1tal + 0.256Lcm2 − 0.623JBp3p4 − 14.966) attempting to reclassify each specimen via cross-validations with the leave-one-out method. A second classification algorithm performed on the resulting data of the PCA is a permutation cluster analysis: bootstrapping cluster analysis (hereafter BCA) is a partitioning method, helpful to test the robustness of the clustering results. We briefly outline the way the methodology works: a preliminary clustering of observations is performed via an UPGMA algorithm (unweighted pair group method with arithmetic mean)^48^ to be used as a reference cluster in which the fossil species are grouped according to similarity in their values. Subsequently, the algorithm assigns a probability value to each partitioning level in the reference clustering. Then a sample of the original data (i.e., rotated a) is randomly chosen and used to feed a new UPGMA cluster analysis, which yields a new cluster. The comparison between the reference and the sample clusters yields a similarity index (G) that ranges between 0 (if the two clusters are totally different) and 1 (if the clusters generated with the original and sampled data coincides). The G* is then compared with the expected similarity value between reference and sample-based clustering (G°) under the null hypothesis that the sampled dataset is a truly random sample of the original data. The resampling the original dataset was reiterated 1000 times. In the end, if the probability that G* is higher or equal to G° is higher than the significant level (P(G° ≤ G*); *α* = 0.05), the partitioning levels of the reference cluster analysis are sharp (for a detailed explanation of the algorithm, see DePatta Pillar^49^).

*Abbreviations for the morphometric analyses*: B: maximum buccolingual breadth of the tooth measured at the base of the tooth crown; JBc: breadth of the mandible at the posterior border of the canine; JDc: the height of the mandible at the posterior border of the canine; JBp3p4: breadth of the mandible measured at the interalveolar space between the third and fourth premolars; JDp3p4: the height of the mandible measured at the interalveolar space between third and fourth premolars; JBp4m1: breadth of the mandible measured at the interalveolar space between the fourth premolar and the carnassial; JDp4m1: the height of the mandible measured at the interalveolar space between the fourth premolar and the carnassial; JBm1m2: breadth of the mandible measured at the interalveolar space between the carnassial and the second molar; JDm1m2: the height of the mandible measured at the interalveolar space between the carnassial and the second molar; L: maximum mesiodistal length of the tooth measured at the base of the tooth crown; Lcm2: the length between the anterior tip of the canine and the posterior border of the second molar; m1tal: trigonid portion of the lower carnassial; m1trig: trigonid portion of the lower carnassial.

### Modeling the bioclimatic niche through time

The reconstructions of past and future spatial distribution of the Ethiopian wolf and related analyses require mathematical modeling of the species’ bioclimatic niche followed by the projection of this niche onto the geographic space. This procedure is known as Species Distribution Modeling (SDM)^50^ and is widely employed for both living^51^ and fossil species^35,52^. The output of this projection is a map of the species’ Habitat Suitability Index (HSI map), which is the reconstruction of the territories suitable to the species’ habitat in the geographic space and that can be used to derive the species’ geographic range and metrics of the landscape connectivity structure. The procedures used to model the bioclimatic niche of *C. simensis*, to forecast its geographic range expansion or contraction in the next 80 years and to project these conditions to the past 2 Ma are described in the following section. We present future projections for *C. simensis*’ under different scenarios of climate change. Using models of past temporal intervals, we explored whether the habitat of Ethiopian wolf already existed before and during the time span suggested for human occupations at the Melka Wakena site-complex and to study the species’ temporal evolution in the area of present-day Ethiopia; for the future, we aimed at estimating the degree of habitat loss caused by global warming. Furthermore, the past and future habitat reconstructions were built upon different environmental variables and at different spatio-temporal resolutions. This means that past and future *C. simensis*’ habitat reconstructions cannot be compared to each other although being accurately reconstructed for their scopes. We named the models for projecting the bioclimatic niche onto past temporal intervals the “Hindcasting Model” (HM), whereas the models for projections onto the future the “Forecasting Model” (FM).

### Species’ occurrences

Information about the present occurrences of C*. simensis* is unsystematic and sporadic. GBIF public repository (www.gbif.org) registered 96 occurrences, but the error of the geographical positions was too high to allow an accurate spatial modeling. Instead, we used the geographic range polygons provided by the IUCN (International Union for the Conservation of Nature, https://www.iucnredlist.org/) for *C. simensis* as a framework for simulating species occurrences. According to the most recent available estimates, there are 197 mature reproductive individuals within the total census population^4^; therefore, we simulated a total of 200 random points falling inside the polygons of the species’ geographic range. This procedure is widely used for modeling species distribution in the absence of accurate true presence data^53^. Because the species is narrowly distributed and the IUCN polygons are drawn from empirical data and the species actual occurrences and movements, simulating presence points inside the polygons approximates the populations’ general distribution. In our simulation, only one presence point could occur in any single grid cell of the bioclimatic variables raster used to model the specie’s niche. This procedure is helpful to reduce overfitting during the modeling process. Since we used bioclimatic variables with different spatial resolutions for the Hindcasting and Forecasting models we used (see below), we produced two sets of simulated occurrences, one each for the past and for the future models, with spatial grids of 4 × 4 and 1 × 1 km cell resolution, respectively.

### Bioclimatic variables

We used different sets of bioclimatic variables to first model the species’ bioclimatic niche on, and then to project it onto future and past time intervals. The Forecasting models and the Hindcasting models derive from the same set of current bioclimatic conditions, differing in the number of the variables considered in the models. Following this, the Forecasting and Hindcasting models were projected onto different temporal intervals, in the next 80 years and within the past 2 Ma, respectively.

For calibrating the Hindcasting models, we used four of the nineteen bioclimatic variables of the present time interval as provided by www.worldclim.org^54^, with a spatial resolution of 4 × 4 km grid cell combined with a Net Primary Productivity (NPP) layer (see the next paragraph for details). For past projections of the species’ niche models, we used the improved version^55^ of the PLASIM-GENIE paleoclimatic emulator data^56^ including four bioclimatic and NPP layers at a spatial resolution of 50 × 50 km grid cell and a temporal step of 1000 years. The PLASIM-GENIE bioclimatic dataset spans the last 5 Ma, including the reconstruction of the current climatic conditions at the same spatial resolution, a fact that makes past habitat reconstructions comparable with present habitat estimations (see below). We used these climate projections to reconstruct the species’ spatial distribution from the present to 2 Ma.

For calibrating the Forecasting models for projection onto the future, we used all the nineteen bioclimatic variables at a spatial resolution of 1 km side grid cell, as provided at http://www.worldclim.org, in order to reconstruct the most detailed current realized species’ niche. We used the bioclimatic variables provided by http://www.worldclim.org at a 4 × 4 km grid cell resolution for the four future time intervals: 2021 to 2040, from 2041 to 2060, from 2061 to 2080, and from 2081 to 2100. Future climates are simulated by different research institutions that are coordinated by the Coupled Model Intercomparison Projects, now at its stage 6 (CMIP6). Each institution uses a specific emulator to predict future climate. Nevertheless, all these future projections are modeled by taking into account different shared socio-economic pathways scenarios (ssp). These scenarios hypothesize future socio-economic projections and political environments which would drive to different CO_2_ emissions’ rates in future years^57^. The CO_2_ emission rate is a parameter used to forecast the future temperature variation. Each institution’s emulator yields a climate forecast that has a specific response to the above-mentioned CO_2_ emission scenarios. This means that each emulator produces a climate forecast by considering a specific “climate sensitivity”, i.e., the rate of temperature increase with the increase of CO_2_ in the atmosphere. The CMIP6 project produced future climates with a sensitivity ranging from 1.8 and 5.6. The IPCC AR5 report^58^ considered as plausible a range from 1.5 and 4.5. For our future projections of the Ethiopian wolf climatic niche, we consider four out of all the available CMIP6 emulators: the MIROC6 with a sensitivity of 2.6; the BCC-CSM2-MR emulator with a climate sensitivity of 3; CNRM-CM6-1 with a sensitivity of 4.3; and CNRM-ESM2-1 with a more extreme sensitivity of 4.8. For each CMIP6 model, we considered the four most important ssps with an increasing CO_2_ emission rate through time (for further details, Meinshausen et al.^57^): ssp 126, ssp 245, ssp 370, and ssp 585. Present, past and future bioclimatic variable rasters were projected into a Lambert Azimuthal Equal Area projection with projection centre at the African continent before performing Species Distribution Models (SDM). We used all the bioclimatic data as provided at the downloading date, 13 June 2021.

### Variables’ pre-processing

The model used to project the Ethiopian wolf climatic niche onto past time intervals could not use all the present bioclimatic variables since the raster dataset^54,55^ includes only four out of the nineteen bioclimatic variables available for the present time interval, with an additional NPP layer. We included in the Hindcasting Models the following variables: BIO 10 (Mean Temperature of Warmest Quarter), BIO 11 (Mean Temperature of Coldest Quarter), BIO 18 (Precipitation of Warmest Quarter), BIO 19 (Precipitation of Coldest Quarter) and NPP. To reduce the effect of multicollinearity between these variables, we performed a Principal Component Analysis and considered the related five PC scores for all the modeling processes. Santini et al.^59^ recommended to check for exportability through time of the models, since models calibrated with present variables might produce extrapolations when projecting onto climatic conditions of different time intervals. We performed a Multivariate Environmental Similarity Surface analysis (MESS)^60^ on all the variables of all the time intervals to detect those raster’s pixels with values’ combinations not included in the present climate. Before performing the MESS analyses, we first turned past variables into PCA scores by applying on them the PCA model calibrated with the variables of the present time interval. After the MESS analyses, we ruled out a specific past bioclimatic variable if, considering all the temporal intervals, its mean proportion of the out-of-the-range values in the total Africa raster would have exceeded 20%. We anticipate here that none of the five PCA raster variables would be ruled out from the modeling procedures.

For future projections of the climate, we performed the same MESS analyses but, because future climate projections shared the same bioclimatic variables with current conditions, we did not perform the PCA. Instead, we reduced the possible effects of multicollinearity by removing from the set of present bioclimatic variables those with a Variance Inflation Factor higher than 3^61^. After this procedure, eight out of nineteen bioclimatic variables proved useful for the modeling process. For the future projections, too, none of the remaining bioclimatic variables showed critical issues of extrapolation after the MESS analyses.

We performed the described analyses and pre-processing procedures with all the CMIP6 future projections and ssp scenarios.

### The hindcasting and forecasting modeling procedures

As mentioned above, Hindcasting Models (HMs) are trained for past niche projections, whereas the Forecasting Models (FMs) were employed for projecting species’ niche onto future ones. Both sets of models were calibrated by combining the simulated species’ occurrences, as described above, with the climate of the present. For the calibration of both Hindcasting and Forecasting models we employed the procedure called “ensemble of small models”^62^. Rather than creating a single model with all the available variables, this procedure combines a number of models built upon different combinations of a subset of the predictor variables. The authors suggest to average the predictions of all the models by weighting them according to their accuracy. This procedure is a strategy to model the bioclimatic niche of small-ranged, endemic species, like *C. simensis*, in order to avoid model overfitting, an issue that may result in an underestimation of the species’ niche breadth. For the Hindcasting models, we only used five bioclimatic variables of past intervals. While employing PC axes would have reduced variable’ collinearity, a small number of predictors is not advisable because it could determine model’s overfitting. Hence, the strategy of Breiner et al.^62^ was prioritized. We trained five small models, each built upon a combination of four out of the five available PC variables. For forecasting, we built 28 models, each using a combination of six out of the eight bioclimatic variables.

### The Maxent model

The SDM we used to predict the species’ habitat suitability was the Maxent algorithm^63^. Maxent belongs the family of presence-only methods in which only species occurrences are required other than the environmental variables. Being currently one of the most popular algorithms for presence-only datasets, Maxent relies on the assumption that the observed species occurrence locations represent a random sample of its true geographic distribution. The aim of the algorithm is to find the true species probability distribution in the study area, which is initially unknown. Further, since many theoretical assumptions are violated in the real world, the Maxent output is better interpreted as measure of the species habitat suitability in the study area than its occurrence probability (for a broad discussion about the Maxent output interpretation, see Merow et al.^64^ and Elith et al.^65^). In Maxent algorithm the true (unknown) species’ occurrence probability in the study area (the landscape, *sensu* Elith et al.^65^ is a function of the Probability Density Function (PDF) of the environmental variables sampled at the presence locations and of a prior hypothesis. Maxent aims to find an approximation of this unknown true probability distribution. The theoretical framework of this algorithm is given by the maximum entropy principle^66^, according to which the best approach to approximate the true PDF of an event is to find the distribution with the maximum entropy, while still satisfying any known constraint^63^. In other words, to assess the possible state of an event at an unknown location, we have to maximize the number of possible choices of the state of the event. Maximizing the number of choices means maximizing the entropy of the system^67^. The Maxent algorithm starts from the most maximized and unconditioned PDF, which is represented by the uniform distribution^63,64^, and applies some constraints determined by the characteristics of the PDFs of the variables at presence points. In thermodynamic terms, finding the PDF with the maximum entropy equals to expanding a gas into a larger volume after providing some energy to its molecules (thus increasing the molecules’ chaotic movements) but controlling for the volume’s shape. With a uniform distribution a species has the same chance to be present in any location of the study area. In ecological terms, this means that the probability of the species to be present in a location is a function of the relative abundance in the landscape of the environmental conditions found there^64^. Hence, the probability density function of environmental variable values in the study area represents our prior hypothesis about the distribution with maximum entropy. If Z is the set of predictor variables geographically distributed over the study, this prior is defined as *Q(z)* and the Maxent algorithm can be explained by calculating the probability of finding the variables’ values favouring the species’ presence. This turns that Maxent algorithm has to find the distribution of predictor values that is the closest to *Q(z)*. The probability density function of these variables at the locations where the species is observed is *P(z)*, whereas P*(z) is the distribution we want Maxent calculates by forcing it to be as closed as possible to *Q(z)*. If we remember the definition of maximum entropy described above, Maxent has to find a P*(z) that also satisfies some constrains. These constrains are determined by the statical characteristics of the environmental values at the presence points. Maxent applies these constraints to the raw environmental variables and turns them into “features”, i.e., functions of original predictors. The details about the features are explained in Phillips et al.^63^ and Merow et al.^64^.

The mathematical formulation of Maxent model is the following, from Merow et al.^64^, Equation 1.

1 $${P}^{\ast }({{{{{\boldsymbol{z}}}}}}({x}_{i}))=\frac{Q({x}_{i})\exp ({{{{{\boldsymbol{z}}}}}}({x}_{i}){{{{{\boldsymbol{I}}}}}})}{{{\sum }^{}}_{i}Q({x}_{i})\exp ({{{{{\boldsymbol{z}}}}}}({x}_{i}){{{{{\boldsymbol{I}}}}}})}$$

where *z*(*x*_*i*_) is the vector of predictor/environmental values at location *x*_*i*_ and ***I*** is the vector of regressor coefficients. The aim of Maxent is to maximize the similarity between the first term of the equation (the distribution of environmental vales favouring the species’ presence/the prediction) and the second term, i.e., the constrained prior distribution. The prior distribution of the environmental variables is taken from a sample of the entire study area called background points. The way we choose these points can strongly affect the Maxent predictions^64^. Since the second term of the equation has as denominator the PDF of variables in the whole study area, the sum of all the P*(***z***(*x*_*i*_)) is 1^64^. This means that the final scores yielded by the algorithm are very small and difficult to interpret. Maxent turns these values into a logistic output that can be interpreted as Habitat Suitability Index scores^64,65^.

### Model cross-validation and parameter’ tuning

Classical calibration-testing protocols require the splitting of the occurrence dataset into training and testing data, the former used to build the mathematical model and the latter to measure the model’s prediction accuracy. This procedure is known as cross-validation and is particularly important when such models have to be transferred to different territories for conservation strategies or for reconstructing the temporal evolution of a species’ habitat, as in the current study. The calibrated model is then applied to reconstruct the known testing data and the related predictive performance is measured by specific indices, like AUC, Boyce, and TSS. The cross-validation is an iterative process, and its algorithm can be built according to the purposes of the research. In machine learning protocols, the cross-validation procedure has the important role of choosing the best combination of model parameters, thus allowing control for the model’s accuracy and complexity, whose balance has a prominent role for the model’s transferability^59,68^. The Maxent algorithm needs the tuning of different parameters, such as the kind of features to combine and to apply to the predictor environmental variables (i.e., the constraints discussed above)^65^ and a regularization multiplier, the function of which is to avoid model overfitting. We applied the Maxent algorithm of Phillips et al.^63^, as provided in the package “ENMeval” ver 2.0.3^69^ of the R software environment^42^. This package allows to set different cross-validation strategies for the tuning of all the model parameters (features and the regularization multiplier). We followed the strategy suggested by Helmstetter et al.^68^ and Santini et al.^59^, which resulted in the best way to tune a Maxent parameters in order to ensure transferability of the model through space and time. This procedure consists of dividing the occurrence data into different, spatially separated folds, using subsets of the data to calibrate the model. The remaining subsets of data were used to validate the model’s predictive performance by means of the AUC metric. The algorithm builds many Maxent models iteratively by testing different combinations of parameters and then provides the related performance metric. In the end, the user can choose the best performance model, i.e., that which most correctly predicts the occurrences in the testing dataset. This is a well-known “leave one out” strategy. Specifically, we divided the simulated occurrence dataset into twelve spatially separated groups, roughly coinciding with the polygons of the species’ spatial distribution provided by https://www.iucnredlist.org/ (the same used to simulate presences, as described above). Since these polygons have a north-south geographic distribution, we are sure that this spatial block strategy is the one that can ensure temporal model’s transferability. This strategy was used for both Hindcasting and Forecasting models.

### Background record simulation

Maxent algorithm is a presence-only method and only requires species’ occurrences and environmental data to work. As explained in Eq. 1, the species occurrence PDF needs to be regularized by dividing it by the PDF of environmental variables of the Landscape. Maxent uses a sample of the landscape represented by what are known as background point data. These points can be simulated over the study area following different strategies and should be sampled over the territory that could possibly host the species, although not found there. The background points could be sampled randomly over the study area, but this strategy implies that we know a-priori that the species could inhabit any area of the territory being studied. Since this is far from a true species’ natural distribution^63^, different strategies could be more suited to the ecology of the species being studied. We followed a strategy that simulates a random distribution of the background points but taking into account two constraints: (1) the occurrence probability of background points is inversely proportional to the distance to presence points; (2) the territory where we simulated the background points was not the whole of the area of present-day Ethiopia, but rather the territory enclosed by the biomes actually inhabited by the Ethiopian wolf plus the geographic distribution of its main prey, the big-headed African mole rat *Tachyoryctes macrocephalus*. To build this territory we used the world’s ecoregion polygons^70^. The range polygon of *Tachyoryctes macrocephalus* was downloaded from https://www.iucnredlist.org/. Once we have identified the polygons of the ecoregions intersecting the Ethiopian wolf spatial polygons, we combined them with the polygon of the big-headed African mole rat. Then we simulated several background points inside this unique combined polygon, verifying that there was only one background point per cell of the 4 × 4 km side resolution bioclimatic raster. We simulated 10,000 background points^59^.

### Statistical analyses

We used the predictions of the hindcasting and forecasting models to reconstruct the geographic range of the Ethiopian wolf through time and to estimate how much of the conditions suitable for the species habitat was present in the past and will be available to the species in the future scenarios.

#### Species geographic range and habitat suitability reconstructions in the past

At first, after computing the continuous habitat suitability maps for each time interval from 0 to 2 Ma, we extracted the HSI values corresponding to the geographic coordinates of the Melka Wakena fossil site and to the territory of the whole Ethiopia for comparisons. Then, we reported the time bins for which we recorded the highest and lowest HSI values for Melka Wakena site during its estimated age range.

For estimating the species geographic range during past time bins, it is important to remember that past bioclimatic rasters were provided by Raia et al.^55^ at a spatial resolution of 50 km side square cell—a rather coarse resolution compared to the 4 × 4 km cell raster used to train the models (see above). Hence, when comparing past and present habitat reconstructions, we used the projections we made of the models onto the present bioclimatic layers as provided by Raia et al.^55^. This allowed us to perform an informative comparison between the present and past suitable territories available to the species. At first, we reconstructed the temporal evolution of the species’ geographic range by binarizing the habitat suitability map obtained for each temporal interval. The binarization is obtained by considering all the cells in the raster map with a suitability index higher than a threshold value as a species geographic range. There are many strategies to find the most appropriate threshold value^71^, such as using the suitability value maximizing the difference between correctly identified true presences and true absences. Threshold values are computed by using the data for training the models and then applied to the habitat suitability maps. In our very case, since the bioclimatic variables of the present that were used for training the model and those of the past time intervals have different spatial resolutions, computing the threshold value by using the training data is not a valid strategy. Coarse-grained habitat suitability maps of the past might not include all the HSI values computed for the present temporal interval. Indeed, when we used threshold values computed with training data, many past temporal intervals yielded no (i.e., zero) geographic range of the species.

Here we used a special iterative strategy to compute a threshold value for successfully binarizing coarse-grained HSI maps computed with the bioclimatic rasters^55^. This strategy, which we dubbed Minimum Useful Threshold (MUT), prevented us from computing a null species geographic range for the past time intervals: (1) we created a vector of different thresholds ranging from 0.01 to 1, with a step of 0.01; (2) we applied each one of these threshold values to binarize the suitability maps computed from 0 to 2 Ma; (3) we counted the number of raster cell selected as species geographic range for each time bin. Then we chose as a valid threshold the lowest suitability value higher than zero geographic range in the complete considered time bin. We then applied this threshold to the continuous habitat suitability maps of all the time bins, to derive the species’ geographic range and to provide statistics about its temporal evolution. After computing the species’ geographic range, we tested if in the past time bins there were climatic conditions as suitable—or more beneficial—to the species as those in the current territory of Ethiopia. To this aim, we computed for each of the past 0.01 Ma-time interval a Wilcoxon test between the habitat suitability values sampled by the species in its estimated geographic range at the specific time bin and the same values sampled in the current geographic distribution of the species. In addition, we counted the time intervals for which we reconstructed statistically significant habitat suitability values similar to, or higher than, the computed ones for the present in the territory of Ethiopia. Since the amplitude of glacial cycles switched from a ~41 ka to ~100 ka at about 1 Ma, we hypothesized that the variance of the species’ geographic ranges computed for the time intervals younger than 1 Ma was higher than the variance during intervals older than 1 Ma. To test this hypothesis, we first averaged HSI values within the species’ geographic range in each time bin and then performed a Brown-Forsythe test for equality of variance, to compare the variances between the bins younger and older than 1 Ma. We performed the Brown-Forsythe test by using the R package “onewaytests” ver 2.6^29^.

Since Santini et al.^59^ warned about computing species’ geographic range by binarizing the habitat suitability maps according to threshold, we also computed for each time bin the surface area of cells in the maps with a specific range of suitability values. Specifically, we divided the past Ethiopian territories into two complementary spatial classes: one included cells with good-to-best habitat suitability values (GB, HSI values higher than 0.5) and another included cells with good-to-worst habitat conditions (GW, HSI values equals or lower than 0.5). Then, we counted the number of cells in each of the two spatial classes per time bin. Since our aim was to understand how many times in the last 2 Ma the species was in better and in worse conditions than at present, when a time bin showed a percentage of GB class higher than the GW we interpreted this information as a species geographic range expansion, whereas the reverse result indicated a stage of its range contraction.

This habitat suitability discrimination is important in order to understand under which bioclimatic conditions the species was able to survive. The procedure of HSI classification described above was applied to the portion of the territory of Ethiopia where we reconstructed the species’ geographic range by binarizing the habitat suitability map using the MUT strategy (described above). This geographic limitation was necessary because, in all of the time bins considered in this study, nearly the whole of the Ethiopia territory rendered very small suitability values, this biasing the relative abundance of the two HSI classes.

#### The relationship among the species’ geographic range evolution, the temperature changes, and the species’ altitude shift in the past

Since the Ethiopian wolf is known to be adapted to the Afroalpine habitat^72^ we tested the relationships between the changes in the species’ geographic range through time with the temperature variation in the last 2 Ma and the altitudes at which the species dwelt as response to climate change. Indeed, since the species currently inhabits high-altitude territories, we hypothesized that any size variation of past geographic ranges was determined by the species movement to higher elevations during unfavorable conditions during warm climates, and towards lower altitudes during periods of colder climates, when *C. simensi*s expanded its territories. In order to obtain a value for average temperature for each time bin, we extracted from the territory of Ethiopia the raster values of the annual maximum and minimum temperatures provided in Raia et al.^55^. Then, for each time bin, we averaged these two measures of temperature and get an annual mean temperature in Ethiopia related to time bins. For the geographic range of the species, we used the surface area of the HSI maps binarized by the MUT strategy, whereas for the altitude, we recorded the mean altitude value obtained by intersecting the species’ geographic range with the altitude raster map of the Ethiopia territory as provided by http://www.worldclim.org and rescaled to the same spatial resolution of Raia et al.^55^ bioclimatic raster maps. Then, we regressed the log_10_ of species geographic range surface area versus both the mean temperature values and log_10_ mean altitude per time bin.

As ours are time series data, we had to account for issues related to temporal auto-correlation in simple linear regressions’ residuals in order to have an accurate calculation of the regression coefficients^73^. This was done by applying an Auto Regressive Integrated Moving Average model (ARIMA)^73^. This model includes three different components to properly compute the regression’s coefficients. The auto regressive (AR) component allows to reduce the effect of auto-correlation in the series of observations, thus complying with the simple linear regression premise of independence of observations. Specifically, we calculated the order of the AR component that tells the ARIMA model how many consecutive values in the time series affect any single observation. For instance, an AR component of order 3 means that any observation can be predicted by considering the values of the 3 preceding observations in the time series^73^. The Integrated component of the model (I) allows detrending the time series in order to have stationarity in mean and variance of the measurements, whereas the moving average (MA) component allows smoothing the series values to reduce the outliers’ effect. At first, we performed an ordinary least square (OLS) regression between response (Log_10_ geographic range) and predictor variables (mean temperature and Log_10_ altitude). Then, we measured the degree of auto-correlation in model residuals. In the case of significant autocorrelations between these residuals, we used ARIMA models to properly compute the relationship between the response and predictor variables described above. We anticipate here that the residuals of the OLS showed significant autocorrelations.

We carried out two different strategies to set the parameters of ARIMA model components. In the first strategy, we used different combinations of parameters based on the OLS residuals time series. Only the AR component order of the OLS residuals was estimated automatically by means of maximum likelihood method. As regards the I component, since the time series of OLS residuals showed a clear auto-regressive component, we also used the first order (1) difference between models’ residuals to confer stationarity to the residuals’ distribution^73^. As regards the MA component, we performed six ARIMA models, each with fixed AR and I components computed as described above, but with different MA component values ranging from 0 (no moving average window) to 6 (i.e., by averaging up to 6 consecutive values in the time series). In the second strategy we estimated all the three ARIMA components by means of maximum likelihood estimation as implemented in *auto.arima* function in the package “forecast” ver 8.16^74^ of the software R^42^. After computing all the ARIMA models described in the two strategies, we chose the model with the lowest AIC.

#### Future suitable climatic conditions estimation

As mentioned above, since Santini et al.^59^ discouraged the use of a unique binarized suitability map to estimate a species’ range size, we considered intervals of different habitat suitability values separately to analyze the temporal evolution of some landscape metrics characterizing the territories suitable to *C. simensis* within the study area. For the present and for each of the future time bin maps, we considered four classes of habitat suitability value pixels: the first class, with the worst HSI values (HSI < 0.25); the second, with low values (HSI ≥ 0.25 and HSI < 0.5); a third one with good values (HSI ≥ 0.5 and HSI < 0.75); and a fourth class with the best habitat suitability conditions (HSI ≥ 0.75). For each of the habitat suitability classes, we computed the following landscape structural connectivity indices. We did not compute any measure of distance between patches since, given that *C. simensis* occupies mountain tops, differences of HSI values between near patches may be strongly affected by the steep altitudinal variation. The indices used in this analysis were: the number of patches (“np”); mean patch surface area (“area_mn”); Patch aggregation (“ai”) is the ratio between the number of the adjacent patches of the same class and the maximum possible number of like adjacencies involving the corresponding class, which is achieved when the class is maximally clumped into a single, compact patch. Its values range from 0 to 100; Clumpiness (“clumpy”) indicates how many equal-class patches are spatially adjacent if compared to a totally random spatial distribution of all the available different classes and its values can range from −1 to 1; Cohesion (“cohesion”) measures the physical connectedness of the corresponding patch class (values ranging from 0 to 100); the Division index (“division”, ranging from 0 to 1) is the probability that two randomly chosen places in the landscape are not situated in the same undissected patch^75^. We computed landscape metrics by using the R package “landscapemetrics” ver 1.5.4^76^.

We then used these landscape metrics to test the statistical differences between the CMIP6 future climatic projections while considering the related four shared socio-economic pathways (ssp) as repeated experiments. For this, we set a two-way permutational MANOVA (two-way PerMANOVA) in which the landscape metrics were the explanatory variables, the four CMIP6 models and the related future time bins were the factors and the four shared socio-economic pathways (ssp) were experiment’s stratifications, i.e., repeated measures. PerMANOVA allows testing differences between groups with small sample size and without the assumption of normal distribution and equality of variance by relying on the computation of between-groups distance matrix and permutations. We considered the Log_10_ of all the used landscape metrics and computed the between-groups distance matrices by using the Euclidean distance. *p*-values were computed after setting 9999 permutations. Where significant differences were detected, a pairwise version of the two-way PerMANOVA with stratification was applied. We performed two-way PerMANOVA by using the R package “vegan” ver 2.6-2^77^ and the related pairwise version by means of the “pairwiseAdonis” package ver 0.4^78^.

The following analyses were performed for each of the considered CMIP6 future climatic scenario projections, separately, since these models come from different starting hypotheses and physical processes the laboratories considered for their modeling experiments. Then we used the landscape metrics to analyze the temporal variation (including the metrics of the present time interval) of the mean patch surface area (“area_mn”), while accounting for the differences between the HSI classes. More specifically, we performed four simple linear regression models, one for each of the CMIP6 future projections, in which we considered the Log_10_ of the mean patch surface area as a response variable, the temporal step of the models’ projections (from the present to the future) and the HSI classes as explanatory variables.

Further, we tested the relationships between the four HSI classes and the landscape metrics to investigate if patches of species habitat suitability classes are actually exploitable by the species in the present, and whether they will be so in the future. We set four Generalized Linear Model (GLM) regressions in which the HSI classes were the response variables, and all the landscape metrics were the explanatory variables. In these regression models, in order to take into account the temporal variation of the variables, all the explanatory variables were considered in interaction with the time bins of the climatic projections, from the present to the future. We emphasize that, since *C. simensis* exists in a very restricted territory of the Ethiopian highlands, most of the geographic surface considered in this study provides unfavorable bioclimatic conditions. Most of the area of Ethiopia shows low HSI values (falling within HSI class 1; see above), i.e., are barely suitable for *C. simensis*. This overabundance of raster pixels of low HSI values occurred in the map of present conditions and in any of the considered future climatic scenarios and might have masked any between-CMIP6 model differences and statistical relationships. Therefore, we excluded the HSI class 1 values from PerMANOVA and regression analyses. Moreover, since some HSI classes totally disappeared (area_mn= 0) for two CMIP6 future projections, it was impossible to compute landscape metrics for these cases and data for the analyses were unbalanced. Therefore, we first standardized all data in a way such that all the CMIP6 model-related projections had the same HSI classes computed and then performed all the statistical analyses. We did not apply this correction to the four regressions describing the relationship between mean patch area and time and HSI classes, since these analyses do not require the computation of the other landscape metrics. It is important to note that, for all the same analyses, we did not consider the landscape metric “division” since it showed zero variance.

We emphasize that our present and future habitat suitability projections as well as the computation of the landscape metrics were not conducted with the aim of providing any strategy for the species conservation plans. They did turn out to highlight the acute threat that the Ethiopian wolf is already subjected to and the growing risks it would be exposed to in the near future.

### Statistics and reproducibility

#### Morphometric study of the fossil

Morphometric analyses were performed with SPSS statistics program ver. 25, and with Rv. 3.6.0^42^ in RStudio ver. 2021.09.1 + 372 “Ghost Orchid” (Release 8b9ced188245155642d024aa3630363df611088a, 2021-11-08) for Windows using the packages “ggfortify” (ver. 0.4.013)^43,44^, “ggplot2” (ver. 3.3.5)^45^, “pvclust” (ver. 2.2-0)^46,47^, and “stats” (ver. 4.1.2.)^42^.

#### Modeling the bioclimatic niche through time

##### Simensis polygon

We simulated 200 *Canis simensis* occurrences over the grid of the bioclimatic variables by considering the geographic range polygon downloaded from IUCN (https://www.iucnredlist.org/species/3748/10051312) as spatial boundaries. For the present and future time intervals, we used the bioclimatic variables at the spatial resolution of 1 × 1 and 4 × 4 km wide cell side (www.wrldclim.org) combined with a rescaled raster of the Net Primary Productivity (https://cmr.earthdata.nasa.gov/search/concepts/C1631984056-LPDAAC_ECS.html), whereas for the past time bins, we used the variables as provided by Raia et al.^55^ with the spatial resolution of 50 × 50 km wide cell side. To train and calibrate Maxent algorithm for the Species Distribution Modeling, we used the “ENMeval” v. 2.0.3^69^.

The ARIMA regressions used for investigating the relationship among the species’ geographic range evolution, the temperature changes, and the species’ altitude shift in the past were performed by using the “forecast” package v. 8.16^74^.

Habitat fragmentation metrics were performed by using the “landscapemetrics” package v. 1.5.4^76^.

We performed two-way PerMANOVA analysis by using the package “vegan” v. 2.6-2 and the related pairwise version with the “pairwiseAdonis” package ver 0.4^78^.

All the statistical analyses were performed with the R statistical software v. 4.1.0^42^.

### Reporting summary

Further information on research design is available in the Nature Portfolio Reporting Summary linked to this article.

## Supplementary information

Supplementary Information

Reporting Summary

## Data Availability

The codes and all the information to run the analyses with ecological data and landscape metrics for present, past and future time intervals, are freely available and stored at https://zenodo.org/badge/latestdoi/618339598. The codes run under the free R statistical software and include the lines to install the necessary packages to perform the analyses.

## References

[1] Yalden DW (1983). The extent of high ground in Ethiopia compared to the rest of Africa. Sinet: Ethiop. J. Sci..

[2] Malcolm, J. R. & Ashenafi, Z. T. In *The Ethiopian Wolf: Status Survey And Conservation Action Plan* (eds. Sillero-Zubiri, C. & Macdonald, D. W.) 61–63 (IUCN/SSC Canid Specialist Group, 1997).

[3] Sillero-Zubiri, C., Marino, J., Gottelli, D. & Macdonald, D. W. In *The Biology and Conservation of Wild Canids* (eds. Sillero-Zubiri, C. & Macdonald, D. W.) (Oxford University Press, 2004).

[4] Marino, J. & Sillero-Zubiri, C. *Canis simensis. The IUCN Red List of Threatened Species Version 20112*. 10.2305/IUCN.UK.2011-1.RLTS.T3748A10051312.en (2011).

[5] Gottelli D, Sillero-Zubiri C, Marino J, Funk SM, Wang J (2013). Genetic structure and patterns of gene flow among populations of the endangered Ethiopian wolf. Anim. Conserv.

[6] Castelló, J. R. *Canids of the World: Wolves, Wild Dogs, Foxes, Jackals, Coyotes, and Their Relatives* Vol. 135 (Princeton University Press, 2018).

[7] EWCP Annual Report 2020. www.wildcru.org/news/ewcp-annual-report-2020-now-out (2020).

[8] Atickem A, Stenseth NC (2022). The role of rodents in the conservation of endangered species in the Ethiopian highlands. Therya.

[9] Traill LW, Bradshaw CJ, Brook BW (2007). Minimum viable population size: a meta-analysis of 30 years of published estimates. Biol. Conserv..

[10] Ferràndez-Cañadell C, Ribot F, Gibert L (2014). New fossil teeth of *Theropithecus oswaldi* (Cercopithecoidea) from the Early Pleistocene at Cueva Victoria (SE Spain). J. Hum. Evol..

[11] Gottelli D (1994). Molecular genetics of the most endangered canid: the Ethiopian wolf *Canis simensis*. Mol. Ecol..

[12] Gottelli D, Marino J, Sillero‐Zubiri C, Funk SM (2004). The effect of the last glacial age on speciation and population genetic structure of the endangered Ethiopian wolf (*Canis simensis*). Mol. Ecol..

[13] Hovers E (2021). The expansion of the Acheulian to the Southeastern Ethiopian Highlands: insights from the new early Pleistocene site-complex of Melka Wakena. Quat. Sci. Rev..

[14] Lindblad‐Toh K (2005). Genome sequence, comparative analysis, and haplotype structure of the domestic dog. Nature.

[15] Gopalakrishnan S (2018). Interspecific gene flow shaped the evolution of the genus. Canis. Curr. Biol..

[16] Ciucani MM (2021). Evolutionary history of the extinct Sardinian dhole. Curr. Biol..

[17] Perri AR (2021). Dire wolves were the last of an ancient NewWorld canid lineage. Nature.

[18] Azzaroli A (1983). Quaternary mammals and the end-villafranchian dispersal event—a turning point in the history of Eurasia. Palaeogeo. Palaeoclim. Palaeoecol..

[19] Tedford RH, Wang X, Taylor BE (2009). Phylogenetic systematics of the North American fossil caninae (Carnivora: Canidae). Bull. Am. Mus. Nat. Hist..

[20] Bartolini-Lucenti, S., Bukhsianidze, M., Martínez-Navarro, B. & Lordkipanidze, D. The wolf from Dmanisi and augmented reality: review, implications, and opportunities. *Front. Earth Sci. Paleont.* **8**, 131 (2020).

[21] Martinez-Navarro B (2021). A new species of dog from the Early Pleistocenesite of Venta Micena (Orce, Baza Basin, Spain).. C. R. Palevol..

[22] Bartolini-Lucenti S (2021). The early hunting dog from Dmanisi with comments on the social behaviour in Canidae and hominins. Sci. Rep..

[23] Martínez-Navarro B, Rook L (2003). Gradual evolution in the African hunting dog lineage systematic implications. C. R. Palevol..

[24] Lordkipanidze D (2007). Postcranial evidence from early Homo from Dmanisi, Georgia. Nature.

[25] Lordkipanidze D (2013). A complete skull from Dmanisi, Georgia, and the evolutionary biology of early. Homo. Sci..

[26] Xue L, Alemu T, Gani ND, Abdelsalam MG (2018). Spatial and temporal variation of tectonic uplift in the southeastern Ethiopian Plateau from morphotectonic analysis. Geomorphology.

[27] Marino J (2003). Threatened Ethiopian wolves persist in small isolated Afroalpine enclaves. Oryx.

[28] Bonnefille, R., Melis, R. & Mussi, M. in *The Emergence of the Acheulean in East Africa and Beyond. Vertebrate Paleobiology and Paleoanthropology* (eds. Gallotti, R. & Mussi, M.) (Springer, 2018).

[29] Dag O, Dolgun A, Konar NM (2018). Onewaytests: an R package for one-way tests in independent groups designs. R. J..

[30] Mondanaro A (2021). The role of habitat fragmentation in Pleistocene megafauna extinction in Eurasia. Ecography.

[31] Mooney, J. A., Marsden, C. D., Yohannes, A., Wayne, R. K. & Lohmueller, K. E. Long-term small population size, deleterious variation, and altitude adaptation in the ethiopian wolf, a severely endangered canid. *Mol. Biol. Evol*. **40**, msac277 (2022). 10.1093/molbev/msac277 PMC9847632 36585842

[32] Abrahms B, Rafiq K, Jordan NR, McNutt JW (2022). Long-term, climate-driven phenological shift in a tropical large carnivore. Proc. Natl Acad. Sci. USA.

[33] Weber MM, Stevens RD, Diniz‐Filho JAF, Grelle CEV (2017). Is there a correlation between abundance and environmental suitability derived from ecological niche modelling? A meta‐analysis. Ecography.

[34] Noto, C. R. In *Taphonomy* 287–336 (Springer, 2011).

[35] Carotenuto F (2020). MInOSSE: a new method to reconstruct geographic ranges of fossil species. Meth. Ecol. Evol..

[36] Geraads D (2011). A revision of the fossil Canidae (Mammalia) of North-western Africa. Palaeontology.

[37] Werdelin, L. & Lewis, M. E. *Koobi Fora Research Project, Volume 7, the Carnivora* 333p (California Academy of Sciences, 2013).

[38] Werdelin, L., Lewis, M. E. & Haile-Selassie, Y. New species of *Eucyon* (Mammalia; Carnivora; Canidae) from the Pliocene of the Woranso-Mille Area, Afar Region, Ethiopia, and a critical review of African species of *Eucyon*. *Pap. Palaeontol.* **1**, 33–40 (2015).

[39] Ewer, R. F. The fossil carnivores of the Transvaal caves: Canidae. *Proc. Zool. Soc. London* **126**, 97–120 (1956).

[40] Adams JA, Olah A, McCurry MR, Potze S (2015). Surface model and tomographic archive of fossil primate and other mammal holotype and paratype specimens of the Ditsong National Museum of Natural History, Pretoria, South Africa. PLoS ONE.

[41] A. von den Driesch, A guide to the measurement of animal bone from archaeological sites. Peabody Museum Bulletin 1 (Peabody Museum of Archaeology and Ethnology, 1976).

[42] R. Core Team R: A language and environment for statistical computing. R Foundation for Statistical Computing, Vienna, Austria. https://www.R-project.org/ (2021).

[43] Horikoshi, M. & Tang, Y. ggfortify: Data visualization tools for statistical analysis results. https://CRAN.R-project.org/package=ggfortify (2016).

[44] Tang Y, Horikoshi M, Wenxuan L (2016). ggfortify: unified interface to visualize statistical result of popular R packages. R. J..

[45] Wickham, H. *ggplot2: Elegant Graphics for Data Analysis* (Springer-Verlag New York, 2016).

[46] Suzuki R, Shimodaira H (2006). Pvclust: an R package for assessing the uncertainty in hierarchical clustering. Bioinformatics.

[47] Suzuki, R., Terada, Y. & Shimodaira, H. pvclust: hierarchical clustering with p-values via multiscale bootstrap resampling. R package version 2.2-0. https://CRAN.R-project.org/package=pvclust (2019).

[48] Sokal RR, Michener CD (1958). A statistical method for evaluating systematic relationships. Univ. Kans. Sci. Bull..

[49] DePatta Pillar V (1999). How sharp are classifications?. Ecology.

[50] Elith J, Leathwick JR (2009). Species distribution models: ecological explanation and prediction across space and time. Ann. Rev. Ecol. Evol. Syst..

[51] Araújo MB (2019). Standards for distribution models in biodiversity assessments. Sci. Adv..

[52] Chiarenza AA, Mannion PD, Farnsworth A, Carrano MT, Varela S (2022). Climatic constraints on the biogeographic history of Mesozoic dinosaurs. Curr. Biol..

[53] Cunze S, Klimpel S (2022). From the Balkan towards Western Europe: Range expansion of the golden jackal (Canis aureus). A climatic niche modeling approach. Ecol. Evol..

[54] Fick SE, Hijmans RJ (2017). WorldClim 2: new 1km spatial resolution climate surfaces for global land areas. Int. J. Climatol..

[55] Raia P (2020). Past extinctions of Homo species coincided with increased vulnerability to climatic change. One Earth.

[56] Holden PB (2019). PALEO-PGEM v1. 0: a statistical emulator of Pliocene–Pleistocene climate. Geosci. Model Dev..

[57] Meinshausen M (2020). The shared socio-economic pathway (SSP) greenhouse gas concentrations and their extensions to 2500. Geosci. Model Dev..

[58] IPCC. *Climate Change 2014: Synthesis Report. Contribution of Working Groups I, II and III to the Fifth Assessment Report of the Intergovernmental Panel on Climate Change* (eds. Core Writing Team, R. K. Pachauri, R. K. & Meyer, L. A.) 151pp (IPCC, 2014).

[59] Santini L, Benítez‐López A, Maiorano L, Čengić M, Huijbregts MA (2021). Assessing the reliability of species distribution projections in climate change research. Divers. Distrib..

[60] Elith J, Kearney M, Phillips S (2010). The art of modelling range‐shifting species. Meth. Ecol. Evol..

[61] Zuur AF, Leno EN, Elphick CS (2010). A protocol for data exploration to avoid common statistical problems. Meth Ecol. Evol..

[62] Breiner FT, Guisan A, Bergamini A, Nobis MP (2015). Overcoming limitations of modelling rare species by using ensembles of small models. Meth. Ecol. Evol..

[63] Phillips SJ, Anderson RP, Schapire RE (2006). Maximum entropy modeling of species geographic distributions. Ecol. Mod..

[64] Merow C, Smith MJ, Silander JA (2013). A practical guide to MaxEnt for modeling species’ distributions: what it does, and why inputs and settings matter. Ecography.

[65] Elith J (2011). A statistical explanation of MaxEnt for ecologists. Divers. Distrib..

[66] Jaynes ET (1957). Information theory and statistical mechanics. Phys. Rev..

[67] Shannon CE (1948). A mathematical theory of communication. Bell Syst. Tech. J..

[68] Helmstetter NA, Conway CJ, Stevens BS, Goldberg AR (2021). Balancing transferability and complexity of species distribution models for rare species conservation. Divers. Distrib..

[69] Kass, J. M. et al. ENMeval 2.0: redesigned for customizable and reproducible modeling of species’ niches and distributions. *Meth. Ecol. Evol*. 1602–1608, 10.1111/2041-210X.13628 (2021).

[70] Dinerstein E (2017). An ecoregion-based approach to protecting half the terrestrial realm. BioScience.

[71] Scherrer D, D’Amen M, Fernandes RF, Mateo RG, Guisan A (2018). How to best threshold and validate stacked species assemblages? Community optimisation might hold the answer. Meth. Ecol. Evol..

[72] Marino J, Sillero‐Zubiri C, Macdonald DW (2006). Trends, dynamics and resilience of an Ethiopian wolf population. Anim. Conserv.

[73] Cowpertwait, P. S. & Metcalfe, A. V. *Introductory Time Series with R* (Springer Science & Business Media, 2009).

[74] Hyndman RJ, Khandakar Y (2008). Automatic time series forecasting: the forecast package for R. J. Stat. Softw..

[75] McGarigal, K. FRAGSTATS: Spatial Pattern Analysis Program for Categorical Maps. Computer software program produced by the authors at the University of Massachusetts, Amherst. http://www.umass.edu/landeco/research/fragstats/fragstats.Html (2002).

[76] Hesselbarth MH, Sciaini M, With KA, Wiegand K, Nowosad J (2019). Landscapemetrics: an open‐source R tool to calculate landscape metrics. Ecography.

[77] Oksanen, J. et al. vegan: Community Ecology Package. R package version 2.5-6. 2019 (2020).

[78] Martinez Arbizu, P. pairwiseAdonis: Pairwise multilevel comparison using adonis. R package version 0.0, 1 (2017).

