# Supplementary material for: The earliest Ethiopian wolf: implications for the species evolution and its future survival
Source: Commun Biol. 2023 May 16;6:530. doi: 10.1038/s42003-023-04908-w (PMC10187515; doi:10.1038/s42003-023-04908-w)
Supplement: Supplementary file 1 — Supplementary Information [file 42003_2023_4908_MOESM1_ESM.pdf]

Supplementary Information

The earliest Ethiopian wolf: Implications for the species evolution and its future survival

Authors: Bienvenido Martínez-Navarro[1,2,3,*], Tegenu Gossa[4,5,6,**], Francesco Carotenuto[7], Saverio Bartolini-Lucenti[8,9], Paul Palmqvist[10], Asfawossen Asrat[11,12], Borja Figueirido[10], Lorenzo Rook[8], Elizabeth M. Niespolo[13], Paul R. Renne[14,15], Gadi Herzlinger[5], Erella Hovers[5,16,***]

1) ICREA, Pg. Lluís Companys 23, 08010 Barcelona, Spain
2) Institut Català de Paleoecologia Humana i Evolució Social (IPHES-CERCA), Zona Educacional 4, Campus Sescelades URV (Edifici W3), 43007 Tarragona, Spain.
3) Universitat Rovira i Virgili, Departament d'Història i Història de l'Art, Avinguda de Catalunya 35, 43002 Tarragona, Spain
4) Human Evolution Research Center (HERC), The University of California at Berkeley, CA, USA
5) Institute of Archaeology, The Hebrew University of Jerusalem, Israel
6) Department of History and Heritage Management, Arba Minch University, Ethiopia
7) Department of Earth, Environment and Resource Sciences, University of Naples "Federico II", Naples, Italy
8) Earth Science Department, Paleo[Fab]Lab, University of Florence, Via G. La Pira 4, Firenze, 50121, Italy
9) Institut Català de Paleontogia M. Crusafont, Universitat Autònoma de Barcelona, E-08193 Cerdanyola del Vallès, Spain
10) Departamento de Ecología y Geología, Universidad de Málaga, Campus de Teatinos, 29071 Málaga, Spain
11) Department of Mining and Geological Engineering, Botswana International University of Science and Technology, Private Bag 16, Palapye, Botswana
12) School of Earth Sciences, Addis Ababa University, P. O. Box 1176, Addis Ababa, Ethiopia
13) Department of Geosciences, Princeton University, Princeton, NJ, USA
14) Department of Earth and Planetary Science, University of California, Berkeley, CA, USA
15) Berkeley Geochronology Center, Berkeley, CA, USA
16) Institute of Human Origins, Arizona State University, Tempe, USA

Corresponding authors: * bienvenido.martinez@icrea.cat

** tegenugossa@berkeley.edu

*** hovers@mail.huji.ac.il

Supplementary Information contains:

Supplementary note 1. Background

Supplementary note 2. The stratigraphic series where the *Canis simensis* fossil mandible MW5-B208 was recovered: MW5-West (MW5-W)

Supplementary note 3. Phylogenetic relationships of extant *Canis simensis*: a short overview

Supplementary note 4. Systematic Paleontology

4.1. Material

4.2. Description

4.3. Comparison to extant African canids

4.3.1. Comparison to the extant Lupulella species

4.3.2. Comparison to extant African Canis species

4.3.3. Comparison to selected fossil canids species

4.3.4. Morphometric analyses and BCA of Melka Wakena mandible

Supplementary Note 5: Results of the modelling analyses

5.1. The Hindcasting modelling

5.2. Estimating the species' habitat status in the past

5.3 The relationship between the species' geographic range evolution and the temperature changes and the species' altitude shift in the past

5.4. The Forecasting modelling

Supplementary Figures 1 –5.

Supplementary Tables 1 – 6.

Suplementary references

Suppementary note 1. Background

The Melka Wakena[1] (MW) archaeological site-complex was discovered in 2014, following reports from local farmers of fossils sightings. Initial feldwork and test excavations were carried out during 2015-2017 at three archaeological localities[1,2]. Additional localities were recognized on both the western and eastern banks but to date these have not been explored.

The site-complex is located at an elevation of 2300-2350 m above sea level (7°05'03.00'' N, 39° 16'01.00'' E) on the floodplain of the upper Wabe river, the headwater of the Wabe Shebele drainage system. The Wabe floodplain lays between the foothills of the Bale Mountains (>4000 m asl) in the east and the Main Ethiopian Rift (MER) (~1700 m asl) to the west (Figs. 1A-B and S1). The regional topography of the basin has been relatively stable since the Oligocene[3]. The most recent regional tectonic uplift affecting the South Eastern Highlands (SEH) dates back to 4.5 Ma, after which the regional topography has been established broadly to its present configuration[4]. Miocene - Pliocene felsic pyroclastic deposits of the Nazareth Series form the extensive low-lying plains of the eastern rift margin and the adjoining highland plains, including the MW study area, dotted with late Pliocene, off-rift-axis volcanic shields[5-7] raising to more than 3600 m asl. Subsequent volcanism, associated with faulting of the MER, took place ~1.6 Ma or [6-9], resulting in deposition of the Early-Middle Pleistocene Dino Formation[10] that is exposed on both rift margins.

The modern climate of the SEH is influenced by the Indian Monsoon and the nearly N-S movement of the Intertropical Convergence Zone (the African rain belt) resulting in a bimodal rainfall regime[11]. Temperatures remain relatively constant throughout the year, averaging 16.9°C the warmest month (March) and 14.6°C the coldest (November). Precipitation occurs as rain and dew, with significant variation in monthly means ranging from 177.8 mm in August to 17.8 mm in December. A major summer (June-September) rainy season and a smaller spring (March-April) rainy season are clearly distinguished.

MW is situated within one of the largest enclaves of the 'Afromontane Archipelago' in Africa, which consists of unique high elevation plant communities of the tropics that are separated by low-lying areas[12-14]. It has been suggested that characteristics of the dry evergreen Afromontane forest/ grassland complex were established on the Ethiopian highlands since ca. 1.8 Ma[15] (Supp. Fig. 1). These authors linked temporal change in pollen abundances at the site of Melka Kunture (MK) to orbitally-forced climatic events and suggested that the expansion of the grasslands occurred during cool and dry climate conditions[16,17]. At the same time, they noted in their MK samples broadly synchronous variation over small spatial scales and the patchiness of the true juniper at the time of the earliest Acheulian settlement (ca. 1.6 Ma).

The specimen MW5-B208 was found ca. 90 meters due west of the archaeological locality MW5 (GPS location: N 7º05'16.83" E 39º16'10.06"), lying directly on a tephra dated to 1626.6 +/- 6.4 ka (Unit I)[1]. Its youngest possible age is constrained by the 1372.5 +/- 4.8 ka age (Hovers et al., 2021) of the overlying Unit II (Fig. 1c-d). The ages were calibrated using the Alder Creek Sanidine fluence monitor age of 1.1891 Ma[18,19].

The hominin occupations at the MW site-complex are associated with fluvial contexts, inferred from both the sedimentology, lithic taphonomy and fossil fauna. The latter is dominated by the abundance of two large water-dependent species, *Hippopotamus gorgops* and *Crocodylus* cf. *niloticus*, although there are other species representative of the savannah grassland and forest[1]. All the archaeological horizons are assigned to the Acheulian technocomplex. The assemblages are characterized by the presence of large and giant cores, giant flakes and bifacial tools (cleavers and handaxes) as well as a system for small debitage production. These typo-technological characteristics as well as geochronology place all the occurrence within the earlier Acheulian[1-,20,21], similar to other highland and MER sites[22-33].

Supplementary note 2. The stratigraphic series where the *Canis simensis* fossil mandible MW5-B208 was recovered: MW5-West (MW5-W)

The general stratigraphic sequence at the MW site-complex consists of a ~30 m thick succession of pyroclastic and reworked volcaniclastic deposits and generally unconsolidated fluvial sediments. Fluvio-lacustrine sediments are rare. The units show sharp to gradational contacts within a short vertical succession, and nearly all units are laterally discontinuous. Some units pinch out laterally at short distances (a few meters to tens of meters), while others are structurally truncated against other units[2]. The sequential aggradation and erosion in fluvial environments have been the pre-requisite for favorable preservation of the archaeological record[1].

The stratigraphic section at MW5-W, ca. 90 meters west of the MW5 archaeological locality, consists of pyroclastic units interspersed with volcaniclastic sediments (Figs. 1C-D, and Supp. Fig. 2). From bottom to top:

1. Unit I (Fig. 1d) is an unwelded tuff/ash fall deposit. The full thickness of the unit remains unknown because its base is under water. The ash shows minor signs of vesiculation. Imprints of grass roots are evident towards the top of the unit. The specimen MW5-B208 was found on the surface of this unit, i.e., on the lowermost geological unit of the stratigraphic sequence of the Downstream section, exposed due to disintegration of sediments of *Units II* (Supp. Fig. 2).
2. *Unit II* is a c. 30 cm-thick, poorly sorted fine conglomerate that consists of poorly sorted sub-angular (basically ignimbrites) and sub-rounded and rounded clasts (mostly basalts) of 1-3 cm in diameter, associated with an ash in a silty to sandy matrix[34]. Lithic artifacts and faunal remains found at the top of the unit in a ca. 10 cm thick layer constitute the archaeological layer MW5-L3 (Supp. Fig. 2b)[1].
3. *Unit III* is a ca. 2.5 m-thick, grey, crystal-poor and fine-grained ash fall with little weathering.This unit provides the minimum possible age of 1,372.5±4.8 ka for MW5-B208. Field observations suggest, however, that the fossil is closer in age to *Unit I* (Fig. 1d and Supp. Fig. 2).

4. *Unit IV* is a ca. 30 cm-thick deposit of reworked sand, which in lateral extension can be 1.5 m-thick. This poorly sorted coarse sand layer contains a mixture of reworked ash, gravels and pebbles. The archaeological locality has two archaeological layers (i.e., MW5-L2 and MW5-L1)[1,20]. Such significant thickness variations over short distance suggest that this unit represents an erosional surface, where repeated truncations, re-depositions and lateral mixing by active channels were improtant.
5. *Unit V* is a ca. 80 cm-thick unit of grey, crystal-poor and weathered ash flow. This unit is exposed along the cliff face of MW5-W as a laterally non-continuous unit. This unit appears to have undergone extensive post-depositional erosion[34], and is missing from the sequence at the MW5 archaeological locality. This unit is dated to 1342.9±15.0 ka (Fig. 1d).
6. *Unit VI* is a poorly sorted layer ca. 50 cm-thick, composed of clay, silt and gravels. It was likely formed as sediment reworking by a high-energy fluvial system.
7. *Unit VII* is a roughly 2.5 m-thick layer of a primary fine-grained bluish ash fall, rich in visible shards. At the MW5 archaeological locality, the unit is represented by a small, non-continuous pinching structure dated to 1251.7±4.8 ka.
8. *Unit VIII* is a ca. 1.5 m-thick layer of moderately welded ignimbrite that caps the sequence.

The alternation of eruption and maximum ages along the depositional series shows that fluvial processes and subsequent depositional events occurred in the context of active volcanic and hydrological processes. All hominin occupations at the site are associated with fluvial contexts.

Supplementary note 3. Phylogenetic relationships of extant *Canis simensis*: a short overview

Molecular analyses on *Canis simensis* were first performed during the mid1990s[35] and revealed a strong relationship between this species, the gray wolf (*Canis lupus*) and coyotes (*Canis latrans*) rather than to other African canids. Subsequent, more comprehensive analyses confirmed the ascription of *C. simensis* to what can be defined the crown-group *Canis*, i.e., the group including *C. lupus*, *C. latrans*, the African golden wolf *C. lupaster*, the golden jackal *C. aureus*[36-40],. Particularly, *C. simensis* stems at the base of this clade[37,41] (See the cladogram in Supp. Fig. 3). Some authors have pointed out the importance of the Ethiopian wolf in terms of gene flow contribution to the diversity pool of the African members of the genus *Canis*[37]. Indeed, *C. lupaster* is considered as a species that arose from the admixture of *C. lupus* and *C. simensis*. In turn, it is reasonable to think of *C. simensis* as a canid that arose from Eurasian *Canis* s.s. stock, as the center of origin and dispersal of *Canis* s.s. is unanimously recognized in Eastern Asia[42]. At present there is no certainty on a plausible fossil species that was the ancestor of the Ethiopian wolf, although it is clear from the molecular evidence that *C. simensis* had a pretty straightforward ascendence with few occasional admixture events with the African Hunting dog (*Lycaon pictus*)[41]. Following molecular clock techniques, the divergence time for *C. simensis* from the other species of the crown-group *Canis* is estimated around 2.5 Ma[40,43]. Such estimates are in marked contrast with the previous interpretations that focused on limited parts of the Ethiopian wolf genome, e.g., mitochondrial DNA. Indeed, such studies proposed a much more recent time of differentiation of *C. simensis* from a wolf-like ancestor, during the early Late Pleistocene, around 100 ka[35]. The evidence on the relationships between the different extant species of crown-group *Canis* allow neontologists as well as paleontologists to reject this hypothesis, considering the number of species more related to wolf than to *C. simensis* (i.e., all diverged after the Ethiopian wolf, like *C. aureus*, *C. lupaster*, *C. latrans*) or the well-documented presence of fossil *C. lupus* and *C. latrans* during the mid-Middle Pleistocene of Eurasia. Although rigorously tested and calculated, the hypotheses on *C. simensis*' time of divergence as

inferred from the molecular data lacked empirical evidence. The first discovery of a fossil *C. simensis* in Ethiopia, at 1.4-1.6 Ma, with all the dentognathic peculiarities of its extant relatives and very few differences, constitutes the support needed for the genetic interpretations for this canid, in terms of divergence and of relationships.

Supplemetary note 4. Systematic Paleontology

Class Mammalia Linnaeus, 1758

Order Carnivora Bowdich, 1821

Family Canidae Fischer, 1817

Subfamily Caninae Fischer, 1817

Tribe Canini Fischer, 1817

Genus *Canis* Linnaeus, 1758

Canis simensis Rüppell, 1840

Synonym *Canis* sp. (Hovers et al.[1])

4.1. ***Material***

A mostly complete right hemimandible without the coronoid process, coming from geological unit II, of MW5-West, that preserves c, roots of p1, p2, and p3, p4, m1, m2, and alveolus of m3 (Figs. 2 and 3, Supp. Table 1).

4.2. ***Description***

It is an elongated mandible of a medium-size canid, similar to the extant endemic Ethiopian species *Canis simensis*. The external surface of the mandible is almost completely worn out and highly weathered. It is rostrally broken, missing the alveoli of the incisors and portion of the symphysis. The dental pieces from c1 to p4 are separated by diastemata and it shows two nutritional foramina, one below the p1 and the other below the diastema between p2 and p3. The canine is broken without the tip, showing a robust oval-base. The first three lower premolars are missing but the roots are present in the alveoli. The p1 is single rooted and the others are bi-rooted, with the mesial root smaller and round compared to distal one, which is larger and oval. The fourth premolar, p4, possesses high principal cuspid, with two distal cuspulids, and small mesial and distal cingulum, with a solid base in the labial face. The lower carnassial, m1, has developed trigonid

with a high protoconid and lower but conspicuous paraconid; the metaconid is marked but not developed. The talonid is bicuspid, with the hypoconid larger and linked to the smaller entoconid, by a transverse cristid. The entoconid is also connected to a mesial reduced cuspulid by another cristid. The second molar, m2, shows a large mesial cristid (paracristid), and has four prominent cuspids which are rather worn down.

4.3. ***Comparison to the extant African canids***

4.3.1. *Comparison to extant* Lupulella *species*

Compared to other extant African canids, the outline of MW5-B208 mandible in buccal view is peculiar (Figs. 2 and 3). The corpus is too shallow and elongated rostrocaudally in comparison to that of *Lupulella adusta* and *Lupulella mesomelas* (Fig. 3i-n). In these canids the mandible has a scaphoid shape and its ventral margin is definitely arched, with ventral convexity, especially in *L. mesomelas*. The position of the mental foramina of MW5-B208 are peculiar and differ from those of *Lupulella* spp., in which the larger and rostral one lies under the distal side of the p1 and the smaller and caudal one is at the level of the mesial root of the p3. Moreover, *Lupulella* do not have diastemata between premolars, not as wide and not as many as in MW5-B208. Dentally, *L. adusta* and *L. mesomelas* have high-crowned premolars, with a conspicuous distal accessory cuspulid on the p4 and slender base of the crown. This contrasts with the morphology of MW5-B208. Although the lower canine of MW5-B208 is broken, it contrasts with the shape of that *L. adusta* that is large at the base, very elongated and arched. In its morphology, the canine of MW5-B208 resembles that of *L. mesomelas*. The carnassial of *L. adusta* is characterized by a reduced trigonid, in mesiodistal length, and an elongated talonid, whereas in MW5-B208 the trigonid makes up the two thirds of the m1. Furthermore, the m1 entoconid of *L. adusta* is not reduced like in MW5-B208. Compared to *L. mesomelas*, MW5-B208 has a more slender m1, a smaller m1 and, particularly, a more reduced m1 talonid characterized by rounded distal corners and reduced lingual side. The occlusal surface of the m2 of *L. adusta* is bean-shaped and enlarged, whereas in *L. mesomelas* it is characterized by a larger mesial (trigonid) portion and a reduced talonid one. The m2 protoconid of both *L. adusta* and *L. mesomelas* is larger when compared to the

metaconid, especially at the base of the cuspid. MW5-B208 possesses m2 protoconid and metaconid of similar size. The distolingual side of the m2 of *L. adusta* is arched with no entoconid, whereas in *L. mesomelas* generally has an entoconid (and some specimens also shows additional accessory cuspulids on this side). Unlike those two species MW5-B208 does not show neither any sign of entoconid nor of a cingulid bounding the distolingual side of the m2.

4.3.2. *Comparison to extant African* Canis *species*

The mandible MW5-B208 differs from that of *Canis lupaster* from northwestern and eastern Africa for the slenderness and shortness of the mandible corpus (Fig. 3o-q). In general, the corpus of the *C. lupaster* is stouter, in both dorsoventral and mediolateral dimension. Moreover, in *C. lupaster*, the ventral side of the corpus is arched, unlike MW5-B208. Although there might be some interalveolar gap between premolars, these spaces cannot be considered real diastemata, for their reduced width and because this feature seems rather variable between individuals. Even dentally the difference between *C. lupaster* and MW5-B208 is evident. The African golden wolf has buccolingually compressed premolars. The p4 has a two prominent accessory cuspulids distal to the protoconid: a stouter one and a tinier but still evident one far distally. The latter is generally separated from the distal cingulid, although in some specimens it is attached to it. In the lower carnassial, unlike MW5-B208, the paraconid of *C. lupaster* is proportionally reduced and the metaconid is more developed and, generally, projecting distally. In the m1 talonid of *C. lupaster* the entoconid is reduced in comparison to the hypoconid but evident, whereas in MW5-B208 this cuspid seems rather reduced. Furthermore, *C. lupaster* generally present an entoconulid on the mesial face of the entoconid, as opposed to the open lingual side of the talonid basin of MW5-B208. The m2 of *C. lupaster* is similar to the morphologies of MW5-B208 yet some differences should be pointed out, for instance, the reduction of the m2 metaconid compared to the m2 protoconid; the large based hypoconid, giving the tooth an occlusal bean-shaped morphology.

4.3.3. *Comparison to selected fossil canids of Africa*

The fossil *Lupulella* sp. from Koobi Fora (2-1.56 Ma, Upper Burgi-KBS: KNM-ER 332 and KNM-ER 895)[45] possess deep mandible corpora (Fig. 3b-c), which are also rather short in rostrocaudal direction and with a scaphoid ventral outline. Moreover, they do not present diastemata between premolars, unlike MW5-B208 from Melka-Wakena (Fig. 2). In terms dental features, Lupulella sp. from Koobi Fora has a buccolingually compressed p4, unlike the more rounded one of Melka Wakena mandible. In turns, the m1 of MW5-B208 is more slender, especially at the level of the protoconid, where KNM-ER 895 is enlarged, both in its m1 protoconid and metaconid. Moreover, the p4 of MW5-B208 shows a very feeble accessory cuspulid distally to the p4 protoconid, unlike the prominent one evident in both KNM-ER 332 and KNM-ER 895. Furthermore, the two specimens from Koobi Fora show a high-crowned, almost pointy distal cingulid on the p4, which is reduced in MW5-B208. The m2 of *Lupulella* sp. is bean-shaped, although the two specimens differ from one another for the development of the buccal cingulid: KNM-ER 895 has a conspicuous enlargement of this cingulid, much more than that of MW5-B208, whereas in KNM-ER 332 the same cingulid is less expanded buccally. Although the metaconid and protoconid are of similar size like in MW5-B208, the talonid portion of the specimens from Koobi Fora differs from that of Melka Wakena for the large and prominent entoconid that occupies half of the talonid basin in both KNM-ER 332 and KNM-ER 895.

In *Lupulella paralius* Geraads, 2011 from the Ma site of Ahl al Oughlam (2.5 Ma), the paratype mandible is rather shallow (Fig. 3e-f), somehow similar to that of Melka Wakena. Nevertheless, there are more differences than similarities between the specimens: for instance, the absence of diastemata in *L. paralius*; the position of the mental foramina of MW5-B208, which in *L. paralius* are located under the interalveolar space p1-p2, the mesial one, and under the distal root of the p3, the distal one; the arched ventral outline of the corpus of *L. paralius*. The two forms differ even in dental morphology. The p4 of *L. paralius* is very compressed buccolingually, as visible in occlusal view, and possesses a prominent distal accessory cuspulid distal to the protoconid and markedly developed distal cingulid, as evident in buccal view. In the lower carnassial, *L. paralius* has a proportionally mesiodistally shorter paraconid compared to the protoconid,

unlike the buccal morphology of MW5-B208, in which the protoconid is slender and similar in mesiodistal length to the paraconid. The m1 talonid of MW5-B208 is more reduced, especially at the level of the entoconid, compared to that of *L. paralius*, which shows a squared occlusal morphology compared to the rounded one of Melka Wakena. Moreover, MNHN.F.AaO-4119 does not show sign of transverse cristid between hypoconid and entoconid, unlike MW5-B208. The m2 of MW5-B208 has a squared occlusal outline whereas *L. paralius* shows an oval shape, in occlusal view. Moreover, in *L. paralius*, the m2 protoconid is prominently developed unlike that of MW5-B208. Both specimens do not possess a m2 entoconid, although the morphology of the talonids in the two taxa differs greatly from one another, particularly for the shape of the distal side of the m2 hypoconid, its smaller proportional size and the deeper m2 talonid basin in MW5-B208 compared to *L. paralius*.

The Middle Pleistocene *Lupulella mohibi* Geraads, 2011[46], for its marked hypocarnivorous dental features, e.g., the mesiodistally short m1 trigonid, enlarged molar areas, etc., are indeed different from MW5-B208 of Melka Wakena (Fig. 3a). Even the mandibular features are distinct between the two taxa. In *L. mohibi* the ventral margin is curved, the larger mental foramen lies below the p2, and the distal one is below the mesial root of the p3, all in contrast with the morphologies of MW5-B208.

In comparison to the much older *Eucyon kuta* from Aralee Issie (Afar Region Ethiopia; 3.82-3.57 Ma)[47] the mandible from Melka Wakena is considerably shallower (Fig. 3g-h), with a straighter ventral outline and marked by evident diastemata between the alveoli of the premolars,. Dentally, the type mandible of *E. kuta* (ARI-VP-1/640) has a stouter canine, larger than that of MW5-B208, a mesiodistally elongated p4 unlike MW5-B208 in which it is shortened and tend to be more rounded, and a larger-based and more robust m1, as opposed to the slender carnassial of MW5-B208.

Compared to the mandible of the type of *Canis brevirostris* Ewer, 1956[48] it is easy to notice the differences that separate MW5-B208 from Melka Wakena from the Sterkfontein canid (Fig. 3d). The mandible of *C. brevirostris* is shortened, like the snout; as a consequence of the shortening there are no diastemata, a clear distinction with MW5-B208[48]. The p4 is high-crowned, with a large-tipped protoconid and a distal cuspulid

closely attached to it. In contrast, the p4 of MW5-B208 is lower-crowned and possesses a reduced distal accessory cuspulid. The m1 and m2 occlusal surfaces are enlarged in *C. brevirostris*, unlike those of MW5-B208, particularly the m1 talonid and the three major cuspids of the m2 are all large-based[42]. In addition to these difference, *C. brevirostris* possesses a m2 paraconid or a cuspulid-like paracristid in the m2 and cuspulids on the lingual side of the m2, although they are rather worn down and it is difficult to assess if those are really entoconid and entoconulid of simple swellings of the enamel of the cristid[48].

4.3.4. *Morphometric analyses and BCA of Melka Wakena mandible*

The results of the principal component analysis are reported in Fig. 4 of the main text. Only the first two components had eigenvalues greater than one (i.e., they explain more variance than any of the original variables considered separately) and jointly account for >85% of the original variance. According to the factor loadings of the variables in these principal components (Supp. Table 1), the first can be interpreted as a size vector, because all variables load positively on this axis, and the second describes a shape vector, with several variables loading positively (for example, the dimensions of the talonid basin in the carnassial and the size of the second molar). Whereas other components show negative loadings (particularly, jaw depth and breadth at several points of the mandible and, to a lesser extent, the size of the trigonid blade in the carnassial). The scatter of the specimens analyzed on the two components (Fig. 4) shows a marked difference between the mesocarnivorous and the hypercarnivorous canids: the jaws of the former are on average of smaller size, as reflected in their negative scores on the first axis, and tend to score more positively on the second axis, which reflects a higher emphasis on the grinding area of the dentition (i.e., the talonid and the second molar); in contrast, the jaws of the hypercarnivores are larger, except in the case of the dhole, and more stoutly built (i.e., the mandibular ramus is shorter and deeper), with a trigonid blade more developed, as expected in these species, which have a diet almost exclusively based on vertebrate flesh from prey of a size similar or greater than the predator and hunted cooperatively.

Supplementary note 5. Results of the modelling analyses

5.1. *The Hindcasting modelling*

The MESS analyses showed that no one of the 5 PC variables of the past time interval had a number of extrapolating pixels higher than the 20% of all the African continent. In details, PC1 variable, on average, has only 0.006% of extrapolating cells (sd: 0.0008), PC2 has no extrapolating cells, PC3 has, on average, 0.001% of extrapolating pixels (sd: 0.001), PC4 has 0.008% (sd: 0.001) and PC5 has, on average, 0.102% of critical pixels (sd: 0.094). For each one of the 5 small models, for the spatial block cross-validation and parameters' tuning procedure, we run a total of 6160 models. Of these, we selected, for any small model, the combinations of parameters' tuning yielding the highest AUC values for the prediction of the testing data. In the end, we had 5 small models with a mean AUC value of 0.965 (sd: 0.032). As regards the variables' importance, the PC2 axis raster resulted as the most important predictive variable in 80% the selected best small models, whereas PC4 was the second most import variable in 60% of models.

5.2. *Estimating the species' habitat status in the past*

During the time interval of the fossil locality age estimate (from 1325 to 1691 ka) the mean HSI value at the site was 0.263 (sd: 0.159), the minimum value was 0.059, recorded at 1,437 ka, whereas the maximum value was 0.769 and recorded at 1657 ka. The HSI values sampled at Melka Wakena site where higher than the 99% of all the HSI values in the whole Ethiopian territory in 100% of the time bins within the fossil site's estimated age range. When we considered the Ethiopian wolf geographic range computation from the present to 2 Ma (all computed by using Raia et al., 2020, bioclimatic variables) the mean value was 35,146.177 km^2, with minimum value of 2,500 km^2 reconstructed for 1077 and 1857 ka, whereas the maximum range size was 240,000 km^2 and was recorded 24 ka, i.e., during the Last Glacial Maximum.

The sets of 2000 Wilcoxon tests showed that the 72.6% of the past time intervals showed climatic conditions in Ethiopia statistically similar to the present territory (mean W: 23.063, W sd: 12.696; mean *p* value: 0.549,

p value sd: 0.346, Fig. 5a), whereas 28.4% of past time bins showed climatic conditions significantly better than the present Ethiopia (mean W: 124.690, W sd: 64.209; mean *p* value: 0.006, *p* value sd: 0.006, Fig. 5a). The variance of HSI in the computed geographic ranges of the species for the time intervals after 1 Ma was significantly higher than the time bins before 1 Ma (HSI variance difference: 0.0001; Brown-Forsythe test for equality of variance: 384.08, $p << 0.0001$). This result confirmed our starting hypothesys according to which the stronger climatic oscillations determind by the ~100 ka glacial cycles made the species' geographic range more variable than the ~41 ka cycles.

As regards the proportional composition of the HSI classes in the territory of Ethiopia, we found that from the present to 2 Ma, the GB class (i.e., the portion of territory bearing HSI values higher than 0.5) had a mean value of 0.095 with minimum value of 0 and recorded for 903 time intervals evenly distributed during the last 2 Ma. The maximum value of GB class portion of territory was 0.5, recorded for 473, 1015, 1016, 1917, 1918 and 1919 ka. As regards GW class (i.e., the portion of territory bearing HSI values equal or lower than 0.5), the mean portion value was 0.904, with minimum value of 0.5 and maximum 1. Since the proportion per time bin of GB and GW are complementary, the temporal intervals during which we recorded the minimum proportion of GW class are the same where we recorded the highest value of GB class. The same applies for the maximum values of GW and the minimum of GB classes. The projection of the Ethiopian wolf niche models onto the present bioclimatic layers[49] provided a proportion of GB class equals to 0 and, inevitably, 1 for the GW class. Since the number of GB and GW classes are complementary (see Methods, section 2.7.1.), we counted the number of temporal intervals with proportion of GB classes higher than recorded for the present. We found that in the last 2 Ma there were 1098 time bins (i.e. 54.9 % of the total) during which the conditions for the species were better than now.

5.3. *The relationship between the species' geographic range evolution and the temperature changes and the species' altitude shift in the past*

As regards the regression analysis between the Log_{10} species' geographic range and mean temperatures and altitudes, the model's residuals showed a clear temporal autocorrelation (Supp. Fig. 4). By following the first strategy to set the ARIMA parameters, maximum likelihood estimation on the OLS residuals time series yielded for the AR component and order of 2, meaning that any value in the series can be predicted by considering the 2 consecutive preceding observations. As described above, we combined this AR component with the I component 1 and the set of MA components ranging from 0 to 5. The second strategy for the calculation of the ARIMA component yielded a combination of the following parameters: AR=2; I=1; MA=2. By considering the two strategies all together, the combination of ARIMA components with the lowest AIC (-5336.057) included the following parameters: AR=2; I=1; MA=4 (Supp. Fig. 5). This ARIMA model yielded a negative and statistically significant relationship between mean temperature and log10 species' geographic range (slope= -0.179, p<<<0.001).

As regards the relationship between mean temperature and the log10 mean altitude, in this case too, the OLS residuals showed a degree of statistically significant autocorrelation. According to the first strategy for ARIMA parameters estimation, the AR component by maximum likelihood estimation for the OLS residuals yielded a value of 12 and this was combined with the I and AM components, as described above. The combined parameters estimation via maximum likelihood method yielded for the AR a value of 2, for I a value of 1 and for MA component a value of 3 (AIC= -11287.72). In the end, we chose as best model the one with the lowest AIC (-11443.68) and including to the following components' values: AR= 12; I= 1; MA=1. This model showed a negative and statistically significant relationship between mean temperature and log10 mean altitude sampled by the species in the last 2 Ma (slope= 0.016, p<<<0.001).

5.4. ***The Forecasting modelling***

Forecasting the fate of such a narrowly distributed species under the current and future global warming is a very challenging task. Yet, researches dealing with this subject are emerging in the scientific community[50]. As seen in the case of past scenarios, the MESS analyses for future temporal intervals also showed that for

all the CMIP6 climatic projections, no one of the selected bioclimatic variables has relevant issues about extrapolating pixels (Supp. Table 2).

The predictor variables selected after ruling out those with a correlation coefficient higher that 0.7 were the “Mean Diurnal Range”, “Isothermality”, the “Mean Temperature of Wettest Quarter”, the “Mean Temperature of Driest Quarter”, the “Precipitation of Wettest Month”, the “Precipitation of Driest Month”, the “Precipitation Seasonality” and the “Precipitation of Coldest Quarter”. Considering all the 28 unique combinations of 6 out of 8 described variables in the small models, for the spatial block cross-validation and parameters’ tuning procedure, we run a total of 15680 models. Then, for any small model, we selected the combinations of parameters with the highest AUC when predicting the spatial block testing data. The mean AUC value of the 28 best small models was 0.985 (sd: 0.025). As regards the variables’ importance, considering the selected 28 best small models, the most frequent most important variables were the Mean Temperature of Driest Quarter (57.14%) and the Mean Temperature of Wettest Quarter (39.29%), whereas the most frequent second most important variables were the Mean Temperature of Wettest Quarter (35.71%), the Mean Diurnal Range and Precipitation of Driest Month (both with 21.43%). The projection of the models onto present climatic conditions is shown in Fig. 6f and the projections onto future time bins are reported in Fig. 6g-j(CMIP6 model BCC-CSM2-MR and ssp 370).

We computed the landscape metrics described in the material and methods section of the main manuscript for each of the four HSI classes, for the present and the cited future temporal bins, by considering the four mentioned CMIP6 future climate projections. The most important metric is the mean patch area, by which all the others are derived. For the present, the HSI class with the highest mean patch area is the class 1 (111.232 km^2), which includes the lowest HSI value. The second most important HSI class is the fourth, bearing the highest HSI values and with 0.030933 km^2; then there are the second (0.014624 km^2) and the third (0.012169 km^2) HSI classes. The other landscape metrics for the present time interval are reported in Supp. Table 3.

In general, as regards the future climate projections, we found that only the first HSI class, i.e. the one with the lowest HSI values, increased in surface area through time (Supp. Table 4) with, for MIROC6 projections, an average increase of 100.36% in 2040, 100.52% in 2060, 100.70% in 2080 and 100.84% in 2100 if compared with the present conditions (see Supp. Table 4 for details). All the other HSI classes decreased drastically with a total disappearance of HSI classes 3 and 4 (with the best HSI values) in 2080 and 2100 with the future climate projections CNRM-CM6-1 and CNRM-ESM2-1, when considering the socio-economic scenario projections with the highest CO_2 emissions rates (ssp 370 and 585). Detailed descriptions of mean patch area for alle the CMIP6 future projections and all the related shared socio-economic pathways are reported in Supp. Table 4.

As regards the differences between the future climatic projection scenarios provided by the CMIP6 models by considering all the landscape metrics we computed (Supp. Table 5), the two-way PerMANOVA test with strata showed statistically significant differences between CMIP6 projections do exist (R2= 0.092, F= 6.502, *p*< 0.001) and even if we take into account the contribution of the landscape metrics' temporal variation (R2= 0.102, F= 21.693, *p*< 0.001). The pairwise version of the two-way PerMANOVA yielded statistically significant differences between MIROC6 and BCC-CSM2-MR projections (F= 4.892, *p*= 0.024), between MIROC6 and CNRM-CM6-1 when considering their values as a whole (R2= 0.143, F= 16.022, *p*= 0.001) also when taking into account the effect of the time (R2= 0.096, F= 10.770 , *p*= 0.001); between MIROC6 and CNRM-ESM2-1 (R2=0.091, F= 9.385, *p*= 0.001) and by accounting for the time effect ((R2=0.088,F= 9.163, *p*= 0.002); between BCC-CSM2-MR vs. CNRM-CM6-1 (R2=0.045,F= 4.621, *p*= 0.029) and by considering the metrics' temporal variation (R2=0.131, F= 13.512, *p*= 0.001); between BCC-CSM2-MR vs. CNRM-ESM2-1 when only considering the effect of the time (R2=0.114, F= 11.064, *p*= 0.002); and between CNRM-CM6-1 vs. CNRM-ESM2-1 models when taking into account how the landscape metrics changed during time (R2=0.213, F= 23.485, *p*= 0.001). The detailed results are provided in Supp. Table 6.

As regards the Linear Regression analyses, when considering the CMIP6 MIROC6, the relationship between Log10 mean patch area (area_mn) and time (from present to 2100 time bin) was negative but not significant,

whereas the relationship with the HSI classes was negative and statistically significant (Adjusted R-squared= 0.524, slope= -1.146, p<< 0.001); when considering the BCC-CSM2-MR future climate projections, the only significant relationship was between area_mn and HSI classes (Adjusted R-squared = 0.557, slope = -1.194, p<< 0.001); with the CNRM-CM6-1 projections, area_mn showed negative and significant relationship with both the time and HSI classes (Adjusted R-squared= 0.512; time: slope= -0.024, p= 0.026; HSI classes: slope= -1.641, p<< 0.001); with the CNRM-ESM2-1 climate projections, area_mn showed a negative and significant relationship with the HSI classes (Adjusted R-squared= 0.479, slope= -1.383, p<< 0.001).

As regards the Generelized Linear Model regressions between the HSI classes and all the landscape metrics in interaction with the time intervals, when considering the MIROC6 future climate projections, the HSI classes showed a positive and significant relationship with the patch aggregation index (ai: slope= 0.0002, p= 0.028); a negative and significant relationship with the patch clumpiness (clumpy: slope= -0.027, p= 0.028) and a positive and significant relationship with the number of patches (np: slope= 0.000001, p= 0.021) with the general model's AIC: 53.545 . When considering both the BCC-CSM2-MR (AIC: 84.58) only the models' intercept was significant (1.142, p= 0.047). With both CNRM-CM6-1 and CNRM-ESM2-1 there was a positive and significant relationship with the number of patches (AIC: 90.33, slope: 0.000002, p= 0.041; AIC: 83.83, slope: 0.000002, p= 0.009, respectively).

Supplementary Figures:

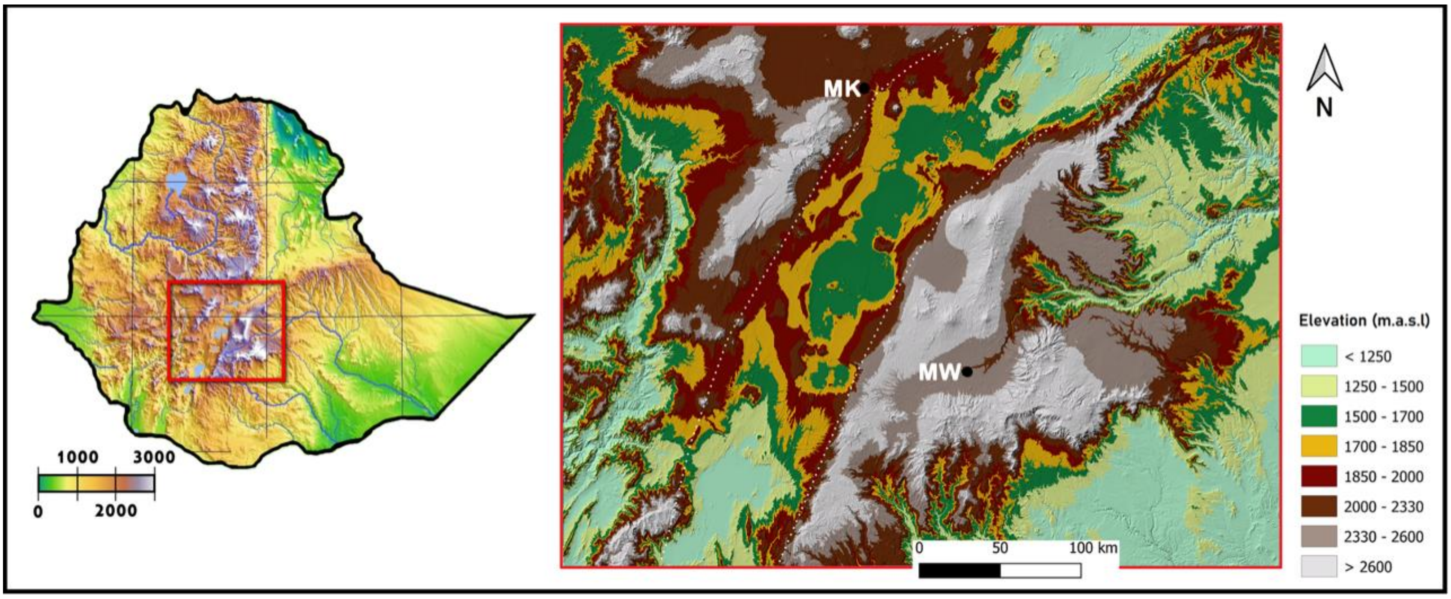

Supplementary Figure 1. Geographic location and topographic elevation of the Early Pleistocene archaeological complexes of Melka Wakena (MW) and Melka Kunture (MK), on the eastern and western highlands, respectively, separated by the Main Ethiopian Rift (MER). The boundaries of the MER are marked by white dotted lines. DEM from USGS National Map Viewer (public domain): http://viewer.nationalmap.gov/viewer/. The map was created by author (T.G) in QGIS.

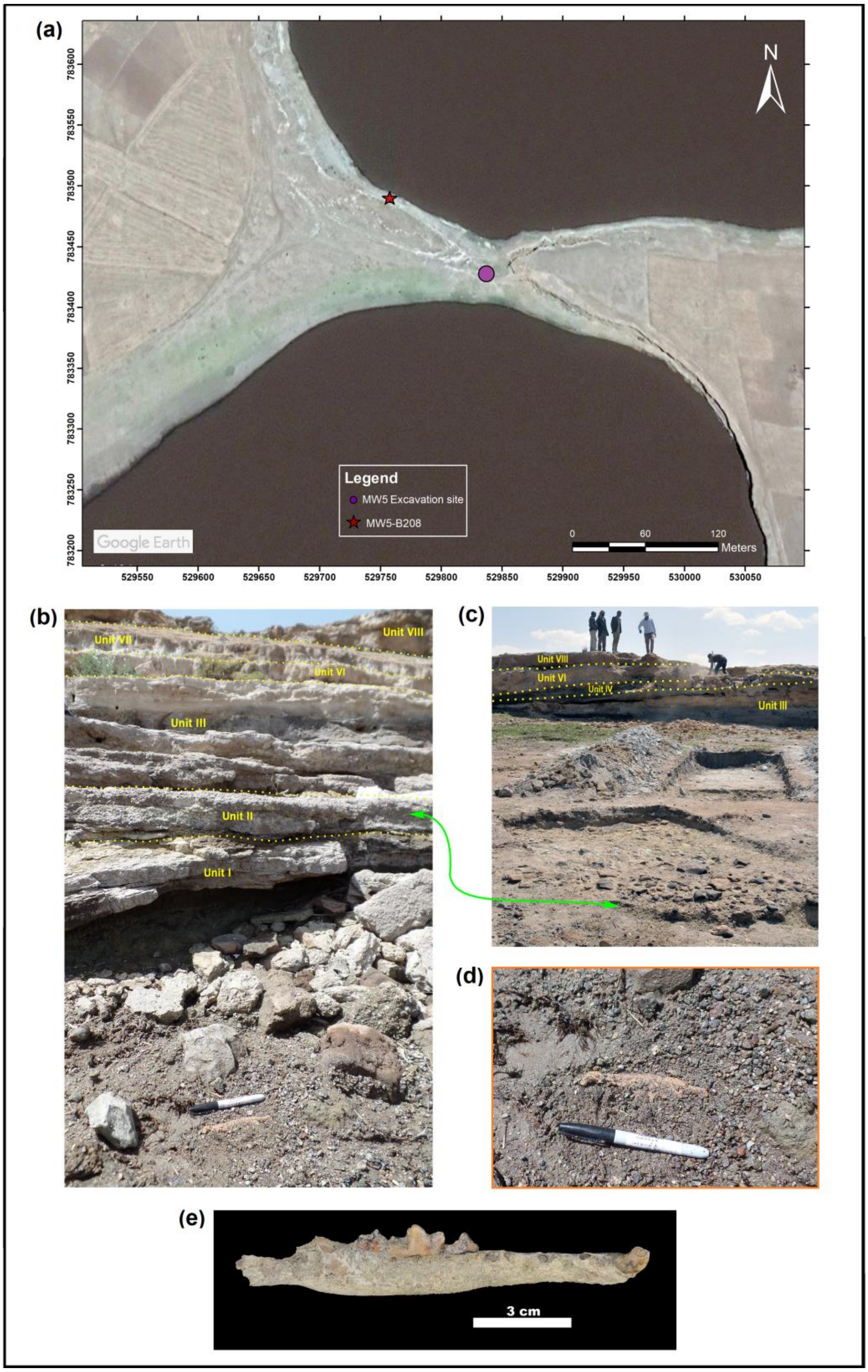

Supplementary Figure 2. (a) Georeferenced map showing the location of the hemimandible (MW5-B208), ca. 90 meters West to the main archaeological site of MW5. Source: https://earth.google.com/web/@7.11707184,39.27041578,-30433.82199405a,33462.79294948d,35y,0.00000277h,5.71535789t,-0r?utm_source=earth7&utm_campaign=vine&hl=en; (b) Location of MW5-B208 in relation to the major stratigraphic units of MW5-West during its discovery in 2017. (c) Field photo showing excavation activities at the main archaeological site of MW5 during the closing stage of the 2017 field work. The green arrow marks the stratigraphic correlation of Unit II at the MW5 sequence with MW5-West. (d) Close-up view of the MW5-B208 in its location during discovery. (e) MW5-B208 before cleaning and curation. Photos 2b-e and stratigraphic correlation created by authors (T. G.).

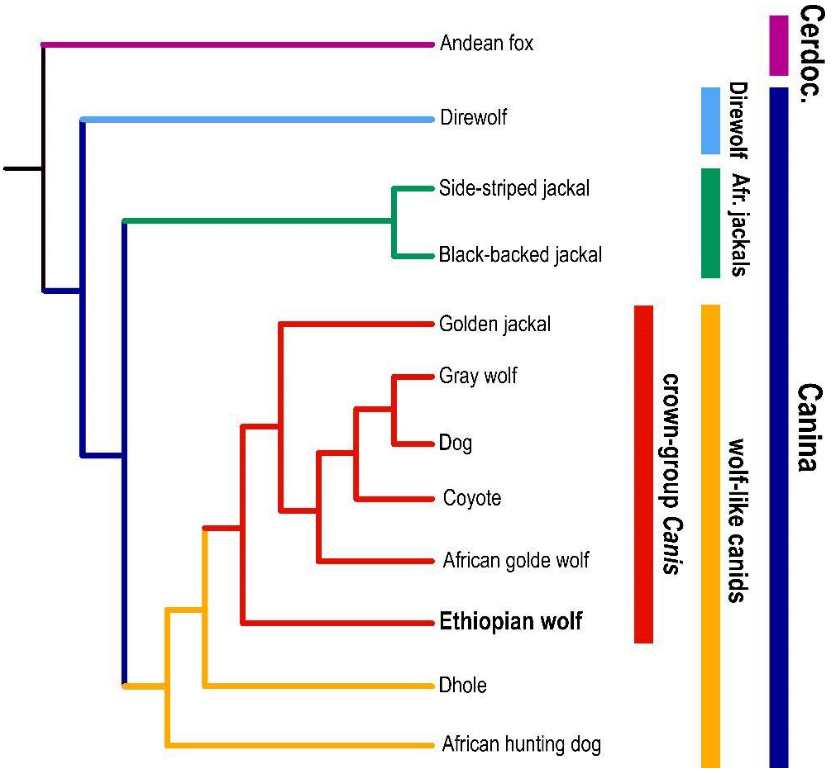

Supplementary Figure 3. Resuming cladogram of molecular relationship of extant and fossil canids compiled from Lindblad-Toh et al.[36], Ciucani et al.[41], Perri et al.[43]. Color code and vertical bars indicate different formal and informal groupings: purple, subtribe Cerdocyonina (sensu Tedford et al.[44]); blue, subtribe Canina; light blue, *Aenocyon dirus*, direwolf, and its lineage; green, clade of African jackals of the genus *Lupulella* (*L. adusta* and *L. mesomelas*); light orange, wolf-like canids (genera *Lycaon*, *Cuon*, and *Canis*); red, species more closely related to *Canis lupus* than to African jackals.

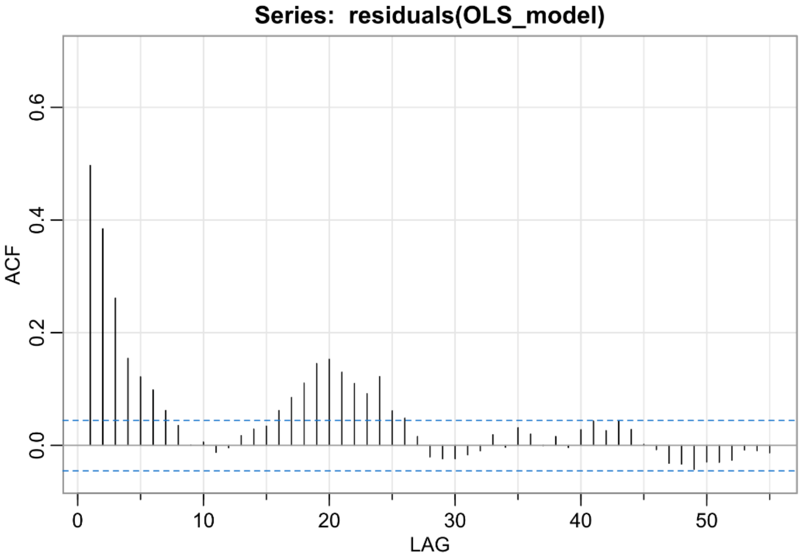

Supplementay Figure 4. Plot showing the temporal autocorrelation in Ordinary Least Square model's residuals. ACF: autocorrelation function, LAG: time step.

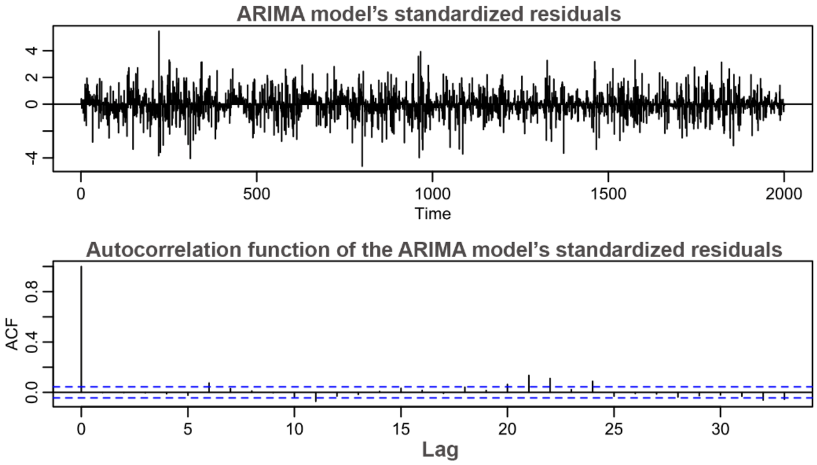

Supplementary Figure 5. Plot showing the ARIMA model's standardized residuals and the related autocorrelation function. ACF: autocorrelation function, LAG: time step.

Supplementary Tables:

Supplemetary Table 1. Measurements (in mm) taken in the mandible and dentition of the fossil specimen MW5-B208 and the extant canid species used for comparison. The specimens were measured by S. Bartolini-Lucenti, B. Figueirido and B. Van Valkenburgh in different museums. MW5-B208 (in bold) was measured by B. Martínez-Navarro in the Ethiopian Heritage Conservation Authority (EHCA), Addis Ababa. In those cases in which a metric variable was not available for a specimen, the mean of this variable in the species was used. Abbreviations of variables: Lc and Bc, anteroposterior length and buccolingual breadth of the lower canine, respectively, measured at the base of the tooth crown; Lp4 and Bp4, length and breadth of the fourth premolar, respectively; Lm1trig, length of the trigonid blade in the lower carnassial; Bm1, maximum breadth of the carnassial; Lm1tal and Bm1tal, length and breadth of the talonid basin in the carnassial, respectively; Lm2 and Bm2, length and breadth of the second molar, respectively; Lcm2, length between the anterior tip of the canine and the posterior border of the second molar; Lm1tal and Bm1tal, height and breadth of the mandible at the posterior border of the canine, respectively; JDp3p4 and JBp3p4, height and breadth of the mandible at the contact between the third and fourth premolars, respectively; JDp4m1 and JBp4m1, height and breadth of the mandible at the contact between the fourth premolar and the carnassial, respectively; JDm1m2 and JBm1m2, height and breadth of the mandible at the contact between the carnassial and the second molar, respectively.

ID	Sex	Species	cL	cB	p4L	p4B	m1 trig L	m1B	m1 tal L	m1 tal B	m2L	m2B	L cm2	JDc	JBc	JD p3 p4	JB p3 p4	JD p4 m1	JB p4 m1	JD m1 m2	JB m1 m2
**MW5-B208**		***C.simensis***	**7.9**	**5.8**	**10.3**	**4.4**	**11.7**	**6.9**	**6.1**	**5.9**	**8.9**	**6.0**	**88.5**	**10.6**	**7.6**	**14.5**	**6.9**	**16.5**	**8.0**	**17.9**	**7.4**
214799		*C. simensis*	8.2	5.2	10.0	5.5	12.6	6.9	6.7	7.7	8.9	6.2	93.5	11.2	16.7	15.8	8.1	16.8	8.3	17.8	7.5
1962-1510		*C. simensis*	10.9	6.4	11.6	5.8	7.3	7.7	7.6	7.5	11.1	6.8	109.8	13.2	17.8	17.2	7.6	19.4	8.3	19.8	7.5
1962-1508		*C. simensis*	10.0	5.7	9.2	4.4	11.8	6.2	6.5	6.1	8.9	5.9	99.0	11.4	14.1	14.9	8.4	15.3	7.8	18.0	7.2
24.8.7.11	m	*C. simensis*	10.4	6.3	10.6	5.1	13.6	6.9	5.8	6.5	9.2	6.1	97.5	12.9	15.1	14.9	7.6	16.3	8.7	16.8	8.7
36.5.20.4	f	*C. simensis*	9.3	5.9	10.1	5.2	13.0	7.0	5.5	6.6	9.2	6.6	96.1	13.4	8.3	16.9	7.3	18.4	7.6	17.1	7.4
23.10.10.1		*C. simensis*	9.1	6.4	11.2	5.4	14.2	7.0	6.2	7.0	9.9	6.6	102.6	14.5	10.3	17.7	7.6	19.1	8.8	19.6	8.4
24.8.7.10	f	*C. simensis*	9.0	5.3	10.1	4.5	12.0	6.4	5.7	6.4	9.1	6.1	96.5	12.4	9.2	14.8	6.7	16.0	7.4	17.5	7.5
2.4.00		*C. simensis*	9.5	6.0	10.3	4.8	13.1	6.6	5.4	6.3	9.0	6.2	99.6	13.4	10.4	17.3	7.6	17.9	8.0	19.2	7.6
24.8.7.12	f	*C. simensis*	9.5	5.0	9.8	4.7	12.2	6.0	5.2	6.2	9.2	6.0	95.8	11.2	9.2	14.8	5.9	14.4	6.4	15.8	6.5
2230		*C. simensis*	9.0	6.2	10.9	4.9	12.5	6.6	6.4	6.6	10.0	6.3	95.8	12.6	12.3	16.5	7.2	20.1	9.8	19.4	8.0
2400	m	*C. simensis*	9.7	6.0	9.8	4.7	12.1	5.9	6.0	6.3	8.9	6.1	90.8	12.6	12.3	16.7	6.5	17.6	7.9	19.0	7.1
221241	f	*C. simensis*	8.6	5.5	10.4	5.0	11.7	6.1	6.0	5.9	9.7	6.3	89.5	12.6	12.3	16.6	7.5	15.3	7.5	16.6	7.5
248710	f	*C. simensis*	8.7	5.4	10.3	4.7	18.1	6.2	6.1	6.3	9.4	6.4	90.8	12.6	12.3	14.9	6.5	16.7	7.5	17.8	7.5
248711	m	*C. simensis*	9.4	6.1	10.7	5.2	13.1	6.5	5.9	6.6	9.3	6.5	87.9	12.6	12.3	15.3	7.4	16.0	8.9	17.5	8.6
248712	f	*C. simensis*	8.5	5.9	10.0	4.7	11.0	5.8	5.7	6.2	9.4	5.9	86.6	12.6	12.3	13.4	5.6	14.8	6.6	15.6	6.2
365204	f	*C. simensis*	8.4	6.0	10.6	5.4	11.3	6.5	6.6	5.6	10.5	6.6	91.9	12.6	12.3	16.7	7.2	17.9	8.4	17.6	7.7
2310101		*C. simensis*	9.8	6.0	11.3	5.5	12.9	6.6	6.1	6.7	10.0	6.6	92.8	12.6	12.3	17.0	7.6	19.7	8.9	19.4	8.4
17800	F	*C. simensis*	9.2	5.4	9.9	4.5	11.9	6.6	6.4	6.5	9.1	6.2	89.6	11.1	8.5	15.7	6.0	15.9	6.7	17.6	6.7
17801	M	*C. simensis*	9.0	5.9	10.3	4.8	12.5	7.0	6.4	7.0	10.0	6.4	90.2	11.4	9.25	14.6	6.8	15.3	7.9	17.0	7.2
818	M	*C. simensis*	9.1	5.9	10.4	4.9	12.6	7.2	6.5	6.9	9.1	6.8	88.3	11.7	8.8	14.8	7.1	15.7	7.8	16.7	7.6
116334	f	*L. adusta*	7.7	4.7	9.7	4.8	9.1	5.7	6.4	6.6	9.1	6.7	78.6	11.4	12.6	12.6	7.7	14.4	7.7	14.9	8.1
52049	m	*L. adusta*	9.8	5.3	8.9	4.3	10.3	6.4	9.3	5.9	7.9	5.5	75.5	12.2	9.5	13.4	6.2	14.1	6.9	15.1	7.1
80662		*L. adusta*	8.1	4.7	10.3	4.5	12.7	7.2	5.3	6.9	9.3	6.9	79.6	11.8	14.2	14.7	7.6	15.7	7.5	16.6	7.9
114174		*L. adusta*	7.6	5.7	10.2	5.0	10.1	6.7	6.8	7.6	8.7	6.4	80.9	13.2	8.7	15.4	7.0	16.3	7.7	17.6	8.4
33322		*L. adusta*	7.6	6.8	12.9	6.1	13.4	8.8	8.6	9.2	8.9	7.0	79.6	13.1	11.3	14.6	10.2	15.4	11.5	15.3	10.5
52057	f	*L. adusta*	7.8	5.3	8.9	4.0	9.0	5.9	5.4	6.1	8.0	6.1	78.3	10.8	8.7	13.1	6.2	14.8	6.6	16.4	6.9
81039	f	*L. adusta*	7.1	5.5	8.9	4.6	9.3	5.9	5.8	6.2	8.1	5.6	73.6	8.9	11.1	11.5	6.9	12.6	7.0	13.7	5.6
116335	f	*L. adusta*	6.7	5.3	10.1	4.7	8.3	7.1	7.1	7.1	8.7	6.4	79.4	10.5	10.6	12.8	6.9	14.9	7.3	15.7	7.3
27725	f	*L. adusta*	6.6	4.3	9.6	4.3	10.4	6.7	5.1	6.4	8.8	6.8	75.1	11.1	12.7	13.0	7.5	13.8	7.3	15.1	7.0
82090	m	*L. adusta*	7.0	5.6	10.5	4.2	8.3	5.9	8.0	7.1	8.5	6.5	74.0	11.7	13.6	13.4	6.2	15.7	6.8	15.7	7.9

52230	m	*L. adusta*	8.2	4.7	9.6	4.6	10.7	7.0	6.9	5.4	7.7	5.9	70.6	11.4	11.3	13.1	7.4	14.0	7.4	15.3	7.3
70661	m	*L. adusta*	7.1	4.9	9.8	4.2	10.5	5.9	5.8	5.4	9.4	5.7	72.3	11.4	11.3	14.4	6.4	15.9	7.6	16.1	6.5
318095	f	*L. adusta*	6.7	4.4	9.3	4.0	9.1	6.0	6.0	6.1	8.1	4.9	72.9	11.4	11.3	11.6	5.8	12.5	6.3	13.0	5.8
341116	f	*L. adusta*	7.2	4.8	10.3	4.4	10.4	6.8	6.3	6.5	9.0	6.2	74.3	11.4	11.3	13.5	6.2	15.4	7.2	14.8	6.4
342085	m	*L. adusta*	7.4	4.7	9.8	4.3	8.5	6.5	6.3	6.1	8.8	5.9	68.8	11.4	11.3	13.5	5.7	15.0	5.9	15.7	5.5
342086	f	*L. adusta*	6.7	4.5	8.6	3.8	7.3	6.0	6.1	5.9	8.0	5.6	66.5	11.4	11.3	12.4	5.0	13.5	5.5	13.9	6.0
671279	f	*L. adusta*	7.4	5.1	11.0	4.7	10.2	6.7	7.0	6.8	9.9	7.1	79.2	11.4	11.3	13.4	6.8	15.4	8.7	16.3	8.4
702327		*L. adusta*	7.9	5.6	10.0	4.4	12.7	6.2	5.9	5.8	9.0	6.3	72.6	11.4	11.3	13.4	5.1	14.5	7.4	14.1	6.6
3591297	m	*L. adusta*	7.6	4.5	10.5	4.3	9.7	6.6	6.9	6.6	9.2	6.7	73.3	11.4	11.3	12.5	6.1	12.9	6.7	13.6	6.7
3591299	m	*L. adusta*	8.2	4.8	10.6	4.5	10.6	6.9	5.5	6.6	9.0	6.4	74.9	11.4	11.3	13.2	6.8	15.4	8.4	15.7	7.5
3591308	m	*L. adusta*	7.0	4.9	10.9	4.6	10.4	7.3	7.4	5.9	10.4	7.1	76.8	11.4	11.3	13.3	7.1	14.6	8.5	16.4	8.1
43144	m	*C. alpinus*	9.1	6.4	12.2	6.3	15.2	8.3	5.7	7.3	7.3	4.9	82.9	16.6	14.1	21.4	9.1	22.8	10.0	23.2	10.1
54544	f	*C. alpinus*	9.9	6.7	11.2	6.0	13.7	9.0	6.0	7.3	7.4	6.4	82.0	15.6	12.6	17.7	9.7	19.9	10.2	21.7	10.0
102083	f	*C. alpinus*	10.0	6.8	11.9	5.5	14.1	7.4	5.3	6.2	6.6	5.9	77.0	15.5	14.3	17.2	11.2	18.4	11.2	21.4	10.6
101773	f	*C. alpinus*	10.3	6.3	10.9	5.4	14.3	7.6	6.0	6.7	7.7	6.1	77.5	15.2	13.4	16.1	10.7	17.7	10.9	19.4	9.8
60775		*C. alpinus*	9.8	6.9	12.6	6.4	14.2	7.5	6.1	7.0	5.9	6.6	83.6	15.5	14.3	19.5	9.3	21.7	9.9	22.1	9.1
34.10.4.2	m	*C. alpinus*	11.3	6.9	12.8	6.2	15.2	8.0	5.3	6.8	7.7	6.1	93.1	11.2	6.8	21.4	11.1	24.0	12.2	26.1	10.1
34.10.4.5	m	*C. alpinus*	10.7	6.6	11.2	5.5	13.7	7.4	4.8	6.2	7.1	6.2	87.0	21.0	13.1	19.7	9.4	21.5	10.3	23.9	10.2
52.4.7.16	f	*C. alpinus*	10.4	5.9	11.4	5.2	13.3	7.2	4.3	6.1	6.9	6.2	78.8	18.7	10.3	19.0	9.7	20.3	10.5	20.5	9.3
52.4.7.17	f	*C. alpinus*	9.6	5.9	12.3	6.1	16.9	8.0	5.2	7.3	6.1	5.9	82.2	20.2	11.9	19.1	9.7	21.8	10.0	21.3	9.2
34.10.4.4	f	*C. alpinus*	10.5	6.6	11.9	5.9	14.7	7.8	4.8	7.3	8.1	6.4	90.2	21.4	10.4	20.4	10.2	21.6	11.0	22.4	9.9
7.11.14.1	m	*C. alpinus*	9.5	6.2	13.2	5.7	16.9	9.4	6.6	7.8	6.9	5.4	84.9	21.0	10.6	20.8	10.7	23.3	11.4	23.9	9.8
341041	m	*C. alpinus*	9.3	6.2	11.5	5.9	14.8	8.2	6.6	6.4	7.7	6.4	82.4	17.4	12.0	20.6	10.0	22.4	11.0	24.9	10.0
341042	m	*C. alpinus*	10.1	6.3	12.6	5.7	14.7	8.3	6.6	6.1	7.4	5.8	86.6	17.4	12.0	20.6	10.7	24.5	12.0	26.2	11.1
341044	f	*C. alpinus*	9.7	6.2	12.4	5.4	14.1	7.9	7.2	6.1	7.9	6.2	81.9	17.4	12.0	19.7	10.2	20.7	10.9	22.4	10.1
341045	m	*C. alpinus*	10.2	6.5	13.1	5.8	14.5	7.9	6.4	5.6	7.1	5.7	81.3	17.4	12.0	19.3	8.7	21.7	10.5	23.7	9.3
524716	f	*C. alpinus*	8.3	5.4	11.6	4.4	13.1	7.8	6.1	5.9	6.8	5.8	75.1	17.4	12.0	18.8	9.0	20.9	10.3	21.1	9.4
524717	f	*C. alpinus*	9.1	5.3	10.5	4.9	11.8	7.5	6.6	5.8	6.5	5.3	74.2	17.4	12.0	18.7	9.1	19.8	10.2	20.7	9.6
711141	m	*C. alpinus*	8.6	5.6	11.6	5.4	13.8	8.4	6.8	6.0	6.6	5.2	81.0	17.4	12.0	20.4	9.7	22.8	10.9	24.0	10.6
2212224	f	*C. alpinus*	10.5	6.5	13.0	6.4	15.1	7.8	6.6	5.7	8.0	5.3	80.9	17.4	12.0	21.6	10.8	22.9	11.6	24.5	10.9
2910194	m	*C. alpinus*	8.9	6.0	12.8	5.2	13.9	7.8	6.3	6.0	6.3	5.1	80.0	17.4	12.0	21.9	8.7	22.0	10.2	22.1	10.1
2910195	f	*C. alpinus*	9.6	6.8	13.0	6.4	15.1	8.2	7.0	5.9	7.2	5.5	82.8	17.4	12.0	21.7	9.3	24.1	11.4	23.0	9.6
27733	f	*C. aureus*	7.3	4.0	9.5	4.1	9.4	6.1	6.7	5.8	8.2	6.0	73.0	11.7	8.7	12.9	6.2	14.3	6.6	14.7	6.5
27732	f	*C. aureus*	7.8	4.4	9.8	4.8	10.6	6.2	6.5	6.4	8.3	5.2	76.7	10.2	12.6	13.0	6.7	14.4	7.1	15.3	6.5
114175	m	*C. aureus*	9.9	5.0	10.7	4.6	11.8	6.8	6.8	6.9	9.1	6.9	82.7	10.8	14.4	12.8	7.5	14.9	7.9	16.7	7.1
100072	f	*C. aureus*	6.3	4.5	9.1	4.3	11.9	6.6	5.5	6.7	8.4	6.5	72.9	11.3	10.5	14.1	6.8	15.8	7.2	16.8	8.4
81040	f	*C. aureus*	6.2	4.2	9.4	4.0	12.1	6.2	5.8	6.5	7.6	5.8	72.9	8.8	9.7	11.1	6.1	13.1	6.8	14.2	6.9
1877144	m	*C. aureus*	7.6	3.9	9.9	4.3	9.4	5.6	6.9	6.0	8.0	5.5	75.5	10.1	9.4	12.7	6.1	13.9	6.6	14.5	6.2
1988.54		*C. aureus*	8.3	5.0	9.9	4.0	11.7	6.5	5.1	6.4	7.6	5.5	78.2	12.6	8.6	13.7	6.6	15.5	8.6	17.6	8.7
14.948		*C. aureus*	8.4	5.0	10.7	4.6	11.8	6.5	6.2	7.1	9.0	6.6	81.5	14.4	8.1	14.9	6.6	15.9	8.2	15.2	7.5
25.4.2.44		*C. aureus*	7.7	5.0	9.5	4.9	10.7	5.9	5.0	6.5	7.3	5.6	69.5	15.4	11.0	15.2	7.7	16.3	8.3	17.3	8.0
4.6.5.4		*C. aureus*	7.1	4.5	9.7	4.3	11.7	6.6	5.4	6.0	7.3	5.9	79.0	11.6	7.4	13.4	6.5	15.1	7.3	15.8	7.0
14479	m	*C. aureus*	6.6	4.3	10.0	4.4	10.3	6.3	6.7	5.5	7.9	5.8	69.9	11.7	10.0	13.3	6.4	14.7	7.5	14.5	7.4
48217	f	*C. aureus*	6.2	4.3	9.3	4.4	10.7	6.1	5.8	6.0	7.3	5.4	63.9	11.7	10.0	11.5	5.1	13.0	5.9	12.6	6.0
70169		*C. aureus*	6.6	3.7	8.3	3.9	10.7	5.8	6.1	5.1	8.1	5.2	61.7	11.7	10.0	11.3	5.8	12.5	6.8	13.5	6.7
231185	m	*C. aureus*	7.2	5.1	9.5	4.9	10.4	6.6	6.3	5.9	8.3	5.8	69.8	11.7	10.0	12.1	5.9	15.7	7.3	15.4	6.7
231187	f	*C. aureus*	7.2	4.6	9.5	4.5	10.1	6.0	6.0	6.0	8.4	5.9	65.9	11.7	10.0	12.7	5.8	14.2	6.9	14.1	6.7
2121128	f	*C. aureus*	6.8	4.3	9.0	4.2	10.2	5.7	5.3	4.8	8.3	5.8	63.7	11.7	10.0	13.1	6.0	14.5	8.2	14.8	6.5
2551216	m	*C. aureus*	8.0	5.3	10.4	4.7	12.0	6.7	6.2	5.4	9.1	5.8	72.7	11.7	10.0	15.4	6.8	15.9	8.0	15.9	6.7
2551217	f	*C. aureus*	7.5	4.5	10.5	4.8	12.0	6.7	6.2	7.2	9.5	5.6	70.3	11.7	10.0	13.6	6.4	15.5	8.1	14.8	7.8
2551218	f	*C. aureus*	8.4	5.2	11.1	5.5	11.7	7.0	6.7	7.0	10.4	6.6	75.7	11.7	10.0	14.0	6.7	17.4	9.1	15.1	8.7

141153	m	*C. latrans*	11.1	5.6	10.5	5.5	9.5	7.8	7.4	7.5	9.1	6.8	94.4	14.4	12.9	14.7	8.5	17.7	9.6	18.3	8.8
141155	m	*C. latrans*	9.8	5.6	12.3	5.5	14.8	7.8	6.6	7.5	9.4	6.8	82.6	13.3	12.9	14.4	8.5	17.3	9.6	20.0	8.8
141162	m	*C. latrans*	10.4	5.5	9.7	5.3	15.8	7.4	7.9	6.9	10.4	6.6	93.5	14.4	14.3	15.7	8.3	18.2	9.2	19.1	8.3
141175	f	*C. latrans*	10.7	4.5	13.4	5.9	17.4	8.9	8.9	8.8	12.1	7.9	94.6	11.4	13.3	13.7	8.4	16.7	9.3	14.1	7.8
141171	f	*C. latrans*	9.2	5.6	11.3	5.5	14.1	7.8	6.8	7.5	9.4	6.8	90.0	13.9	12.9	16.9	8.5	19.1	9.6	19.5	8.8
99653	m	*C. latrans*	10.7	6.0	14.1	6.4	18.2	9.0	7.0	8.8	11.0	8.5	98.2	16.8	13.7	18.7	8.8	22.3	9.6	24.0	8.9
5389	f	*C. latrans*	10.3	5.3	12.2	5.3	13.9	7.2	7.1	6.8	9.3	6.0	91.0	10.9	14.0	14.4	9.3	17.1	11.1	18.9	9.0
131833	m	*C. latrans*	10.8	5.9	12.9	6.5	12.5	8.4	9.2	7.7	8.9	6.8	98.7	13.6	13.5	14.6	9.5	17.0	10.7	18.5	9.4
33653	m	*C. latrans*	10.7	6.3	11.7	6.2	14.1	8.2	6.8	7.5	9.9	6.5	96.4	18.8	13.8	16.1	7.7	17.6	8.9	20.2	8.6
98.12.21.1	f	*C. latrans*	10.2	5.6	12.4	4.7	14.8	7.8	5.9	8.1	9.2	6.4	90.9	15.3	11.1	16.3	8.0	18.6	8.8	18.9	9.4
2.4.2.3	m	*C. latrans*	10.6	5.9	7.9	3.4	8.8	5.2	5.4	5.2	9.2	6.1	97.9	16.0	9.4	17.2	8.3	18.9	9.5	18.9	8.8
33333	f	*C. latrans*	8.3	5.1	11.5	4.6	13.7	7.5	7.6	6.7	10.8	5.5	86.7	14.4	12.9	15.4	7.1	17.9	8.9	19.2	8.2
33430	f	*C. latrans*	8.9	5.9	12.0	5.0	13.5	7.4	7.9	6.6	9.0	5.8	89.5	14.4	12.9	16.0	7.2	18.5	8.4	19.2	7.4
43378	m	*C. latrans*	9.8	6.5	12.3	5.0	14.7	7.6	7.8	7.4	10.0	6.2	92.7	14.4	12.9	16.8	7.7	19.2	9.2	20.1	8.0
43387	m	*C. latrans*	8.6	6.1	11.2	5.3	13.9	8.0	7.8	6.6	9.9	6.5	89.3	14.4	12.9	16.9	7.5	18.7	9.6	19.1	8.6
43390	f	*C. latrans*	8.5	5.2	11.2	4.6	13.5	7.5	7.5	7.0	9.1	5.6	85.4	14.4	12.9	14.9	7.4	16.4	9.0	17.9	8.1
43392	f	*C. latrans*	8.8	5.8	11.5	4.9	13.4	7.8	7.9	7.2	9.3	5.2	88.4	14.4	12.9	16.1	7.7	17.6	8.7	17.9	8.2
43393	f	*C. latrans*	8.1	5.3	10.9	4.9	12.9	7.2	7.0	6.7	8.7	5.6	86.0	14.4	12.9	15.6	7.5	18.3	8.9	18.4	7.8
43399	m	*C. latrans*	9.4	6.3	11.7	5.1	13.1	7.6	7.8	6.8	10.4	6.0	91.5	14.4	12.9	16.5	8.0	19.2	9.0	19.6	8.9
43403	m	*C. latrans*	8.8	5.7	11.8	4.8	13.6	7.3	7.7	6.7	9.6	6.2	88.8	14.4	12.9	17.1	8.3	20.2	8.9	19.7	7.8
43451	m	*C. latrans*	8.5	5.7	10.7	4.5	13.7	7.3	6.6	6.6	9.4	5.5	83.9	14.4	12.9	16.3	7.3	17.7	8.4	18.8	8.1
98227	f	*C. lupus*	16.7	9.9	16.7	7.8	23.3	11.5	8.8	10.4	12.2	9.1	131.2	26.3	18.2	26.7	13.6	29.1	15.6	33.0	15.2
98230	m	*C. lupus*	17.7	11.0	17.6	8.7	23.1	13.0	9.7	11.1	12.3	9.4	138.3	26.5	17.7	29.8	13.6	33.8	15.6	37.1	16.1
98231	m	*C. lupus*	18.1	10.6	16.8	8.5	22.1	12.3	9.0	11.1	12.3	9.2	138.5	28.2	19.9	29.2	17.1	31.8	19.3	34.1	13.7
98226	m	*C. lupus*	18.2	9.9	16.9	7.8	24.0	11.5	8.8	10.4	12.3	9.1	130.2	28.8	18.2	27.3	13.6	30.6	15.6	33.3	15.2
130266	f	*C. lupus*	14.7	9.6	15.4	7.0	20.5	10.9	7.9	10.3	11.7	8.9	111.4	22.8	19.4	23.4	13.4	27.9	14.9	30.9	15.0
134944	f	*C. lupus*	13.5	8.8	14.4	7.0	18.0	10.2	8.6	9.1	11.6	8.5	117.6	24.2	18.2	24.6	13.6	28.4	15.6	27.8	15.2
134942	f	*C. lupus*	16.3	10.9	18.8	9.0	21.3	13.7	12.3	13.6	13.3	12.0	126.9	25.7	21.8	27.0	13.7	30.3	16.5	32.3	18.6
130267	m	*C. lupus*	15.0	9.1	15.9	7.8	20.1	12.1	9.9	10.9	12.9	8.7	126.3	22.8	17.0	23.4	10.8	26.2	13.2	33.0	15.1
24144	f	*C. lupus*	14.8	8.7	13.9	7.5	18.1	10.6	9.1	9.2	11.4	7.9	119.9	24.0	21.0	26.5	14.7	27.4	16.2	28.5	13.1
19.7.15.4		*C. lupus*	18.2	10.3	16.4	7.7	22.7	11.6	7.0	10.4	12.5	9.3	127.9	26.4	17.8	30.6	13.3	33.7	15.0	36.2	17.0
19.7.15.5	m	*C. lupus*	16.1	10.2	17.8	8.6	22.1	11.8	7.6	11.1	11.3	8.8	126.7	28.6	13.4	28.5	13.3	32.3	15.3	30.8	14.7
1938.11.17.1	m	*C. lupus*	16.0	10.1	12.7	6.5	15.4	8.6	5.5	7.2	11.8	8.4	128.0	24.2	15.8	27.2	12.6	31.9	14.1	30.9	13.5
135	f	*C. lupus*	14.0	9.2	15.0	7.2	17.5	11.1	9.8	8.2	11.9	9.3	101.8	25.7	18.2	24.2	11.1	26.9	12.3	26.6	11.2
722	f	*C. lupus*	12.6	8.6	14.2	5.6	17.8	10.7	9.7	8.2	11.8	9.6	102.9	25.7	18.2	24.1	14.7	28.7	14.1	29.7	13.9
753	m	*C. lupus*	16.5	9.9	15.4	7.7	19.5	12.8	10.6	9.4	11.6	8.4	122.2	25.7	18.2	26.3	12.7	29.5	14.8	33.3	15.1
788	m	*C. lupus*	15.5	9.5	15.5	7.6	20.2	11.1	9.8	8.0	11.8	8.3	118.3	25.7	18.2	26.5	13.1	27.7	15.2	27.2	13.6
790	f	*C. lupus*	11.5	7.9	13.8	7.1	15.7	10.4	9.8	8.6	10.7	8.2	103.7	25.7	18.2	24.4	12.8	26.8	13.7	27.6	12.6
1359	m	*C. lupus*	14.0	8.4	14.8	6.9	18.2	10.3	9.2	7.0	10.9	8.3	112.6	25.7	18.2	25.7	12.8	28.9	14.3	31.7	13.7
1499	m	*C. lupus*	15.3	8.0	13.9	6.4	18.2	10.5	9.5	7.7	12.7	8.2	107.1	25.7	18.2	24.4	12.2	26.2	14.2	27.9	13.7
1511	f	*C. lupus*	12.2	7.5	14.7	7.0	16.3	11.3	10.4	8.7	12.5	8.1	109.0	25.7	18.2	26.9	12.9	31.9	15.2	32.2	14.0
1751	f	*C. lupus*	13.4	8.9	13.9	6.6	16.3	10.4	9.2	7.4	11.6	8.7	107.5	25.7	18.2	25.8	11.8	29.7	13.2	29.2	12.2
1964	m	*C. lupus*	15.3	8.8	15.5	7.8	18.7	10.9	9.6	7.6	11.5	8.3	116.3	25.7	18.2	27.3	12.5	30.0	14.3	29.1	13.7
34731	m	*L. mesomelas*	7.6	5.0	9.5	4.5	12.6	6.6	6.6	6.8	7.7	5.9	76.4	10.7	11.2	11.4	7.5	13.0	8.0	14.8	6.0
54209	f	*L. mesomelas*	6.6	4.0	9.3	4.0	10.4	5.9	6.4	5.9	7.0	5.1	72.4	10.8	12.2	14.2	7.7	15.6	7.6	16.3	7.4
187712	m	*L. mesomelas*	6.0	4.4	8.0	4.3	11.4	6.7	5.6	6.1	7.0	5.6	72.4	10.9	10.7	12.8	6.7	14.9	6.7	15.4	6.8
187713	f	*L. mesomelas*	6.6	3.8	8.6	4.4	11.2	6.7	7.0	6.1	6.9	5.6	70.0	8.4	11.0	12.0	7.3	13.3	7.5	14.8	7.2
114228	m	*L. mesomelas*	6.6	4.8	9.7	4.0	10.5	6.4	5.7	6.6	7.3	5.3	72.2	9.5	10.7	13.2	5.8	14.0	7.1	14.5	7.6
27736	f	*L. mesomelas*	6.3	5.0	9.2	4.3	10.6	5.4	4.8	5.9	7.4	5.5	68.9	10.2	9.2	11.5	6.5	13.2	6.5	14.0	6.3

27734	f	*L. mesomelas*	6.8	5.3	9.1	4.3	11.2	6.5	5.5	5.8	7.2	5.7	73.0	10.1	12.0	13.4	7.6	13.8	7.5	15.0	6.7
34732	m	*L. mesomelas*	6.8	4.7	9.1	4.6	12.0	6.3	5.6	6.8	7.7	6.0	74.1	11.1	15.6	14.3	6.8	15.7	7.1	17.0	6.8
27731	m	*L. mesomelas*	7.0	4.4	8.4	4.6	10.9	6.2	5.4	6.1	6.6	5.4	73.2	10.4	11.7	13.0	7.1	14.4	7.4	15.3	7.1
233010		*L. mesomelas*	9.4	4.8	10.5	4.5	13.0	7.7	6.2	7.6	8.6	5.6	75.6	11.9	16.1	14.4	8.0	16.6	8.6	16.5	8.6
99.7.8.3		*L. mesomelas*	7.5	4.4	12.3	5.3	14.9	7.9	7.0	8.0	7.1	5.6	76.6	10.4	8.4	12.5	7.2	14.4	7.3	14.5	7.9
56762	f	*L. mesomelas*	5.5	4.4	9.1	4.0	10.1	6.2	6.0	4.8	7.8	5.1	66.3	10.4	11.7	13.0	6.6	13.8	7.3	14.7	6.9
56763	m	*L. mesomelas*	6.8	3.9	9.4	4.3	10.5	6.8	6.9	5.4	7.7	5.3	69.9	10.4	11.7	12.8	6.2	13.6	7.4	15.8	6.9
99753	m	*L. mesomelas*	7.1	4.8	9.7	4.4	10.8	6.5	6.2	6.6	8.5	5.3	68.5	10.4	11.7	13.3	6.5	14.9	7.4	15.8	7.0
652506		*L. mesomelas*	8.4	4.4	9.6	4.5	10.1	6.7	5.9	5.5	7.6	5.7	66.7	10.4	11.7	12.8	5.9	14.6	7.4	15.2	7.5
2612764	f	*L. mesomelas*	7.7	4.9	10.0	4.0	11.9	6.7	6.4	5.9	8.8	6.0	70.8	10.4	11.7	13.0	5.8	15.3	7.4	16.3	7.3
2612765	m	*L. mesomelas*	8.0	5.4	11.0	4.7	12.1	7.3	6.5	6.9	9.1	6.7	76.0	10.4	11.7	14.2	6.8	15.4	8.0	15.8	7.6
3591315	f	*L. mesomelas*	7.4	4.9	10.1	4.5	10.7	7.6	6.5	6.0	7.9	6.0	69.3	10.4	11.7	11.7	6.8	13.6	7.3	14.0	7.1
28911136	f	*L. mesomelas*	8.4	4.6	10.3	4.2	12.9	6.7	6.1	6.5	8.4	5.9	73.8	10.4	11.7	12.6	5.8	15.8	7.3	14.9	7.3
28911137	m	*L. mesomelas*	8.9	5.5	11.1	5.1	12.7	7.6	6.9	6.6	8.7	6.6	76.9	10.4	11.7	14.8	7.6	17.3	8.5	15.9	8.1
28911141	f	*L. mesomelas*	7.6	4.7	9.4	3.9	11.4	6.3	5.7	5.6	8.0	6.1	65.3	10.4	11.7	11.4	6.2	13.6	6.8	14.1	6.3
85154	m	*L. pictus*	10.4	8.1	13.4	6.6	18.3	9.7	6.1	7.9	9.3	7.8	97.9	21.4	15.7	24.0	11.9	25.6	12.7	28.9	13.0
82087	f	*L. pictus*	11.3	7.8	13.8	5.9	16.8	9.0	6.9	8.1	11.1	6.9	95.6	17.7	16.0	22.6	12.5	24.4	11.8	27.9	12.2
82088	f	*L. pictus*	10.2	8.4	13.0	6.2	16.2	9.3	9.1	8.3	10.8	6.9	98.6	20.4	17.9	23.4	13.4	34.9	13.3	27.3	11.1
114249	m	*L. pictus*	10.4	8.8	14.2	6.8	15.9	11.0	8.6	9.2	10.4	9.3	100.6	20.1	17.2	22.6	12.3	24.5	13.2	28.6	14.7
82086	m	*L. pictus*	10.2	8.5	12.9	6.3	16.6	9.0	8.3	7.6	10.3	7.8	93.5	19.8	14.9	22.6	11.0	23.7	12.1	26.5	13.7
82084	m	*L. pictus*	10.3	8.3	12.3	6.3	18.1	9.8	6.5	8.9	9.5	7.9	92.3	23.4	19.2	24.0	11.6	25.1	12.2	27.0	13.8
82081	f	*L. pictus*	10.2	9.1	13.5	6.6	16.9	9.3	7.4	9.0	10.6	7.2		23.8	15.9	28.0	11.8	28.9	13.5	29.5	15.5
82083	m	*L. pictus*	11.0	9.7	12.4	6.3	17.6	8.6	7.6	8.6	8.9	7.3	95.3	19.9	17.9	22.6	11.3	24.6	12.9	28.3	15.4
114250	f	*L. pictus*	10.1	6.7	11.7	5.7	18.0	9.4	5.6	7.3	9.5	7.2	89.6	17.0	15.7	19.9	11.6	20.6	10.6	24.1	10.7
114251	f	*L. pictus*	10.5	6.0	12.9	6.0	16.5	8.0	6.5	8.0	9.1	7.4	91.6	15.1	16.3	19.3	11.1	21.6	11.1	25.6	11.0
49.121	m	*L. pictus*	12.0	8.2	14.2	6.7	17.5	9.3	5.6	8.5	9.8	7.4	94.8	24.0	14.2	21.0	10.8	21.1	11.9	24.0	11.3
66.643		*L. pictus*	10.7	7.7	9.1	4.2	12.6	6.6	5.0	6.5	9.0	7.3	97.3	26.0	12.6	23.3	11.2	25.2	12.7	26.6	13.2
30587	f	*L. pictus*	9.2	6.7	12.4	5.4	14.5	8.5	7.7	6.3	9.5	6.7	87.8	20.7	16.1	21.9	11.3	23.2	12.4	25.8	12.3
30588	m	*L. pictus*	10.9	7.3	12.6	5.3	15.8	8.9	7.6	6.0	10.0	6.1	90.2	20.7	16.1	22.1	10.6	22.9	10.9	25.1	10.3
75275	m	*L. pictus*	10.7	7.8	13.3	6.4	15.2	9.3	8.6	7.3	9.6	6.7	88.7	20.7	16.1	21.8	10.9	22.9	12.3	25.5	12.3
75276	f	*L. pictus*	10.2	7.0	12.9	6.0	15.0	7.9	7.8	6.4	11.0	7.0	89.2	20.7	16.1	20.1	10.9	21.6	12.7	24.5	12.7
75279	f	*L. pictus*	10.5	7.2	13.2	6.6	15.1	9.0	8.8	6.7	10.3	6.4	90.1	20.7	16.1	22.8	12.1	25.1	13.0	25.6	12.8
301232	m	*L. pictus*	10.3	7.6	13.2	7.0	17.4	9.5	8.9	7.5	11.1	6.9	92.0	20.7	16.1	21.9	11.8	23.5	12.5	26.0	12.9
301233	f	*L. pictus*	10.7	7.6	12.6	6.6	18.2	9.2	7.1	6.8	9.8	7.2	88.2	20.7	16.1	20.8	12.1	23.1	13.1	26.2	13.1

Supplementary Table 2. MESS results. Proportion of raster cell values for each of the used bioclimatic variables in the total territory of Africa with no extrapolating values as computed via Multivariate Environmental Similarity Surface analysis (MESS). The analysis was computed for each of the shared socio-economic pathways (ssp) for the considered future climate models in the time intervals: 2021 – 2040, 2041 – 2060, 2061 – 2080, 2081 – 2100.

**MIROC6**								
**ssp126**								
**Time**	bio2	bio3	bio8	bio9	bio13	bio14	bio15	bio19
**2040**	1.0000	1.0000	0.9967	0.9950	0.9999	1.0000	0.9999	1.0000
**2060**	1.0000	1.0000	0.9933	0.9900	1.0000	1.0000	0.9999	1.0000
**2080**	1.0000	1.0000	0.9928	0.9895	1.0000	1.0000	0.9999	1.0000
**2100**	1.0000	1.0000	0.9936	0.9899	1.0000	1.0000	0.9998	1.0000
**ssp245**								
**Time**	bio2	bio3	bio8	bio9	bio13	bio14	bio15	bio19
**2040**	1.0000	1.0000	0.9970	0.9953	1.0000	1.0000	0.9999	1.0000
**2060**	1.0000	1.0000	0.9914	0.9878	1.0000	1.0000	0.9998	1.0000
**2080**	1.0000	1.0000	0.9868	0.9786	1.0000	1.0000	0.9997	1.0000
**2100**	1.0000	1.0000	0.9827	0.9734	1.0000	1.0000	0.9998	1.0000
**ssp370**								
**Time**	bio2	bio3	bio8	bio9	bio13	bio14	bio15	bio19
**2040**	1.0000	1.0000	0.9998	0.9945	1.0000	1.0000	1.0000	1.0000
**2060**	1.0000	1.0000	0.9896	0.9856	1.0000	1.0000	1.0000	1.0000
**2080**	1.0000	1.0000	0.9814	0.9709	1.0000	1.0000	0.9998	1.0000
**2100**	0.9987	1.0000	0.9648	0.9569	1.0000	1.0000	0.9996	1.0000
**ssp585**								
**Time**	bio2	bio3	bio8	bio9	bio13	bio14	bio15	bio19
**2040**	1.0000	1.0000	0.9956	0.9939	1.0000	1.0000	0.9999	1.0000
**2060**	1.0000	1.0000	0.9860	0.9771	1.0000	1.0000	0.9997	1.0000
**2080**	1.0000	1.0000	0.9695	0.9608	1.0000	1.0000	0.9996	1.0000
**2100**	0.9997	1.0000	0.9343	0.9359	1.0000	1.0000	0.9997	1.0000

**BCC_CSM2_MR**								
**ssp126**								
**Time**	bio2	bio3	bio8	bio9	bio13	bio14	bio15	bio19
**2040**	1.0000	1.0000	0.9954	0.9964	1.0000	1.0000	1.0000	1.0000
**2060**	1.0000	1.0000	0.9932	0.9930	1.0000	1.0000	1.0000	1.0000
**2080**	1.0000	1.0000	0.9930	0.9910	1.0000	1.0000	1.0000	1.0000
**2100**	1.0000	1.0000	0.9942	0.9932	1.0000	1.0000	1.0000	1.0000
**ssp245**								
**Time**	bio2	bio3	bio8	bio9	bio13	bio14	bio15	bio19
**2040**	1.0000	1.0000	0.9952	0.9940	1.0000	1.0000	1.0000	1.0000
**2060**	0.9999	1.0000	0.9912	0.9887	1.0000	1.0000	1.0000	1.0000
**2080**	0.9997	1.0000	0.9866	0.9828	1.0000	1.0000	1.0000	1.0000
**2100**	0.9997	1.0000	0.9816	0.9754	0.9997	1.0000	1.0000	0.9999

**ssp370**								
**Time**	bio2	bio3	bio8	bio9	bio13	bio14	bio15	bio19
**2040**	1.0000	1.0000	0.9963	0.9937	1.0000	1.0000	1.0000	1.0000
**2060**	1.0000	1.0000	0.9856	0.9789	1.0000	1.0000	1.0000	1.0000
**2080**	1.0000	1.0000	0.9731	0.9672	1.0000	1.0000	1.0000	1.0000
**2100**	1.0000	1.0000	0.9454	0.9505	1.0000	1.0000	1.0000	1.0000
**ssp585**								
**Time**	bio2	bio3	bio8	bio9	bio13	bio14	bio15	bio19
**2040**	1.0000	1.0000	0.9949	0.9937	1.0000	1.0000	1.0000	1.0000
**2060**	1.0000	1.0000	0.9838	0.9768	1.0000	1.0000	0.9996	1.0000
**2080**	1.0000	1.0000	0.9610	0.9613	1.0000	1.0000	0.9997	1.0000
**2100**	0.9996	1.0000	0.9343	0.9423	1.0000	1.0000	0.9996	1.0000

**CNRM_CM6_1**								
**ssp126**								
**Time**	bio2	bio3	bio8	bio9	bio13	bio14	bio15	bio19
**2040**	1.0000	1.0000	0.9947	0.9927	1.0000	1.0000	1.0000	1.0000
**2060**	1.0000	1.0000	0.9910	0.9890	0.9999	1.0000	1.0000	1.0000
**2080**	1.0000	1.0000	0.9894	0.9880	1.0000	1.0000	1.0000	1.0000
**2100**	1.0000	1.0000	0.9885	0.9879	1.0000	1.0000	1.0000	1.0000
**ssp245**								
**Time**	bio2	bio3	bio8	bio9	bio13	bio14	bio15	bio19
**2040**	1.0000	1.0000	0.9953	0.9941	0.9998	1.0000	1.0000	1.0000
**2060**	1.0000	1.0000	0.9888	0.9853	0.9999	1.0000	1.0000	1.0000
**2080**	1.0000	1.0000	0.9797	0.9755	0.9999	1.0000	1.0000	1.0000
**2100**	1.0000	1.0000	0.9694	0.9709	0.9999	1.0000	1.0000	1.0000
**ssp370**								
**Time**	bio2	bio3	bio8	bio9	bio13	bio14	bio15	bio19
**2040**	1.0000	1.0000	0.9953	0.9944	0.9998	1.0000	1.0000	1.0000
**2060**	1.0000	1.0000	0.9869	0.9835	0.9999	1.0000	1.0000	1.0000
**2080**	1.0000	1.0000	0.9668	0.9683	0.9999	0.9999	0.9999	1.0000
**2100**	1.0000	1.0000	0.9176	0.9490	0.9999	0.9998	1.0000	1.0000
**ssp585**								
**Time**	bio2	bio3	bio8	bio9	bio13	bio14	bio15	bio19
**2040**	1.0000	1.0000	0.9940	0.9927	0.9998	0.9999	1.0000	1.0000
**2060**	1.0000	1.0000	0.9820	0.9774	0.9999	0.9999	1.0000	1.0000
**2080**	1.0000	1.0000	0.9370	0.9557	0.9999	0.9998	1.0000	1.0000
**2100**	0.9998	1.0000	0.8596	0.9231	0.9999	0.9997	0.9999	1.0000

**CNRM_ESM2_1**

**ssp126**								
**Time**	bio2	bio3	bio8	bio9	bio13	bio14	bio15	bio19
**2040**	1.0000	1.0000	0.9956	0.9954	0.9998	1.0000	1.0000	1.0000
**2060**	1.0000	1.0000	0.9912	0.9883	0.9999	1.0000	1.0000	1.0000
**2080**	1.0000	1.0000	0.9886	0.9862	1.0000	1.0000	1.0000	1.0000
**2100**	1.0000	1.0000	0.9895	0.9867	0.9999	1.0000	1.0000	1.0000
**ssp245**								
**Time**	bio2	bio3	bio8	bio9	bio13	bio14	bio15	bio19
**2040**	1.0000	1.0000	0.9957	0.9948	0.9999	1.0000	1.0000	1.0000
**2060**	1.0000	1.0000	0.9888	0.9849	0.9998	1.0000	1.0000	1.0000
**2080**	1.0000	1.0000	0.9806	0.9757	1.0000	1.0000	1.0000	1.0000
**2100**	1.0000	1.0000	0.9697	0.9695	0.9999	1.0000	1.0000	1.0000
**ssp370**								
**Time**	bio2	bio3	bio8	bio9	bio13	bio14	bio15	bio19
**2040**	1.0000	1.0000	0.9950	0.9953	0.9999	1.0000	1.0000	1.0000
**2060**	1.0000	1.0000	0.9874	0.9829	0.9999	1.0000	1.0000	1.0000
**2080**	1.0000	1.0000	0.9668	0.9661	0.9998	1.0000	1.0000	1.0000
**2100**	1.0000	1.0000	0.9214	0.9454	0.9995	1.0000	1.0000	1.0000
**ssp585**								
**Time**	bio2	bio3	bio8	bio9	bio13	bio14	bio15	bio19
**2040**	1.0000	1.0000	0.9946	0.9935	0.9998	1.0000	1.0000	1.0000
**2060**	1.0000	1.0000	0.9831	0.9781	0.9999	1.0000	1.0000	1.0000
**2080**	1.0000	1.0000	0.9467	0.9588	0.9999	1.0000	1.0000	1.0000
**2100**	1.0000	1.0000	0.8789	0.9303	0.9988	1.0000	1.0000	1.0000

Supplementary Table 3. Landscape metrics computed for the present. HSI class: Habitat Suitability Index classes (see Methods); HSI class: 1 (HSI < 0.25), 2 (HSI ≥ 0.25 and HSI< 0.5), 3 (HSI ≥ 0.5 and HSI< 0.75), 4 (HSI ≥ 0.75). Metrics used: "ai" (patches' aggregation index); "area_mn" (mean patch area); "clumpy" (patches clumpiness); "cohesion" (patches cohesion index); "division" (landscape division index); "np" (number of patches). "area_mn" is expressed in square meters. NA value for mean and standard deviation statistics means that there is only one patch for the specific HSI class.

HSI class	Metric	Value
**1**	ai	99.545
**2**	ai	50.631
**3**	ai	47.234
**4**	ai	78.522
**1**	area_mn	111232000
**2**	area_mn	14624
**3**	area_mn	12.169.697
**4**	area_mn	30.933.333
**1**	clumpy	1.1
**2**	clumpy	0.503
**3**	clumpy	0.47
**4**	clumpy	0.785
**1**	cohesion	99.997
**2**	cohesion	76.32
**3**	cohesion	75.04
**4**	cohesion	83.277
**1**	division	0.027
**2**	division	1
**3**	division	1
**4**	division	1
**1**	np	1
**2**	np	50
**3**	np	33
**4**	np	12
**1**	split	1.027
**2**	split	381.922.579
**3**	split	805.289.797
**4**	split	365.421.139

Supplementary Table 4. Temporal evolution of Habitat Suitability Index classes (HSI classes) surface area for all the CMIP6 future climate models and related shared socio-economic pathways (ssp). Values are reported as percentage of the starting area, i.e. present conditions. HSI class: 1 (HSI < 0.25), 2 (HSI ≥ 0.25 and HSI< 0.5), 3 (HSI ≥ 0.5 and HSI< 0.75), 4 (HSI ≥ 0.75). Percentage values exceeding 100 in a future time intervals indicate an increase of surface area if compared to the starting conditions. NA indicates the total disappearance of HSI class patch.

Model: MIROC6		Starting Area %			
HSI class	**Time**	**ssp 126**	**ssp 245**	**ssp 370**	**ssp 585**
1	**2021-2040**	100.39	100.33	100.34	100.37
1	**2041-2060**	100.43	100.48	100.55	100.62
1	**2061-2080**	100.47	100.68	100.75	100.91
1	**2081-2100**	100.53	100.79	100.93	101.12
2	**2021-2040**	62.86	55.02	69.10	63.42
2	**2041-2060**	70.48	68.22	69.54	81.61
2	**2061-2080**	65.85	76.59	65.34	59.81
2	**2081-2100**	70.16	64.40	61.87	45.95
3	**2021-2040**	59.94	73.08	69.87	65.36
3	**2041-2060**	63.68	75.60	62.67	48.48
3	**2061-2080**	54.91	46.02	67.53	60.81
3	**2081-2100**	67.55	51.99	54.78	52.59
4	**2021-2040**	87.41	70.26	80.69	77.59
4	**2041-2060**	75.52	69.54	70.69	55.86
4	**2061-2080**	85.63	58.05	44.48	39.44
4	**2081-2100**	64.14	51.72	28.74	41.38

Model: BCC-CSM2-MR		**Starting Area %**			
HSI class	**Time**	**ssp 126**	**ssp 245**	**ssp 370**	**ssp 585**
1	**2021-2040**	100.57	100.65	100.65	100.63
1	**2041-2060**	100.68	100.80	100.93	100.93
1	**2061-2080**	100.73	100.91	101.11	101.16
1	**2081-2100**	100.69	100.96	101.20	101.25
2	**2021-2040**	56.85	78.20	88.55	72.56
2	**2041-2060**	76.25	64.32	62.25	57.62
2	**2061-2080**	83.15	67.67	51.42	72.38
2	**2081-2100**	75.65	63.54	63.96	54.70
3	**2021-2040**	73.53	65.23	53.04	50.12
3	**2041-2060**	61.35	47.53	71.00	56.46
3	**2061-2080**	54.17	53.97	61.35	64.09
3	**2081-2100**	45.38	70.12	71.00	83.27
4	**2021-2040**	74.71	74.35	72.41	80.82
4	**2041-2060**	71.12	69.46	36.95	62.07
4	**2061-2080**	59.77	53.45	25.86	18.97
4	**2081-2100**	73.06	29.56	12.93	5.17

Model: CNRM-CM6-1		**Starting Area %**			
HSI class	**Time**	**ssp 126**	**ssp 245**	**ssp 370**	**ssp 585**
1	**2021-2040**	100.69	100.70	100.65	100.72
1	**2041-2060**	100.91	100.95	100.97	101.07
1	**2061-2080**	100.96	101.09	101.19	101.26
1	**2081-2100**	100.95	101.19	101.30	101.34
2	**2021-2040**	70.97	68.45	76.29	68.38
2	**2041-2060**	69.01	67.40	56.59	36.71
2	**2061-2080**	65.65	52.10	57.05	69.75
2	**2081-2100**	62.13	41.33	47.41	21.88
3	**2021-2040**	48.21	46.02	48.35	42.73
3	**2041-2060**	69.24	64.09	65.74	58.73
3	**2061-2080**	71.37	68.37	63.55	46.02

3	**2081-2100**	62.23	65.74	52.59	0
4	**2021-2040**	60.92	65.95	63.79	75.37
4	**2041-2060**	39.44	35.34	37.93	29.74
4	**2061-2080**	34.48	28.45	15.52	0
4	**2081-2100**	37.93	20.69	0	0

Model: CNRM-ESM2-1		**Starting Area %**			
HSI class	**Time**	**ssp 126**	**ssp 245**	**ssp 370**	**ssp 585**
1	**2021-2040**	100.55	100.54	100.48	100.56
1	**2041-2060**	100.84	100.86	100.86	100.99
1	**2061-2080**	100.91	101.07	101.11	101.21
1	**2081-2100**	100.93	101.16	101.25	101.32
2	**2021-2040**	80.06	73.18	70.37	83.15
2	**2041-2060**	57.21	66.35	67.01	46.59
2	**2061-2080**	59.65	42.14	57.58	45.13
2	**2081-2100**	47.95	64.08	45.59	41.03
3	**2021-2040**	55.05	49.75	60.22	60.10
3	**2041-2060**	74.78	69.85	69.02	63.98
3	**2061-2080**	76.25	56.97	47.53	55.22
3	**2081-2100**	66.51	73.25	36.16	0
4	**2021-2040**	68.39	77.59	74.14	65.52
4	**2041-2060**	49.14	43.32	42.12	28.82
4	**2061-2080**	34.73	28.45	16.81	25.86
4	**2081-2100**	36.21	18.10	10.34	0

Supplementary Table 5. Landscape metrics computed for the considered Climate models and all the shared socio-economic pathways (ssp) in the temporal intervals 2021-2040, 2041-2060, 2061-2080 and 2081-2100. HSI class: 1 (HSI < 0.25), 2 (HSI ≥ 0.25 and HSI< 0.5), 3 (HSI ≥ 0.5 and HSI< 0.75), 4 (HSI ≥ 0.75). Metrics used: "ai" (patches' aggregation index); "area_mn" (mean patch area); "clumpy" (patches clumpiness); "cohesion" (patches cohesion index); "division" (the landscape division index); "np" (number of patches). NA value for mean and standard deviation statistics means that there is only one patch for the specific HSI class. NA value for all the other metrics means that no patch exists for that specific HSI class and time interval.

Model: MIROC 6

Time	HSI class	metric	ssp 126	ssp 245	ssp 370	ssp 585
**2021-2040**	1	ai	99.573	99.559	99.565	99.571
**2041-2060**	1	ai	99.577	99.588	99.599	99.616
**2061-2080**	1	ai	99.587	99.626	99.642	99.669
**2081-2100**	1	ai	99.600	99.647	99.673	99.727
**2021-2040**	2	ai	47.426	46.847	47.801	47.759
**2041-2060**	2	ai	48.025	49.000	47.584	52.124
**2061-2080**	2	ai	48.173	51.907	49.000	42.053
**2081-2100**	2	ai	48.288	49.608	43.709	42.282
**2021-2040**	3	ai	42.456	45.714	45.349	45.171
**2041-2060**	3	ai	39.298	41.216	41.985	33.178
**2061-2080**	3	ai	40.000	32.673	37.255	42.308
**2081-2100**	3	ai	38.828	38.065	43.939	37.805
**2021-2040**	4	ai	76.603	73.333	72.474	74.671
**2041-2060**	4	ai	74.532	70.909	72.197	69.744
**2061-2080**	4	ai	74.725	69.061	66.667	71.698
**2081-2100**	4	ai	72.444	68.323	68.235	78.947
**2021-2040**	1	area_mn	111667200.000	111601600.000	111604800.000	111638400.000
**2041-2060**	1	area_mn	111710400.000	111768000.000	111844800.000	111924800.000
**2061-2080**	1	area_mn	111752000.000	111984000.000	112065600.000	112249600.000
**2081-2100**	1	area_mn	111819200.000	112115200.000	112265600.000	112478400.000
**2021-2040**	2	area_mn	9193.220	8045.714	10105.263	9274.576
**2041-2060**	2	area_mn	10307.692	9976.471	10168.889	11935.135
**2061-2080**	2	area_mn	9630.189	11200.000	9555.556	8746.667
**2081-2100**	2	area_mn	10260.870	9417.143	9048.276	6720.000
**2021-2040**	3	area_mn	7294.118	8894.118	8502.857	7954.286
**2041-2060**	3	area_mn	7750.000	9200.000	7626.667	5900.000
**2061-2080**	3	area_mn	6682.353	5600.000	8218.182	7400.000
**2081-2100**	3	area_mn	8220.690	6327.273	6666.667	6400.000
**2021-2040**	4	area_mn	27040.000	21733.333	24960.000	24000.000
**2041-2060**	4	area_mn	23360.000	21511.111	21866.667	17280.000
**2061-2080**	4	area_mn	26488.889	17955.556	13760.000	12200.000
**2081-2100**	4	area_mn	19840.000	16000.000	8888.889	12800.000
**2021-2040**	1	clumpy	1.170	1.147	1.154	1.164
**2041-2060**	1	clumpy	1.181	1.205	1.236	1.284
**2061-2080**	1	clumpy	1.201	1.322	1.387	1.599
**2081-2100**	1	clumpy	1.232	1.427	1.629	2.403
**2021-2040**	2	clumpy	0.472	0.466	0.475	0.475
**2041-2060**	2	clumpy	0.478	0.488	0.474	0.519
**2061-2080**	2	clumpy	0.479	0.517	0.488	0.419

**2081-2100**	2	clumpy	0.481	0.495	0.436	0.422
**2021-2040**	3	clumpy	0.423	0.456	0.452	0.450
**2041-2060**	3	clumpy	0.392	0.411	0.419	0.331
**2061-2080**	3	clumpy	0.399	0.326	0.372	0.422
**2081-2100**	3	clumpy	0.387	0.380	0.439	0.378
**2021-2040**	4	clumpy	0.765	0.733	0.724	0.746
**2041-2060**	4	clumpy	0.745	0.709	0.721	0.697
**2061-2080**	4	clumpy	0.747	0.690	0.666	0.717
**2081-2100**	4	clumpy	0.724	0.683	0.682	0.789
**2021-2040**	1	cohesion	99.998	99.998	99.998	99.998
**2041-2060**	1	cohesion	99.998	99.998	99.999	99.999
**2061-2080**	1	cohesion	99.998	99.999	99.999	99.999
**2081-2100**	1	cohesion	99.998	99.999	99.999	100.000
**2021-2040**	2	cohesion	71.981	67.979	73.821	69.614
**2041-2060**	2	cohesion	70.346	70.361	72.282	79.384
**2061-2080**	2	cohesion	71.733	78.519	70.783	69.474
**2081-2100**	2	cohesion	73.840	68.541	69.903	61.913
**2021-2040**	3	cohesion	68.527	70.196	69.294	69.384
**2041-2060**	3	cohesion	65.236	67.551	62.276	61.850
**2061-2080**	3	cohesion	62.906	58.000	65.292	69.123
**2081-2100**	3	cohesion	69.574	61.849	61.668	58.204
**2021-2040**	4	cohesion	80.985	79.943	80.073	80.487
**2041-2060**	4	cohesion	80.348	79.515	79.823	78.247
**2061-2080**	4	cohesion	80.588	77.882	73.407	69.391
**2081-2100**	4	cohesion	79.786	74.631	65.756	70.189
**2021-2040**	1	division	0.019	0.020	0.020	0.019
**2041-2060**	1	division	0.018	0.017	0.016	0.014
**2061-2080**	1	division	0.017	0.013	0.012	0.008
**2081-2100**	1	division	0.016	0.011	0.008	0.004
**2021-2040**	2	division	1.000	1.000	1.000	1.000
**2041-2060**	2	division	1.000	1.000	1.000	1.000
**2061-2080**	2	division	1.000	1.000	1.000	1.000
**2081-2100**	2	division	1.000	1.000	1.000	1.000
**2021-2040**	3	division	1.000	1.000	1.000	1.000
**2041-2060**	3	division	1.000	1.000	1.000	1.000
**2061-2080**	3	division	1.000	1.000	1.000	1.000
**2081-2100**	3	division	1.000	1.000	1.000	1.000
**2021-2040**	4	division	1.000	1.000	1.000	1.000
**2041-2060**	4	division	1.000	1.000	1.000	1.000
**2061-2080**	4	division	1.000	1.000	1.000	1.000
**2081-2100**	4	division	1.000	1.000	1.000	1.000
**2021-2040**	1	np	1.000	1.000	1.000	1.000
**2041-2060**	1	np	1.000	1.000	1.000	1.000
**2061-2080**	1	np	1.000	1.000	1.000	1.000
**2081-2100**	1	np	1.000	1.000	1.000	1.000
**2021-2040**	2	np	59.000	70.000	57.000	59.000
**2041-2060**	2	np	52.000	51.000	45.000	37.000
**2061-2080**	2	np	53.000	36.000	36.000	30.000
**2081-2100**	2	np	46.000	35.000	29.000	20.000
**2021-2040**	3	np	34.000	34.000	35.000	35.000

**2041-2060**	3	np	32.000	28.000	30.000	32.000
**2061-2080**	3	np	34.000	32.000	22.000	16.000
**2081-2100**	3	np	29.000	22.000	18.000	12.000
**2021-2040**	4	np	10.000	12.000	10.000	11.000
**2041-2060**	4	np	10.000	9.000	9.000	10.000
**2061-2080**	4	np	9.000	9.000	10.000	8.000
**2081-2100**	4	np	10.000	9.000	9.000	3.000

Model: BCC-CSM2-MR

**Time**	**HSI class**	**metric**	**ssp 126**	**ssp 245**	**ssp 370**	**ssp 585**
**2021-2040**	1	ai	99.602	99.625	99.626	99.619
**2041-2060**	1	ai	99.633	99.654	99.668	99.673
**2061-2080**	1	ai	99.637	99.667	99.723	99.737
**2081-2100**	1	ai	99.630	99.680	99.744	99.759
**2021-2040**	2	ai	43.863	46.916	49.072	48.305
**2041-2060**	2	ai	47.319	47.500	41.447	41.724
**2061-2080**	2	ai	51.059	42.532	42.262	49.020
**2081-2100**	2	ai	48.894	41.516	52.239	47.059
**2021-2040**	3	ai	42.238	37.872	31.604	34.389
**2041-2060**	3	ai	38.865	35.714	50.694	44.531
**2061-2080**	3	ai	34.595	44.928	51.546	61.538
**2081-2100**	3	ai	32.642	51.408	69.767	68.966
**2021-2040**	4	ai	74.684	74.519	71.782	75.330
**2041-2060**	4	ai	73.367	73.214	58.824	70.192
**2061-2080**	4	ai	70.053	68.519	58.065	60.000
**2081-2100**	4	ai	75.490	68.657	60.000	NA
**2021-2040**	1	area_mn	111862400.000	111956800.000	111955200.000	111937600.000
**2041-2060**	1	area_mn	111990400.000	112124800.000	112262400.000	112270400.000
**2061-2080**	1	area_mn	112040000.000	112244800.000	112464000.000	112518400.000
**2081-2100**	1	area_mn	111996800.000	112302400.000	112563200.000	112624000.000
**2021-2040**	2	area_mn	8313.725	11435.294	12950.000	10610.526
**2041-2060**	2	area_mn	11151.515	9406.061	9103.448	8426.667
**2061-2080**	2	area_mn	12160.000	9896.296	7520.000	10584.615
**2081-2100**	2	area_mn	11062.857	9292.308	9353.846	8000.000
**2021-2040**	3	area_mn	8948.148	7938.462	6455.172	6100.000
**2041-2060**	3	area_mn	7466.667	5784.615	8640.000	6870.588
**2061-2080**	3	area_mn	6592.000	6568.421	7466.667	7800.000
**2081-2100**	3	area_mn	5522.581	8533.333	8640.000	10133.333
**2021-2040**	4	area_mn	23111.111	23000.000	22400.000	25000.000
**2041-2060**	4	area_mn	22000.000	21485.714	11428.571	19200.000
**2061-2080**	4	area_mn	18488.889	16533.333	8000.000	5866.667
**2081-2100**	4	area_mn	22600.000	9142.857	4000.000	1600.000
**2021-2040**	1	clumpy	1.242	1.303	1.304	1.288
**2041-2060**	1	clumpy	1.329	1.439	1.599	1.621
**2061-2080**	1	clumpy	1.358	1.574	2.267	2.653
**2081-2100**	1	clumpy	1.327	1.683	3.128	4.433
**2021-2040**	2	clumpy	0.437	0.467	0.489	0.481
**2041-2060**	2	clumpy	0.471	0.474	0.413	0.416
**2061-2080**	2	clumpy	0.509	0.424	0.422	0.490

**2081-2100**	2	clumpy	0.487	0.414	0.522	0.470
**2021-2040**	3	clumpy	0.421	0.378	0.315	0.343
**2041-2060**	3	clumpy	0.388	0.356	0.506	0.445
**2061-2080**	3	clumpy	0.345	0.449	0.515	0.615
**2081-2100**	3	clumpy	0.325	0.514	0.698	0.690
**2021-2040**	4	clumpy	0.746	0.745	0.717	0.753
**2041-2060**	4	clumpy	0.733	0.732	0.588	0.702
**2061-2080**	4	clumpy	0.700	0.685	0.581	0.600
**2081-2100**	4	clumpy	0.755	0.686	0.600	NA
**2021-2040**	1	cohesion	99.999	99.999	99.999	99.999
**2041-2060**	1	cohesion	99.999	99.999	99.999	99.999
**2061-2080**	1	cohesion	99.999	99.999	100.000	100.000
**2081-2100**	1	cohesion	99.999	99.999	100.000	100.000
**2021-2040**	2	cohesion	68.100	76.036	79.568	74.606
**2041-2060**	2	cohesion	74.574	68.639	68.884	68.249
**2061-2080**	2	cohesion	78.960	71.196	68.174	70.346
**2081-2100**	2	cohesion	75.243	69.572	68.410	63.556
**2021-2040**	3	cohesion	70.452	67.844	63.862	58.615
**2041-2060**	3	cohesion	65.661	60.321	65.796	61.298
**2061-2080**	3	cohesion	59.136	63.283	66.604	62.850
**2081-2100**	3	cohesion	58.071	69.015	66.127	64.471
**2021-2040**	4	cohesion	80.216	79.816	79.255	80.376
**2041-2060**	4	cohesion	78.896	78.623	67.800	76.282
**2061-2080**	4	cohesion	75.801	76.595	57.471	49.050
**2081-2100**	4	cohesion	79.417	65.309	41.824	0.000
**2021-2040**	1	division	0.015	0.014	0.014	0.014
**2041-2060**	1	division	0.013	0.011	0.008	0.008
**2061-2080**	1	division	0.012	0.009	0.005	0.004
**2081-2100**	1	division	0.013	0.008	0.003	0.002
**2021-2040**	2	division	1.000	1.000	1.000	1.000
**2041-2060**	2	division	1.000	1.000	1.000	1.000
**2061-2080**	2	division	1.000	1.000	1.000	1.000
**2081-2100**	2	division	1.000	1.000	1.000	1.000
**2021-2040**	3	division	1.000	1.000	1.000	1.000
**2041-2060**	3	division	1.000	1.000	1.000	1.000
**2061-2080**	3	division	1.000	1.000	1.000	1.000
**2081-2100**	3	division	1.000	1.000	1.000	1.000
**2021-2040**	4	division	1.000	1.000	1.000	1.000
**2041-2060**	4	division	1.000	1.000	1.000	1.000
**2061-2080**	4	division	1.000	1.000	1.000	1.000
**2081-2100**	4	division	1.000	1.000	1.000	1.000
**2021-2040**	1	np	1.000	1.000	1.000	1.000
**2041-2060**	1	np	1.000	1.000	1.000	1.000
**2061-2080**	1	np	1.000	1.000	1.000	1.000
**2081-2100**	1	np	1.000	1.000	1.000	1.000
**2021-2040**	2	np	51.000	34.000	32.000	38.000
**2041-2060**	2	np	33.000	33.000	29.000	30.000
**2061-2080**	2	np	30.000	27.000	20.000	13.000
**2081-2100**	2	np	35.000	26.000	13.000	10.000
**2021-2040**	3	np	27.000	26.000	29.000	32.000

**2041-2060**	3	np	27.000	26.000	15.000	17.000
**2061-2080**	3	np	25.000	19.000	12.000	8.000
**2081-2100**	3	np	31.000	15.000	5.000	3.000
**2021-2040**	4	np	9.000	8.000	8.000	8.000
**2041-2060**	4	np	8.000	7.000	7.000	5.000
**2061-2080**	4	np	9.000	6.000	4.000	3.000
**2081-2100**	4	np	8.000	7.000	2.000	1.000

Model: CNRM-CM6-1

**Time**	**HSI class**	**metric**	**ssp 126**	**ssp 245**	**ssp 370**	**ssp 585**
**2021-2040**	1	ai	99.629	99.630	99.623	99.635
**2041-2060**	1	ai	99.669	99.677	99.680	99.706
**2061-2080**	1	ai	99.680	99.717	99.742	99.763
**2081-2100**	1	ai	99.677	99.739	99.778	99.795
**2021-2040**	2	ai	52.561	51.316	50.311	50.952
**2041-2060**	2	ai	44.040	39.223	40.364	33.155
**2061-2080**	2	ai	38.909	41.111	49.219	57.471
**2081-2100**	2	ai	39.384	42.017	68.293	100.000
**2021-2040**	3	ai	33.166	32.804	33.495	31.016
**2041-2060**	3	ai	42.857	47.101	47.967	49.573
**2061-2080**	3	ai	47.015	39.326	68.085	75.000
**2081-2100**	3	ai	43.200	72.414	100.000	NA
**2021-2040**	4	ai	70.157	70.492	72.500	71.038
**2041-2060**	4	ai	67.925	71.014	68.919	69.444
**2061-2080**	4	ai	73.134	73.529	71.429	NA
**2081-2100**	4	ai	75.676	100.000	NA	NA
**2021-2040**	1	area_mn	111998400.000	112006400.000	111955200.000	112038400.000
**2041-2060**	1	area_mn	112241600.000	112291200.000	112305600.000	112417600.000
**2061-2080**	1	area_mn	112302400.000	112449600.000	112555200.000	112635200.000
**2081-2100**	1	area_mn	112289600.000	112556800.000	112680000.000	112724800.000
**2021-2040**	2	area_mn	10378.378	10010.256	11156.757	10000.000
**2041-2060**	2	area_mn	10092.308	9856.000	8275.862	5367.742
**2061-2080**	2	area_mn	9600.000	7619.048	8342.857	10200.000
**2081-2100**	2	area_mn	9085.714	6044.444	6933.333	3200.000
**2021-2040**	3	area_mn	5866.667	5600.000	5883.871	5200.000
**2041-2060**	3	area_mn	8426.667	7800.000	8000.000	7146.667
**2061-2080**	3	area_mn	8685.714	8320.000	7733.333	5600.000
**2081-2100**	3	area_mn	7573.333	8000.000	6400.000	NA
**2021-2040**	4	area_mn	18844.444	20400.000	19733.333	23314.286
**2041-2060**	4	area_mn	12200.000	10933.333	11733.333	9200.000
**2061-2080**	4	area_mn	10666.667	8800.000	4800.000	NA
**2081-2100**	4	area_mn	11733.333	6400.000	NA	NA
**2021-2040**	1	clumpy	1.333	1.337	1.306	1.361
**2041-2060**	1	clumpy	1.590	1.675	1.705	2.053
**2061-2080**	1	clumpy	1.702	2.220	3.128	5.203
**2081-2100**	1	clumpy	1.672	3.124	9.493	134.151
**2021-2040**	2	clumpy	0.524	0.511	0.501	0.508
**2041-2060**	2	clumpy	0.439	0.391	0.402	0.331
**2061-2080**	2	clumpy	0.388	0.410	0.492	0.574

**2081-2100**	2	clumpy	0.392	0.420	0.683	1.000
**2021-2040**	3	clumpy	0.331	0.327	0.334	0.309
**2041-2060**	3	clumpy	0.428	0.470	0.479	0.495
**2061-2080**	3	clumpy	0.470	0.393	0.681	0.750
**2081-2100**	3	clumpy	0.431	0.724	1.000	NA
**2021-2040**	4	clumpy	0.701	0.704	0.725	0.710
**2041-2060**	4	clumpy	0.679	0.710	0.689	0.694
**2061-2080**	4	clumpy	0.731	0.735	0.714	NA
**2081-2100**	4	clumpy	0.757	1.000	NA	NA
**2021-2040**	1	cohesion	99.999	99.999	99.999	99.999
**2041-2060**	1	cohesion	99.999	99.999	99.999	99.999
**2061-2080**	1	cohesion	99.999	100.000	100.000	100.000
**2081-2100**	1	cohesion	99.999	100.000	100.000	100.000
**2021-2040**	2	cohesion	75.401	73.673	76.513	72.373
**2041-2060**	2	cohesion	70.815	71.222	66.753	58.158
**2061-2080**	2	cohesion	70.699	64.633	61.734	70.981
**2081-2100**	2	cohesion	68.878	56.669	62.642	29.400
**2021-2040**	3	cohesion	60.242	56.641	59.979	54.908
**2041-2060**	3	cohesion	70.249	70.129	63.800	59.762
**2061-2080**	3	cohesion	71.191	66.009	66.371	48.311
**2081-2100**	3	cohesion	67.360	65.856	50.189	NA
**2021-2040**	4	cohesion	78.892	78.869	78.879	79.278
**2041-2060**	4	cohesion	73.792	67.915	68.401	64.256
**2061-2080**	4	cohesion	67.399	65.001	47.068	NA
**2081-2100**	4	cohesion	69.599	50.189	NA	NA
**2021-2040**	1	division	0.013	0.013	0.014	0.012
**2041-2060**	1	division	0.009	0.008	0.007	0.005
**2061-2080**	1	division	0.008	0.005	0.003	0.002
**2081-2100**	1	division	0.008	0.003	0.001	0.000
**2021-2040**	2	division	1.000	1.000	1.000	1.000
**2041-2060**	2	division	1.000	1.000	1.000	1.000
**2061-2080**	2	division	1.000	1.000	1.000	1.000
**2081-2100**	2	division	1.000	1.000	1.000	1.000
**2021-2040**	3	division	1.000	1.000	1.000	1.000
**2041-2060**	3	division	1.000	1.000	1.000	1.000
**2061-2080**	3	division	1.000	1.000	1.000	1.000
**2081-2100**	3	division	1.000	1.000	1.000	NA
**2021-2040**	4	division	1.000	1.000	1.000	1.000
**2041-2060**	4	division	1.000	1.000	1.000	1.000
**2061-2080**	4	division	1.000	1.000	1.000	NA
**2081-2100**	4	division	1.000	1.000	NA	NA
**2021-2040**	1	np	1.000	1.000	1.000	1.000
**2041-2060**	1	np	1.000	1.000	1.000	1.000
**2061-2080**	1	np	1.000	1.000	1.000	1.000
**2081-2100**	1	np	1.000	1.000	1.000	1.000
**2021-2040**	2	np	37.000	39.000	37.000	36.000
**2041-2060**	2	np	26.000	25.000	29.000	31.000
**2061-2080**	2	np	25.000	21.000	14.000	8.000
**2081-2100**	2	np	28.000	18.000	6.000	1.000
**2021-2040**	3	np	30.000	30.000	31.000	32.000

**2041-2060**	3	np	15.000	16.000	14.000	15.000
**2061-2080**	3	np	14.000	10.000	6.000	2.000
**2081-2100**	3	np	15.000	7.000	1.000	NA
**2021-2040**	4	np	9.000	8.000	9.000	7.000
**2041-2060**	4	np	8.000	6.000	6.000	4.000
**2061-2080**	4	np	6.000	4.000	2.000	NA
**2081-2100**	4	np	6.000	1.000	NA	NA

Model: CNRM-ESM2-1

**Time**	**HSI class**	**metric**	**ssp 126**	**ssp 245**	**ssp 370**	**ssp 585**
**2021-2040**	1	ai	99.599	99.588	99.579	99.599
**2041-2060**	1	ai	99.658	99.659	99.655	99.688
**2061-2080**	1	ai	99.667	99.713	99.723	99.745
**2081-2100**	1	ai	99.672	99.733	99.759	99.784
**2021-2040**	2	ai	50.796	48.325	49.111	52.007
**2041-2060**	2	ai	46.154	44.828	45.604	34.855
**2061-2080**	2	ai	42.949	36.898	43.333	46.087
**2081-2100**	2	ai	39.560	44.828	50.588	54.545
**2021-2040**	3	ai	38.525	36.328	38.077	39.056
**2041-2060**	3	ai	40.741	40.397	45.638	46.875
**2061-2080**	3	ai	49.032	46.018	48.750	68.750
**2081-2100**	3	ai	41.176	63.077	40.000	NA
**2021-2040**	4	ai	71.759	73.394	74.468	71.845
**2041-2060**	4	ai	65.672	66.667	63.265	55.385
**2061-2080**	4	ai	60.000	70.588	55.556	100.000
**2081-2100**	4	ai	59.794	75.000	100.000	NA
**2021-2040**	1	area_mn	111843200.000	111830400.000	111769600.000	111854400.000
**2041-2060**	1	area_mn	112168000.000	112184000.000	112188800.000	112337600.000
**2061-2080**	1	area_mn	112243200.000	112422400.000	112472000.000	112580800.000
**2081-2100**	1	area_mn	112262400.000	112523200.000	112627200.000	112704000.000
**2021-2040**	2	area_mn	11707.317	10702.222	10290.196	12160.000
**2041-2060**	2	area_mn	8365.714	9703.226	9800.000	6812.903
**2061-2080**	2	area_mn	8722.581	6162.963	8421.053	6600.000
**2081-2100**	2	area_mn	7011.765	9371.429	6666.667	6000.000
**2021-2040**	3	area_mn	6700.000	6054.054	7329.032	7314.286
**2041-2060**	3	area_mn	9100.000	8500.000	8400.000	7786.667
**2061-2080**	3	area_mn	9280.000	6933.333	5784.615	6720.000
**2081-2100**	3	area_mn	8094.118	8914.286	4400.000	NA
**2021-2040**	4	area_mn	21155.556	24000.000	22933.333	20266.667
**2041-2060**	4	area_mn	15200.000	13400.000	13028.571	8914.286
**2061-2080**	4	area_mn	10742.857	8800.000	5200.000	8000.000
**2081-2100**	4	area_mn	11200.000	5600.000	3200.000	NA
**2021-2040**	1	clumpy	1.236	1.219	1.195	1.240
**2041-2060**	1	clumpy	1.489	1.505	1.502	1.786
**2061-2080**	1	clumpy	1.587	2.096	2.352	3.514
**2081-2100**	1	clumpy	1.621	2.744	4.830	18.252
**2021-2040**	2	clumpy	0.506	0.481	0.489	0.518
**2041-2060**	2	clumpy	0.460	0.447	0.455	0.347
**2061-2080**	2	clumpy	0.428	0.368	0.433	0.460

**2081-2100**	2	clumpy	0.394	0.448	0.506	0.545
**2021-2040**	3	clumpy	0.384	0.362	0.380	0.389
**2041-2060**	3	clumpy	0.407	0.403	0.456	0.468
**2061-2080**	3	clumpy	0.490	0.460	0.487	0.687
**2081-2100**	3	clumpy	0.411	0.631	0.400	NA
**2021-2040**	4	clumpy	0.717	0.733	0.744	0.718
**2041-2060**	4	clumpy	0.656	0.666	0.632	0.554
**2061-2080**	4	clumpy	0.600	0.706	0.555	1.000
**2081-2100**	4	clumpy	0.598	0.750	1.000	NA
**2021-2040**	1	cohesion	99.999	99.998	99.998	99.999
**2041-2060**	1	cohesion	99.999	99.999	99.999	99.999
**2061-2080**	1	cohesion	99.999	99.999	100.000	100.000
**2081-2100**	1	cohesion	99.999	100.000	100.000	100.000
**2021-2040**	2	cohesion	73.249	72.821	71.452	75.734
**2041-2060**	2	cohesion	69.073	71.142	70.113	62.187
**2061-2080**	2	cohesion	69.180	61.105	65.274	61.643
**2081-2100**	2	cohesion	62.445	68.072	60.528	53.556
**2021-2040**	3	cohesion	59.274	59.563	62.856	60.650
**2041-2060**	3	cohesion	67.628	67.100	68.521	66.630
**2061-2080**	3	cohesion	69.145	62.922	55.162	58.902
**2081-2100**	3	cohesion	64.991	68.101	43.512	NA
**2021-2040**	4	cohesion	79.428	79.186	80.166	79.642
**2041-2060**	4	cohesion	74.327	74.769	73.722	65.691
**2061-2080**	4	cohesion	71.981	62.776	48.469	55.488
**2081-2100**	4	cohesion	72.638	48.311	29.400	NA
**2021-2040**	1	division	0.016	0.016	0.017	0.015
**2041-2060**	1	division	0.010	0.010	0.010	0.007
**2061-2080**	1	division	0.009	0.005	0.005	0.003
**2081-2100**	1	division	0.008	0.004	0.002	0.000
**2021-2040**	2	division	1.000	1.000	1.000	1.000
**2041-2060**	2	division	1.000	1.000	1.000	1.000
**2061-2080**	2	division	1.000	1.000	1.000	1.000
**2081-2100**	2	division	1.000	1.000	1.000	1.000
**2021-2040**	3	division	1.000	1.000	1.000	1.000
**2041-2060**	3	division	1.000	1.000	1.000	1.000
**2061-2080**	3	division	1.000	1.000	1.000	1.000
**2081-2100**	3	division	1.000	1.000	1.000	NA
**2021-2040**	4	division	1.000	1.000	1.000	1.000
**2041-2060**	4	division	1.000	1.000	1.000	1.000
**2061-2080**	4	division	1.000	1.000	1.000	1.000
**2081-2100**	4	division	1.000	1.000	1.000	NA
**2021-2040**	1	np	1.000	1.000	1.000	1.000
**2041-2060**	1	np	1.000	1.000	1.000	1.000
**2061-2080**	1	np	1.000	1.000	1.000	1.000
**2081-2100**	1	np	1.000	1.000	1.000	1.000
**2021-2040**	2	np	41.000	45.000	51.000	40.000
**2041-2060**	2	np	35.000	31.000	32.000	31.000
**2061-2080**	2	np	31.000	27.000	19.000	16.000
**2081-2100**	2	np	34.000	14.000	12.000	4.000
**2021-2040**	3	np	32.000	37.000	31.000	28.000

**2041-2060**	3	np	16.000	16.000	16.000	15.000
**2061-2080**	3	np	15.000	15.000	13.000	5.000
**2081-2100**	3	np	17.000	7.000	4.000	NA
**2021-2040**	4	np	9.000	8.000	9.000	9.000
**2041-2060**	4	np	8.000	8.000	7.000	7.000
**2061-2080**	4	np	7.000	4.000	4.000	1.000
**2081-2100**	4	np	8.000	2.000	1.000	NA

Supplementary Table 6. Two-way pairwise PerMANOVA test results. These results come after the detected significant difference between future CMIP6 projection bioclimatic models when taking into account the temporal variation (time) of the landscape metrics and the different shared socio-economic pathways (ssp) as repeated experiments. CMIP6: pairwise test statistics when considering landscape metrics as a whole in the models; time: test statistics when considering the temporal evolution of the landscape metrics within each CMIP6 model.

MIROC6 vs. BCC-CSM2-MR

	Df	SumOfSqs	R^2	F	Pr(>F)
CMIP6	1.000	0.715	0.053	4.892	0.024
Tme	1.000	0.469	0.035	3.213	0.062
Residual	85.000	12.415	0.913		
Total	87.000	13.599	1.000		

MIROC6 vs. CNRM-CM6-1

	Df	SumOfSqs	R^2	F	Pr(>F)
CMIP6	1.000	2.995	0.143	16.022	0.001
Tme	1.000	2.013	0.096	10.770	0.001
Residual	85.000	15.889	0.760		
Total	87.000	20.897	1.000		

MIROC6 vs. CNRM-ESM2-1

	Df	SumOfSqs	R^2	F	Pr(>F)
CMIP6	1.000	1.370	0.091	9.385	0.001
Tme	1.000	1.338	0.088	9.163	0.002
Residual	85.000	12.409	0.821		
Total	87.000	15.117	1.000		

BCC-CSM2-MR vs. CNRM-CM6-1

	Df	SumOfSqs	R^2	F	Pr(>F)
CMIP6	1.000	0.853	0.045	4.621	0.029
Tme	1.000	2.493	0.131	13.512	0.001
Residual	85.000	15.683	0.824		
Total	87.000	19.029	1.000		

BCC-CSM2-MR vs. CNRM-ESM2-1

	Df	SumOfSqs	R^2	F	Pr(>F)
CMIP6	1.000	0.174	0.012	1.191	0.306
Tme	1.000	1.615	0.114	11.064	0.002
Residual	85.000	12.406	0.874		
Total	87.000	14.195	1.000		

CNRM-CM6-1 vs. CNRM-ESM2-1

	Df	SumOfSqs	R^2	F	Pr(>F)
CMIP6	1.000	0.357	0.018	2.036	0.145
Tme	1.000	4.122	0.213	23.485	0.001
Residual	85.000	14.917	0.769		
Total	87.000	19.396	1.000		

References:

1. Hovers, E. et. al. The expansion of the Acheulian to the Southeastern Ethiopian Highlands: Insights from the new early Pleistocene site-complex of Melka Wakena. *Quat. Sci. Rev*., 253, 106763 (2021).

2. Resom, A., Asrat, A. Gossa, T. & Hovers, E. Petrogenesis and depositional history of felsic pyroclastic rocks from the Melka Wakena archaeological site-complex in South central Ethiopia. *J. Afr. Earth Sci*. **142**, 93-111 (2018). https://doi.org/10.1016/j.jafrearsci.2018.03.003.

3. Sembroni, A. & Molin, P. Long-term drainage system evolution in the Wabe Shebele River basin (SE Ethiopia - SW Somalia). *Geomorphology* **320**, 45-63 (2018). https://doi.org/10.1016/j.geomorph.2018.08.001.

4. Xue, L., Alemu, T., Gani, N. D. & Abdelsalam, M. G. Spatial and temporal variation of tectonic uplift in the southeastern Ethiopian Plateau from morphotectonic analysis. *Geomorphology* 309, 98-111 (2018).

5. Zanettin, B., Justin-Visentin, E., Nicoletti, M. & Petrucciani, C. Evolution of the chencha escarpment and the ganjiuli graben (lake abaya) in the southern Ethiopian rift. *N. J. Geol. Palaont. Monats.* **8**, 473-490 (1978).

6. WoldeGabriel, G., Aronson, J. & Walter, R. C. Geology, geochronology, and rift basin development in the central sector of the Main Ethiopia Rift. GSA Bulletin 102, 439-458 (1990).

7. Corti, G. Continental rift evolution: from rift initiation to incipient break-up in the Main Ethiopian Rift, East Africa. *Earth Sci. Rev.* **96**, 1-53 (2009). https://doi.org/10.1016/j.earscirev.2009.06.005

8. Teklemariam, M. Water-Rock Interaction Processes in the Aluto-Langano Geothermal Field, Ethiopia. Unpublished PhD Thesis. University of Pisa, Italy (1996).

9. Chernet, T., Hart, W. H., Aronson, J. L. & Walter, R.C. New age constraints on the timing of volcanism and tectonism in the northern Main Ethiopian Riftesouthern Afar transition zone (Ethiopia). *J. Volcanol. Geoth. Res.* **80**, 267-280 (1998). https://doi.org/10.1016/S0377-0273(97)00035-8

10. Kazmin, V. & Berhe, S. M. Geological Map of the Ethiopian Rift (1:500,000). Geological Survey of Ethiopia (Unpublished Map) (1981).

11. Nicholson, S. E. A review of climate dynamics and climate variability in Eastern Africa. In: Johnson, T.C., Odada, E.O. (Eds.), The Limnology, Climatology and Paleoclimatology of the East African Lakes, the International Decade for the East African Lakes (IDEAL). Gordon and Breach, Newark, NJ, pp. 25-56 (1996).

12. Kingdon, J. Island Africa:The Evolution of Africa's Animals and Plants. Harper Collins Publishers, New York City (1989).

13. Mairal, M. et al. Geographic barriers and Pleistocene climate change shaped patterns of genetic variation in the Eastern Afromontane biodiversity hotspot. *Sci. Rep*. **7**, 45749 (2017). https://doi.org/10.1038/srep45749

14. White, F. The Vegetation of Africa. A Descriptive Memoir to Accompany the Unesco/AETFAT/UNSO vegetation map of Africa. UNESCO (2014).

15. Bonnefille, R., Melis, R. & Mussi, M. Variability in the Mountain Environment at Melka Kunture Archaeological Site, Ethiopia, During the Early Pleistocene (~1.7 Ma) and the Mid-Pleistocene Transition (0.9–0.6 Ma). In: R. Gallotti, M. Mussi (eds) The Emergence of the Acheulean in East Africa and Beyond. Vertebrate Paleobiology and Paleoanthropology. Springer, Cham. (2018). https://doi.org/10.1007/978-3-319-75985-2_5

16. Bonnefille, R. Implications of pollen assemblage from the Koobi Fora formation, East Rudolf, Kenya. *Nature* **264**, 403-407 (1976).

17. Bonnefille, R., Lobreau, D. & Riollet G. Fossil Pollen of Ximenia (Olacaceae) in the Lower Pleistocene of Olduvai, Tanzania: Palaeocological Implications. *J. Biogeogr.* **9**, 469-486 (1982).

18. Renne, P. R., Balco, G., Ludwig, K. R., Mundil, R. & Min, K. Response to the comment by W.H. Schwarz et al. on "Joint determination of 40K decay constants and 40Ar*/40K for the Fish Canyon sanidine standard,

and improved accuracy for 40Ar/39Ar geochronology" by P.R. Renne et al. (2010). *Geochem. Cosmochim. Acta* **75**, 5097-5100 (2011). https://doi.org/10.1016/j.gca.2011.06.021

19. Niespolo, E. M., Rutte, D., Deino, A. L. & Renne, P. R. Intercalibration and age of the Alder Creek sanidine $^{40}Ar/^{39}Ar$ standard. *Quat. Geochr.* **39**, 205-213 (2017). https://doi.org/10.1016/j.quageo.2016.09.004

20. Gossa, T. The Melka Wakena Site Complex, South-Central Ethiopia: Lithic Technology, Raw Material Economy, and Regional Perspectives on the Early Acheulian on the Ethiopian Highlands. Unpublished Ph.D. dissertation. The Institue of Archaeology, The Hebrew University of Jerusalem (2020).

21. Gossa, T. & Hovers, E. Continuity and change in lithic techno-economy of the early Acheulian on the Ethiopian highland: a case study from locality MW2; the Melka Wakena site-complex. PLoSONE 17(12): e0277029 (2022). https://doi.org/10.1371/journal.pone.0277029

22. Kurashina, H. An Examination of Prehistoric Lithic Technology in East-Central Africa. University MIcrofilm International, Ann Arbor (1978).

96. Clark, J. D. & Kurashina H. Hominid occupation of the east-central highlands of Ethiopia in the plio-pleistocene. *Nature* **282**, 33-39 (1979).

23. de la Torre, I., Mora, R. & Martínez-Moreno, J. The early Acheulean in Peninj (lake natron, Tanzania). *J. Anthropol. Archaeol.* **27**, 244-264 (2008). https://doi.org/10.1016/j.jaa.2007.12.001

24. Beyene, Y. et al. The characteristics and chronology of the earliest Acheulean at Konso, Ethiopia. *Proc. Natl. Acad. Sci. U.S.A.* **110**, 1584e1591 (2013). https://doi.org/10.1073/pnas.1221285110

25. Gallotti, R. An older origin for the Acheulean at Melka kunture (upper Awash, Ethiopia): techno-economic behaviours at Garba IVD. *J. Hum. Evol.* **65**, 520-594 (2013). https://doi.org/10.1016/j.jhevol.2013.07.001.

26. Diez-Martín, F. et al. Early Acheulean technology at Es2-lepolosi (ancient MHS-bayasi) in Peninj (lake natron, Tanzania). *Quat. Int.* **322-323**, 209-236 (2014). https://doi.org/10.1016/j.quaint.2013.08.053

27. Díez-Martín, F. et al. The origin of the Acheulean: the 1.7 million-year-old site of FLK west, Olduvai Gorge (Tanzania). *Sci. Rep*. **5**, 17839 (2015).

28. Santonja, M. et al. Technological strategies and the economy of raw materials in the TK (Thiongo Korongo) lower occupation, Bed II, Olduvai Gorge, Tanzania. *Quat. Int*. **322-323**, 181-208 (2014).

29. Sánchez-Yustos, P. et al. Techno-economic human behavior in a context of recurrent megafaunal exploitation at 1.3 Ma. Evidence from BK4b (Upper Bed II, Olduvai Gorge, Tanzania). *J. Arch. Sci.: Rep*. **9**, 386-404 (2016).

30. de la Torre, I. & Mora, R. Technological behaviour in the early acheulean of EFHR (Olduvai Gorge, Tanzania). *J. Hum. Evol.* **120**, 329-377 (2018). https://doi.org/10.1016/j.jhevol.2018.01.003

31. Presnyakova, D. et al. Site fragmentation, hominin mobility and LCT variability reflected in the early Acheulean record of the Okote Member, at Koobi Fora, Kenya. *J. Hum. Evol*. 125, 159-180 (2018). https://doi.org/10.1016/j.jhevol.2018.07.008.

32. Semaw, S., Rogers, M. J., Cáceres, I., Stout, D. & Leiss, A. C. The Early Acheulean ~1.6–1.2 Ma from Gona, Ethiopia: Issues related to the Emergence of the Acheulean in Africa. In: Gallotti, R., Mussi, M. (Eds.), The Emergence of the Acheulean in East Africa and Beyond: Contributions in Honor of Jean Chavaillon*.* Vertebrate Paleobiology and Paleoanthropology, Springer International Publishing, pp. 115-127 (2018).

33. Mussi, M. et al. After the emergence of the Acheulean at Melka Kunture (Upper Awash, Ethiopia): From Gombore IB (1.6 Ma) to Gombore Iγ (1.4 Ma), Gombore Iδ (1.3 Ma) and Gombore II OAM Test Pit C (1.2 Ma). *Quat. Int*. in press (2021) https://doi.org/10.1016/j.quaint.2021.02.031.

34. Resom, A. Petrogenetic Evolution of the Melka Wakena Pyroclastic Deposits: Implicaitons for the Depositional History of the Intercalated Volcano-Sedimentary Rocks. Unpublished MA thesis. School of Earth Sciences, Addis Ababa University (2017).

35. Gottelli, D. et al. Molecular genetics of the most endangered canid: the Ethiopian wolf *Canis simensis. Mol. Ecol.* **3**, 301-312 (1994).

36. Lindblad-Toh, K. et al. Genome sequence, comparative analysis, and haplotype structure of the domestic dog. *Nature* **438**, 803–819 (2005).

37. Gopalakrishnan, S. et al. Interspecific gene flow shaped the evolution of the genus Canis. *Curr. Biol.* **28**, 3441-3449. (2018).

38. Rueness, E. K. et al. The cryptic African wolf: *Canis aureus lupaster* is not a golden jackal and is not endemic to Egypt. *PLoS One* **6**, e16385 (2011).

39. Gaubert, P. et al. Reviving the African wolf *Canis lupus lupaster* in North and West Africa: a mitochondrial lineage ranging more than 6,000 km wide. *PLoS One* **7**, e42740 (2012).

40. Koepfli, K. P. et al. Genome–wide evidence reveals that African and Eurasian golden jackals are distinct species. *Curr. Biol*. **25**, 2158–2165 (2015).

41. Ciucani, M. M. et al. Evolutionary history of the extinct Sardinian dhole. *Curr. Biol.* **31**, 5571-5579.e6 (2021). https://doi.org/10.1016/j.cub.2021.09.059

42. Wang, X. & Tedford, R. H. Dogs, their fossil relatives and evolutionary history. Columbia University Press (2008).

43. Perri, A. R. et al. Dire wolves were the last of an ancient NewWorld canid lineage. *Nature* **591**, 87-91 (2021).

44. Tedford, R. H., Wang, X. & Taylor, B. E. Phylogenetic systematics of the North American fossil caninae (Carnivora: Canidae). *Bull. Amer. Mus. Nat. Hist.* 2009, 1-218 (2009).

45. Werdelin, L. & Lewis, M. E. Koobi Fora research project, volume 7, the Carnivora. California Academy of Sciences 333 p (2013).

46. Geraads, D. A revision of the fossil Canidae (Mammalia) of North-western Africa. *Palaeontology* **54**, 429-446 (2011).

47. Werdelin, L., Lewis, M. E. & Haile-Selassie, Y. New species of Eucyon (Mammalia; Carnivora; Canidae) from the Pliocene of the Woranso-Mille Area, Afar Region, Ethiopia, and a critical review of African species of *Eucyon*. *Papers in Palaeontology* (2015).

48. Ewer, R. F. The fossil carnivores of the Transvaal caves: Canidae. Proc. Zool. Soc. *London* **126**, 97-120 (1956).

49. Carotenuto, F. et al. MInOSSE: A new method to reconstruct geographic ranges of fossil species. *Meth. Ecol. Evol.* **11**, 1121-1132 (2020).

50. Ahmed, A. S., Kufa, C. A., Atickem, A., Yihune, M. & Bekele. A. Habitat suitability and distribution of endangered Ethiopian wolf (*Canis simensis* Ruppell 1840) and the potential effects of climate change on its habitat in the Ethiopian highlands. (2022). DOI: https://doi.org/10.21203/rs.3.rs-1618917/v1
